# CMTM6 as a potential therapy target is associated with immunological tumor microenvironment and can promote migration and invasion in pancreatic adenocarcinoma

**DOI:** 10.1007/s10142-023-01235-5

**Published:** 2023-09-20

**Authors:** Hongli Gao, Jianqiao Yin, Xin Guan, Shuang Zhang, Songlin Peng, Xun Liu, Fei Xing

**Affiliations:** 1 Department of Oncology, Shengjing Hospital of China Medical University, Shenyang, 110004 China; 2 Department of Gastroenterology, Shengjing Hospital of China Medical University, Shenyang, 110004 China; 3 Department of General Surgery, Shengjing Hospital of China Medical University, Shenyang, 110004 China; 4 Department of General Surgery, the Seventh Affiliated Hospital of Sun Yat-sen University, Shenzhen, 518107 China

**Keywords:** CMTM6, Pancreatic adenocarcinoma, PD-L1, Prognosis, Mutation, Tumor microenvironment, Immune infiltration, Inflammatory activity

## Abstract

CMTM6 has been connected to the development of several malignancies. However, it is still unknown what function CMTM6 serves in pancreatic adenocarcinoma (PAAD). We obtained RNA sequencing information of PAAD from public datasets and predicted statistical significance of CMTM6 survival in accordance with Kaplan–Meier curves. Gene set enrichment assessment (GSEA) was employed to analyze changes in pathways. Then, we systematically investigated the association involving CMTM6 and the immunological traits within the tumor microenvironment (TME) of PAAD, including immune pathways, immunomodulators, immune infiltrating cells, inflammatory activities, and immunotherapy response prediction. To demonstrate the biologically malignant properties of CMTM6 expression, the Cell Counting Kit-8, transwell experiments, colony formation, and wound healing were utilized. Upregulated CMTM6 expression was revealed within PAAD tissues, which was associated with more frequent somatic mutations and worse survival outcomes. Specifically, CMTM6 expression represented stronger immune infiltration, inflammatory activity, and better immunotherapeutic response in TME. Functional studies revealed that CMTM6 promoted the ability to proliferate, migrate, and invade. Additionally, CMTM6 and PD-L1 had a positive relationship, and CMTM6 can co-immunocoprecipitate with PD-L1 protein in pancreatic cell lines. CMTM6 overexpression shapes the inflammatory TME with a strong immune response. These findings support that CMTM6 is an immunotherapeutic target with promising effect to treat PAAD.

**Supplementary Information:**

The online version contains supplementary material available at 10.1007/s10142-023-01235-5.

## Introduction

Pancreatic cancer ranks as the fourth most common cause of cancer-related deaths worldwide. It accounts for approximately 5% of all fatalities associated with cancer (Bray et al., 2018). Over the 5 years, the survival rate of pancreatic cancer is only 7–9% (Atay, 2020). Due to the late diagnosis, low resection rates, and elevated resistance to chemotherapeutic drugs, effective treatments for pancreatic cancer have stagnated for the past few decades despite many improvements in clinical treatment strategies for other cancers (Siegel et al., 2018). According to predictions, pancreatic cancer will rank second only to lung cancer in terms of cancer-related mortality in the future (Rahib et al., 2014).

Pancreatic adenocarcinoma (PAAD) accounts for over 90% of pancreatic cancer and remains a critical challenge in clinical process. Surgical and adjuvant chemotherapy can partially prolong long-term survival among a minority of patients (Conroy et al., 2018). The development on combination chemotherapy and radiotherapy over the past decade has provided a valuable alternative for patients having advanced or metastatic PAAD; however, nearly all patients eventually relapse, and second-line treatment options are limited (Conroy et al., 2011). Moreover, some targeted therapies, including the KRAS, CDKN2A, TP53, SMAD4, and other NRG1 or NTRK gene, also show poor outcomes in patients having advanced PAAD (Wood & Hruban, 2012; Maitra & Hruban, 2008). More importantly, emerging immunotherapy demonstrates little efficacy for PAAD patients (Jiang et al., 2017; Qian et al., 2021). PAAD is the most common kind of malignancy in terms of immune resistance. Its unusual genomic landscape, which is formed of carcinomic driving genes, induces immunosuppression and disrupts adaptive T-cell immunity from the earliest stages of tumor genesis. To date, no single-agent immunomodulators have shown clinical effectiveness; consequently, a multimodal therapy targeting the mechanism of immunotherapy resistance is still required (Bear et al., 2020).

It has recently been reported that in various cancer cells, the CKLF-like MARVEL transmembrane domain containing 6 (CMTM6) protein is associated with the controlled expression for the programed cell apoptosis 1 ligand 1 (PD-L1) protein by two critically important researches (Burr et al., 2017; Mezzadra et al., 2017). The CMTM6 protein is responsible for maintaining the persistent expression of PD-L1 and preventing its degradation by ubiquitination. The CMTM6 protein also appears to increase the half-life of the PD-L1 protein and to modulate anti-tumor immunity. The inhibition of CMTM6 in turn relieves the immunosuppression of T cells. Along with the deep-going development of recent research, expression of CMTM6 has been affirmatively related with PD-L1 protein and represents a poor estimated outcome in a variety of cancers (Liang et al., 2022). In tumor-induced immunosuppression, CMTM6 has become an importantly potential immune checkpoint, indicating its new possibilities for overcoming tumor immune resistance.

Previous researches have reflected the association between CMTM6 expression and the therapeutic response to PD-L1 pathway inhibition in patients with gastrointestinal cancers (Zhang et al., 2021; Ubukata et al., 2021; Tanaka et al., 2019; Peng et al., 2021). However, the relevance of CMTM6 and its relationship with PD-L1 within PAAD progression has not yet been clarified. To investigate the unidentified roles played by CMTM6 within PAAD, the Cancer Genome Atlas (TCGA) network was used to obtain genomic profiles, which contained somatic mutations as well as DNA copy numbers to clarify the status of CMTM6 within PAAD. Then, we displayed Kaplan–Meier survival assessment and a series of basic experiments among PAAD tissues as well as cell lines for investigating the malignant biological behaviors, including proliferation, migration, and invasion. Furthermore, we performed an integrative and in-depth analysis to demonstrate the immunological roles of CMTM6 within the tumor microenvironment (TME) in PAAD, including immune pathways, immunomodulators, immune infiltrating cells, inflammatory activities, and immunotherapy response prediction. We then validated the association between CMTM6 and PD-L1 protein by consistent Western blot and co-immunoprecipitation within PAAD cell line. We aim to identify CMTM6 as a potential immunotherapeutic target for PAAD based on theoretical implications, and this study is the first to describe CMTM6’s participation within PAAD, both clinically and molecularly.

## Materials and methods

### Information source as well as preprocessing

Gene expression atlas with clinical information from PAAD projects (including 4 normal and 179 tumor tissues) were acquired from TCGA (https://portal.gdc.cancer.gov/projects/TCGA-PAAD). We also used 167 normal pancreatic tissues for the program of Genotype-Tissue Expression (GTEx) (https://www.gtexportal.org/home/index.html) (https://gtexportal.org/home) as supplemental information for the normal tissues. Log2 transformation was applied to all RNA-seq data. The somatic mutation information was analyzed with VarScan2. Its copy number variation (CNV) information was dealt with the GISTIC method, both of which were obtained online from the TCGA-PAAD project. The Cancer Cell Line Encyclopedia (CCLE) datasets were obtained from the Broad Institute CCLE website (http://www.broadinstitute.org/ccle). Gene expression information of GSE85916, GSE78229, GSE62452, GSE57495, GSE28735, and GSE102238 datasets from the GEO database (https://www.ncbi.nlm.nih.gov) was also downloaded for validation.

### CMTM6 differential expression assessment and survival analysis

To calculate the differential expression of CMTM6, box plots and scatter plots were generated via the disease state (tumor or normal) as the variable. A curve of receiver operating characteristic (ROC) was employed for estimating the forecast performance for CMTM6. Survival assessment of Kaplan–Meier was conducted on its overall survival (OS), disease-specific survival (DSS), and progression-free interval (PFI) outcomes, and survival curves of Kaplan–Meier were also generated with other sub-cohorts of clinical features (age, T stage, histological grade, lymph node status, smoking status, history of diabetes, history of chronic pancreatitis, and alcohol history).

### Assessment involving differential expression for CMTM6 in cohorts of PAAD

Gene set enrichment assessment (GSEA) was conducted via JAVA program gsea-3.0.jar. And TCGA-PAAD tumor information was divided to CMTM6 high-expression as well as low-expression cohorts. Differentiated expressed genes were enriched by the Kyoto Encyclopedia of Genes and Genomes (KEGG) pathway, WikiPathways, and Gene Ontology (GO) gene sets. The enriched results were displayed in a bubble chart. R software with the “maftools” and “ggplot2” packages was employed for CMTM6 high- and low-expression cohorts in the somatic mutation information visualization.

### Immune-related modulators, immune cell infiltration, and inflammatory activity analysis

We examined the association involving CMTM6 as well as its immunological features of TME regarding the following aspects to confirm its function to modulate cancer immunity in PAAD. TCGA-PAAD, GSE85916, GSE78229, and GSE62452 datasets were applied to determine the association between CMTM6 and immunological activity. A total of 122 immunomodulators were identified, including receptors, chemokines, immune stimulators, and immune inhibitors. Moreover, major histocompatibility complex (MHC) from the research of Charoentong was downloaded (Charoentong et al., 2013). In these datasets, correlations involving CMTM6 and the five types of immunomodulators were computed via the Mantel test (Guillot, 2013) and the Pearson correlation coefficient. To provide graphical representations of correlations and their combinations, the “ggcor” R package v0.9.8.1 (https://github.com/houyunhuang/ggcor) was used, which was based on the “ggplot2” R package. This study was conducted via the R package GSVA to assess the enrichment of gene sets within individual samples (ssGSEA) to quantify the degrees of 24 types of immune- and tumor-related signatures in each sample. Spearman correlation was used in order to determine the relationship between CMTM6 expression with immune cell infiltration enrichment and immunomodulators as defined in (Bindea et al., 2013). Heatmaps were employed to view how the effector genes of immunity cells were expressed within CMTM6 high- and low-expression cohorts. We selected 7 clusters from 104 genes that were subsequently described to be metagenes (Wang et al., 2016; Chen & Mellman, 2013). Positive and negative correlations within the corrgram were represented by red and blue, respectively.

### Prediction of immunotherapy response

The gene expression profiles of TCGA, GSE85916, and GSE57495 were sorted out and divided into CMTM6_high and CMTM6_low groups according to the expression of CMTM6. Submap algorithm (Roh et al., 2017) was used to predict the response probability of CMTM6_high and CMTM6_low groups to immunotherapy. The predictions were based on data including the expression profiles of CTLA-4 and PD-1 following immunotherapy.

### Immunohistochemistry (IHC) staining of pancreatic cancer microarray

The PADD microarray was obtained from Shanghai Outdo Biotech Co., Ltd (HPanA060CS04), which included 52 tumor tissues and 9 para-cancerous tissues. A detailed clinical report was prepared by Shanghai National Engineering Center for Biochip. We placed the tissue chip in a 67 °C oven to remove the paraffin and hydrate it, then put it into sodium citrate buffer solution, pretreated the slice by microwave heating to extract the antigen, and covered it with a normal goat serum solution. Then, incubate the antibody against rabbit CMTM6 (Sigma-Aldrich, USA, HPA026980) and slice overnight at 4 °C. Incubation of the biotinylated goat anti-rabbit IgG secondary antibody with the slice at ambient temperature for 1 to 2 h was conducted on the second day. At last, the sections were stained with avidin and biotin peroxidase complex (GeneTex, USA). Images were captured via Olympus microscope (Japan). *P* (percentage of positive cells) value was calculated based on the percentage of malignant cells that stained positively (0, negative; 1, < 25%; 2, 26–50%; 3, 51–75%; 4, 76–100%); *I* (intensity score) value was determined by the staining intensity: strong brown = 3, medium yellow brown = 2, weak yellow = 1, and negative blue = 0. We multiply the *I* by the *P* to determine the final IHC score. IHC score > 4 was defined as positive expression of CMTM6, and scores ≤ 4 were considered as low expression of CMTM6.

### Culture of cells

There were five types of cell lines, including AsPC-1, CFPAC-1, MIA PaCa-2, PANC-1, and SW1990 cells, which were collected from Cell Bank of Type Culture Collection of the Chinese Academy of Sciences located in Shanghai. Another two cell lines, Capan-1 and Capan-2 cells, were obtained from American Type Culture Collection (ATCC). Roswell Park Memorial Institute (RPMI) 1640 medium (Thermo Fisher, USA) containing 10% fetal bovine serum (FBS) (Gibco, Australia) was employed for AsPC-1 cell culture. Dulbecco’s Modified Eagle Medium (DMEM, Thermo Fisher, USA) containing 10% FBS was employed for MIA PaCa-2, PANC-1, SW1990, and Capan-2 cell culture. CFPAC-1 and Capan-1 cells were cultured with Iscove’s Modified Dulbecco’s Medium (IMDM) containing 20% FBS (IMDM, Gibco, Australia). Cell culture was maintained under standard cell culture conditions in humidified 5% CO_2_ that had 100 mg/mL streptomycin as well as 100 U/mL penicillin.

### Extraction of RNA as well as quantitative reverse transcription polymerase chain reaction (qRT-PCR)

A TRIzol reagent (Thermo Fisher, USA) was used to extract total RNA from cells. PrimeScript reverse transcriptase (Takara, Japan) was applied to reverse transcribe RNA into complementary DNA. Glyceraldehyde 3-phosphate dehydrogenase (GAPDH) was identified as a reference gene for determining the expression degrees of the genes. Sequences of the primers were as follows: CMTM6: F: 5-TTTCCACACATGACAGGACTTC-3, R: 5-GGCTTCAGCCCTAGTGGTAT-3; as well as GAPDH: F: 5-GAAGGTGAAGGTCGGAGTC-3, R: 5-GAGATGGTGATGGGATTTC-3.

### Western blot assessment

All cell protein was extracted and lysed via radioimmunoprecipitation test (RIPA) buffer containing phenylmethanesulfonyl fluoride (PMSF) with proteinase and phosphatase inhibitor (PI, PPI). Total protein concentrations of supernatants were measured based on the instructions provided by the manufacturer via a test kit for Pierce BCA Protein (Thermo Fisher, USA). We subjected the protein samples to sodium dodecyl sulfate-polyacrylamide gel electrophoresis (SDS-PAGE) and then transferred them to a polyvinylidene difluoride (PVDF) membrane for further assessment (Millipore, Billerica, MA, USA). We blocked the membranes with 5% nonfat milk in Tris-buffered saline with 0.1% Tween-20 (TBST) for 2 h at ambient temperature and incubated them with appropriate concentrations of primary antibodies for overnight incubation at 4 °C (CMTM6, Sigma-Aldrich, USA, HPA026980; GAPDH, Proteinch, China, 10494-1-AP; PD-L1, Cell Signaling Technology, USA, 13684). For 1 h at ambient temperature, secondary antibodies conjugated to horseradish peroxidase were incubated with the membranes (M21008, Abmart) after three washes in TBST for 10 min each. A chemiluminescence system was used to visualize and capture immunoreactive protein bands.

### Lentiviral construction and transduction

In order to construct stable cell lines that express inhibition of CMTM6 expression, we transduced Capan-2 cells with lentivirus (at a multiplicity of infection of 10) containing either control shRNA or CMTM6-specific shRNA (Sangon Biotech, Shanghai, China) and cultured the cells in puromycin (5 g/mL) for 4 days. As a measure of knockdown efficiency, Western blotting was performed. Its sequences of CMTM6-specific shRNA were shRNA#1 5′-CCCAAGACAGTGAAAGTAATT-3′, shRNA#2 5′-TGGAGAACGGAGCGGTGTACA-3′.

### Cell Counting Kit-8 (CCK-8) assay

We plated Capan-2 cells (5000/well) to 96-well plates. After attaching tightly, cell viability was ascertained at 0, 1, 2, 3, and 4 days with 10 μL of CCK-8 reagent (Dojindo, Japan) per well. Absorbance at 480 nm was measured via a microplate reader. Growth curves were plotted via average absorption values.

### Colony formation test

In 6-well plates, cells were plated and permitted to continuously grow for 12 to 14 days. Four percent paraformaldehyde with 0.1% crystal violet was then employed to fix all colonies for 30 min prior to staining. We cleaned the plates three times with phosphate-buffered saline (PBS), then scanned the images, and counted the colonies visually.

### Wound-healing test and cell invasion test

Approximately 90% of the Capan-2 cells were plated in 24-well plates. After attachment of the cells, artificial wounds were created by scraping the monolayer of the cells with a 200-μL sterile pipette tip. We photographed the wound area at 0 h and 24 h in order to calculate the percentage of wound closure. Cell invasion assays were conducted in 24-well cell culture chambers by using inserts containing 8-μm pore membranes pre-coated with Matrigel. In the upper wells, the test cells were placed without serum, and the lower wells were filled with conditioned medium. Following 24 h of fixation with 0.5% glutaraldehyde, the cells were stained with 0.5% toluidine blue. Three fields of view/membranes were examined under a light microscope (×20), and its number of invading cells was normalized to the total amount of cells.

### Co-immunoprecipitation (Co-IP)

Capan-2 cells were lysed via applying RIPA lysis buffer that contains 1 to 100 PI and PPI. Then, cell protein was treated with anti-CMTM6 antibodies over 4 h around 4 °C anti-PD-L1 antibody (CMTM6, Sigma-Aldrich, USA, HPA026980; PD-L1, Cell Signaling Technology, USA, 13684), or immunoglobulin IgG (2 μg) as a negative control. Cells were slowly mixed overnight at 4 °C after adding 20 μL Dynabeads (sc-2003, Santa Cruz Biotechnology, Dallas, TX). The cells were cleaned 3 times with ice-cold PBS to eliminate the beads as well as unbound antibodies, re-suspended in 1× SDS loading buffer for 5 min at 95 °C, separated by SDS-PAGE, and immunoblotted.

### Statistical analysis

Pearson or Spearman coefficients were applied to assess its correlations involving continuous variables. The correlation between clinical features of PAAD patients as well as CMTM6 protein expression was evaluated via the test of Wilcoxon rank sum. The Student *t*-test and one-way ANOVA were employed to evaluate variations in the parameters involving different cohorts. It was determined that CMTM6 had a prognostic effect on survival via the Kaplan–Meier approach, as well as log-rank testing to compare the survival curves. In univariate as well as multivariate Cox proportional hazard assessment, CMTM6 was assessed for its prognostic value. A *P*-score of below 0.05 was deemed significant from a statistical perspective. IBM SPSS statistical software was used for all statistical analyses (v26, Armonk, NY; IBM Company). The R programming language (v4.1.3; https://www.r-project.org/) was used to carry out the relevant data analysis.

## Results

### CMTM6 is highly expressed within PAAD tissues as well as cell lines

**Fig. 1:**
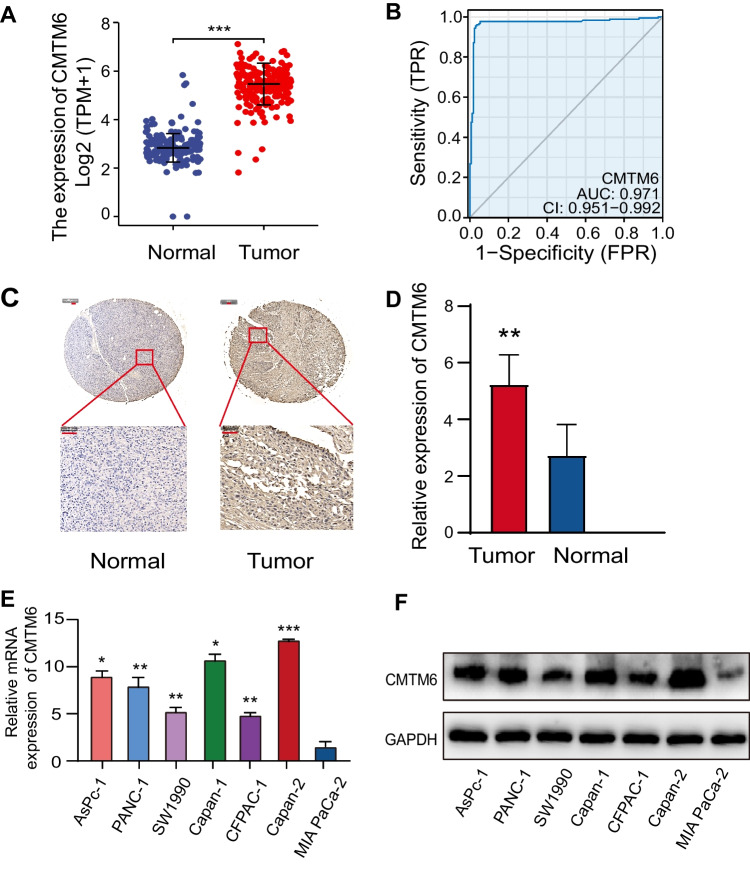
The CMTM6 expression within PAAD tissues as well as cell lines. **A** Its mRNA CMTM6 expression involving PAAD tissues and non-cancerous pancreatic tissues in TCGA and GTEx databases. **B** Receiver operating characteristic curve to confirm the CMTM6’s extremely significant expression within PAAD. **C** An immunohistochemical staining of CMTM6 within PAAD tissue and normal para-cancerous tissue. The scale bars are 50 μm in width. **D** The staining results were evaluated based on the percentage of positive cells and the intensity of staining. **E** The mRNA expression of CMTM6 within PAAD cell lines by qRT-PCR. **F** The protein level of CMTM6 within PAAD cell lines by western blotting. *P < 0.05; **P < 0.01; ***P < 0.001

Using RNA sequencing data from the TCGA and GTEx data sources, we examined the mRNA expression of CMTM6 inside PAAD. The results showed that CMTM6 was strongly expressed in 179 samples with PAAD than 171 samples of normal tissues (*P* < 0.001) (Fig. 1A). The differentiating effectiveness of CMTM6 within PAAD and normal pancreatic tissues was also examined by using the ROC curve. Surprisingly, the area under the curve (AUC) for the expression of CMTM6 reached 0.971 (Fig. 1B). This suggests that CMTM6 is highly expressed within PAAD and could be a molecular biomarker with potential for patients having PAAD. We then detected the protein expression of CMTM6 within PAAD microarray. Typical PAAD tissues and normal pancreatic tissues were displayed in Fig. 1C. The IHC outcomes also indicated that the expression of CMTM6 within PAAD tissues was greater than that within pancreatic tissues in normal state (*P* < 0.01) (Fig. 1D). Furthermore, to further investigate the expression of CMTM6 in various pancreatic cancer cell lines, the cell line expression matrix of pancreatic cancer was obtained from the CCLE dataset, and we analyzed it with the software package ggplot2 for R v4.0.3 (v3.3.3). The distribution of expression of CMTM6 in different cell lines was indicated in Supplement Fig. S1. Then, using a range of pancreatic cancer cell lines, including AsPc-1, PANC-1, SW1990, Capan-1, CFPAC, Capan-2, and MIAPaca-2, we examined the mRNA and protein degrees of CMTM6 by qRT-PCR and Western blot. Their expression of CMTM6 was compatible with the results of CCLE (Fig. 1E–F). Among the existing cell lines, Capan-2 cells showed the highest expression of CMTM6, followed by Capan-1, AsPc-1, and PANC-1 cells with moderate expression, and the SW1990, CFPAC-1, and MIAPaCa-2 cells showed relatively weak expression. These findings imply that CMTM6 could function as a possible biomarker for PAAD and that CMTM6 involvement in the network regulation of PAAD deserves further investigation.

### CMTM6 predicts high-grade clinical features and poor survival in patients with PAAD

**Fig. 2:**
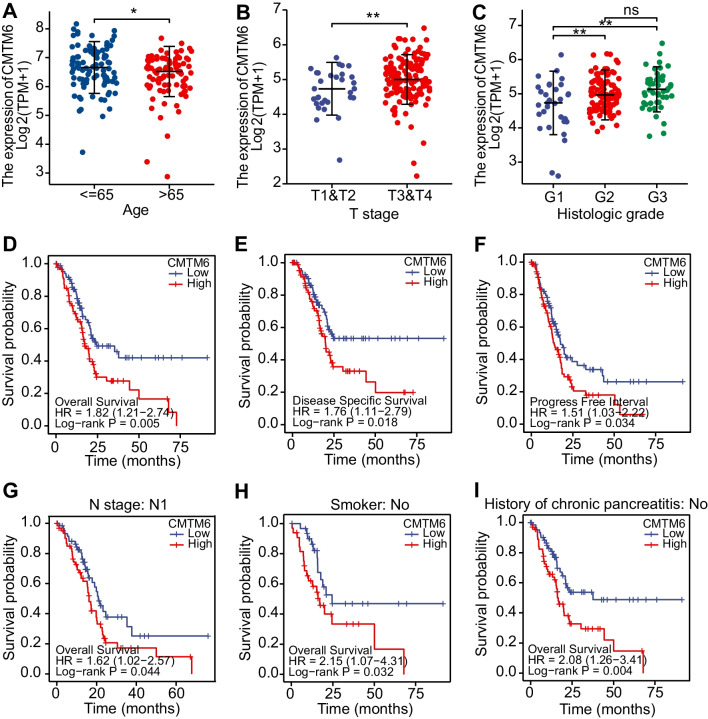
The clinical outcome as well as prognostic score for CMTM6 in patients having PAAD. **A** The expression of CMTM6 involving age ≤ 65 and age > 65 cohorts within PAAD patients. **B** The expression of CMTM6 involving T1&T2 and T3&T4 cohorts within PAAD patients. **C** The expression of CMTM6 involving G1, G2, and G3 cohorts within PAAD patients. **D**–**F** Kaplan–Meier survival curves using the log-rank test for OS, DSS, and PFI for all PAAD patients. **G**–**I** Survival curves of Kaplan–Meier for OS of N1, non-smoker, and no history of chronic pancreatitis sub-cohorts within PAAD patients by log-rank test. OS, overall survival; DSS, disease-specific survival; PFI, progress free interval. *P < 0.05; **P < 0.01; ***P < 0.001

It was proved that the CMTM6 expression within PAAD was greatly higher through the above analysis, which prompted us to further investigate the impact of the elevated CMTM6 expression on the clinical features as well as survival time for PAAD patients. Because of the histopathological heterogeneity of pancreatic cancer, we analyzed the RNA sequence information of PAAD based on the WHO grading system, histology, and age classification. The expression of CMTM6 within PAAD patients younger than 60 years was higher than that within PAAD patients older than 60 years (Fig. 2A). In comparison to T1 and T2 stage, CMTM6 had the highest expression in T3 and T4 stage of PAAD (Fig. 2B). In addition, the expression of CMTM6 increased in higher histopathological grade (G3 > G2 > G1) malignancies (Fig. 2C). Furthermore, to determine the prognostic score of CMTM6, we performed a Kaplan–Meier analysis within PAAD from a clinical perspective. The median OS time was longer in the CMTM6 low cohort than in the CMTM6-elevated cohort (hazard ratio (HR) = 1.82; *P* = 0.005; Fig. 2D). The rates of DSS were especially higher in patients having reduced expression of CMTM6 than among those with elevated expression of CMTM6 (HR = 1.76; *P* = 0.018; Fig. 2E). Similarly, the PFI rates in the CMTM6-elevated cohort were greatly lower than those in the CMTM6 low cohort (HR = 1.51; *P* = 0.034; Fig. 2F). Then, we carried out sub-cohort survival assessments of OS, which indicated that the estimated outcome of patients having CMTM6 elevated was weak in N1 (HR = 1.62; *P* = 0.044), non-smokers (HR = 2.15; *P* = 0.032), and no history of chronic pancreatitis sub-cohort (HR = 2.08; *P* = 0.004; Fig. 2G–I). The sub-cohort survival assessments of DSS indicated that the predicted survival time of patients having CMTM6 elevated was shorter in no history of chronic pancreatitis sub-cohort (HR = 1.88; *P* = 0.025; Supplement Fig. S2A) and diabetes history sub-cohort (HR = 9.33; *P* = 0.009; Supplement Fig. S2B). And the sub-cohort survival assessments of PFI demonstrated that the predicted survival outcome of CMTM6 high-expressed patients was worse in G1 grade (HR = 4.29; *P* = 0.012; Supplement Fig. S2C), no alcohol history (HR = 2.09; *P* = 0.027; Supplement Fig. S2D), and non-smokers sub-cohort (HR = 2.49; *P* = 0.004; Supplement Fig. S2E). Additionally, we conducted a Cox regression assessment to reveal the importance of CMTM6 for clinical prognosis within PAAD. The univariate assessment showed that CMTM6, T stage, N stage, histologic stage, and history of chronic pancreatic pancreatitis were greatly related to OS within TCGA-PAAD (Table 1). In the multivariate analysis, CMTM6 remained a significant predictor even after adjusting for T stage, N stage, and histologic stage. These results suggest that CMTM6 could be an indicator of poor estimated outcomes in patients with PAAD.

**Table 1:** The expression of CMTM6 in univariate and multivariate cox regression analyses in TCGA-PAAD.

		Univariate analysis		Multivariate analysis	
Characteristics	Total (*N*)	Hazard ratio (95% CI)	*P* value	Hazard ratio (95% CI)	*P* value
T stage	176				
T1 and T2	31				
T3 and T4	145	2.023 (1.072–3.816)	**0.030**	1.308 (0.657–2.603)	0.445
N stage	173				
N0	50				
N1	123	2.154 (1.282–3.618)	**0.004**	2.055 (1.177–3.586)	**0.011**
Pathologic stage	175				
Stage I and Stage II	167				
Stage III and Stage IV	8	0.673 (0.212–2.135)	0.501		
Histologic grade	176				
G1 and G2	126				
G4 and G3	50	1.538 (0.996–2.376)	**0.042**	1.218 (0.781–1.901)	0.385
Smoker	144				
No	65				
Yes	79	1.086 (0.687–1.719)	0.724		
Alcohol history	166				
No	65				
Yes	101	1.147 (0.738–1.783)	0.542		
History of diabetes	146				
No	108				
Yes	38	0.927 (0.532–1.615)	0.790		
History of chronic pancreatitis	141				
No	128				
Yes	13	1.177 (0.562–2.464)	**0.046**		
Family history of cancer	110				
No	47				
Yes	63	1.117 (0.650–1.920)	0.689		
Age	178				
≤ 65	93				
> 65	85	1.290 (0.854–1.948)	0.227		
CMTM6	178				
Low	89				
High	89	1.828 (1.198–2.788)	**0.005**	1.549 (1.006–2.386)	**0.047**

The *P* value < 0.050 were in bold emphasis

### Expression of CMTM6 is related with different patterns of genomic mutation

**Fig. 3:**
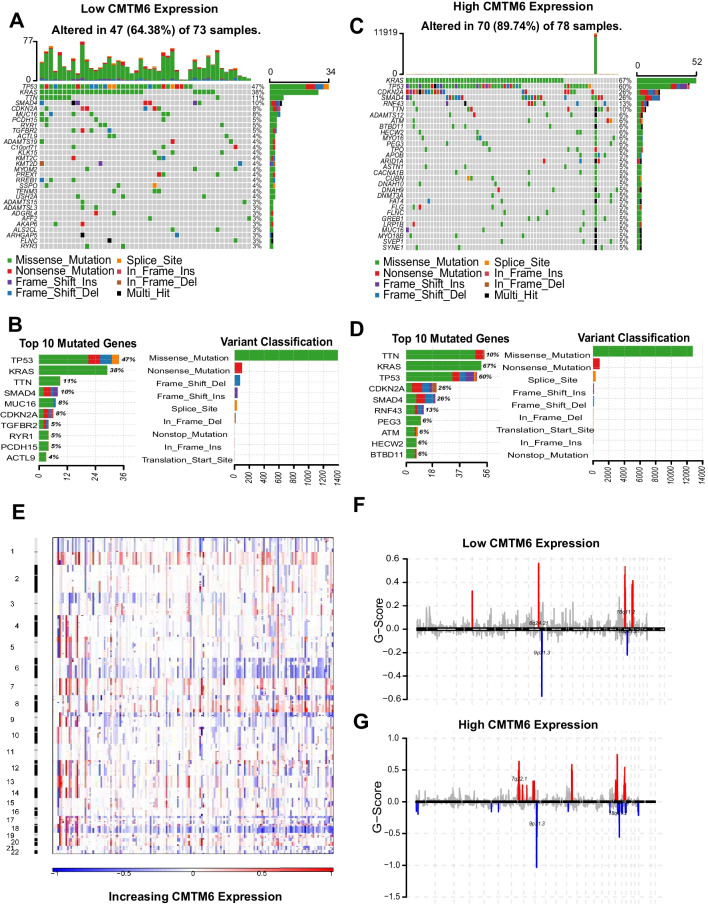
Distinctive genomic mutation profiles related with expression of CMTM6. **A** Somatic mutation alterations in reduced expression of CMTM6 cohort. **B** Top 10 mutated genes and variant classification in reduced expression of CMTM6 cohort. **C** Somatic mutation alterations in elevated expression of CMTM6 cohort. **D** Top 10 mutated genes and variant classification in elevated expression of CMTM6 cohort. **E** A summary of the overall CNA profile in order of increasing expression of CMTM6. **F**–**G** Amplifications and deletions of GISTIC 2.0 within PAAD with reduced and elevated expression of CMTM6. On the chromosomal map, significant recurring focal amplifications (red) and deletions (blue) are illustrated

In order to investigate the cellular mechanism of CMTM6 mediated in PAAD, we analyzed somatic mutation and copy amount alternations in TCGA database. We compared the frequency of somatic mutations between patient groups with elevated and reduced expression of CMTM6. The somatic mutations were more prevalent in patients with elevated expression of CMTM6 (70 of 78, 89.74%), and only 47 of 73 (64.38%) (Fig. 3C; Supplement Fig. S3B) somatic mutation alternations were revealed among patients having decreased expression of CMTM6 (Fig. 3A; Supplement Fig. S3A). Then, we showed the difference of top 10 mutated genes, variant classification, single nucleotide variant (SNV) class, and variant type within Fig. 3B, D between CMTM6 high and CMTM6 low groups. Among these mutations, elevated frequency of mutations in TP53, KRAS, TTN, SMAD4, and MUC16 was observed in samples with reduced expression of CMTM6. Besides, elevated frequency of mutations in CDKN2A, RNF43, PEG3, ATM, and HECW2 was also noticed in samples having elevated expression of CMTM6. And both in the elevated or reduced expression of CMTM6 cohorts, missense mutation type, C > T class, and single nucleotide polymorphisms (SNP) type were always at the top. Next, we further investigated the CNAs between CMTM6-reduced expression and CMTM6-elevated expression cases. Figure 3E showed the general CNA profile in order of increasing expression of CMTM6. The results demonstrated that an amplification of Chr 7 and 8 along with a deletion of Chr 6 was accompanied with elevated expression of CMTM6 of PAAD. Based on the GISTIC 2.0 assessment of all PAAD samples, significant recurrences of focal amplifications and deletions were identified. 8q24.21 and 18q11.2 exhibited significant amplifications, whereas 18q21.2 was frequently deleted in the reduced expression of the CMTM6 cohort (Fig. 3F). Peaks in 7q22.1 and 18q21.2 and deletions in 18q11.2 were found in elevated expression of CMTM6 cohort (Fig. 3G). Moreover, we detected 9q21.3 (CDKN2A and CDKN2B) deletion in all cases with elevated and reduced expression of CMTM6, but the G score of elevated expression cohort was greatly greater in elevated cohort than that of reduced CMTM6 expression cohort within PAAD (Fig. 3F, G).

### CMTM6 is engaged in immunological as well as inflammatory biological processes

**Fig. 4:**
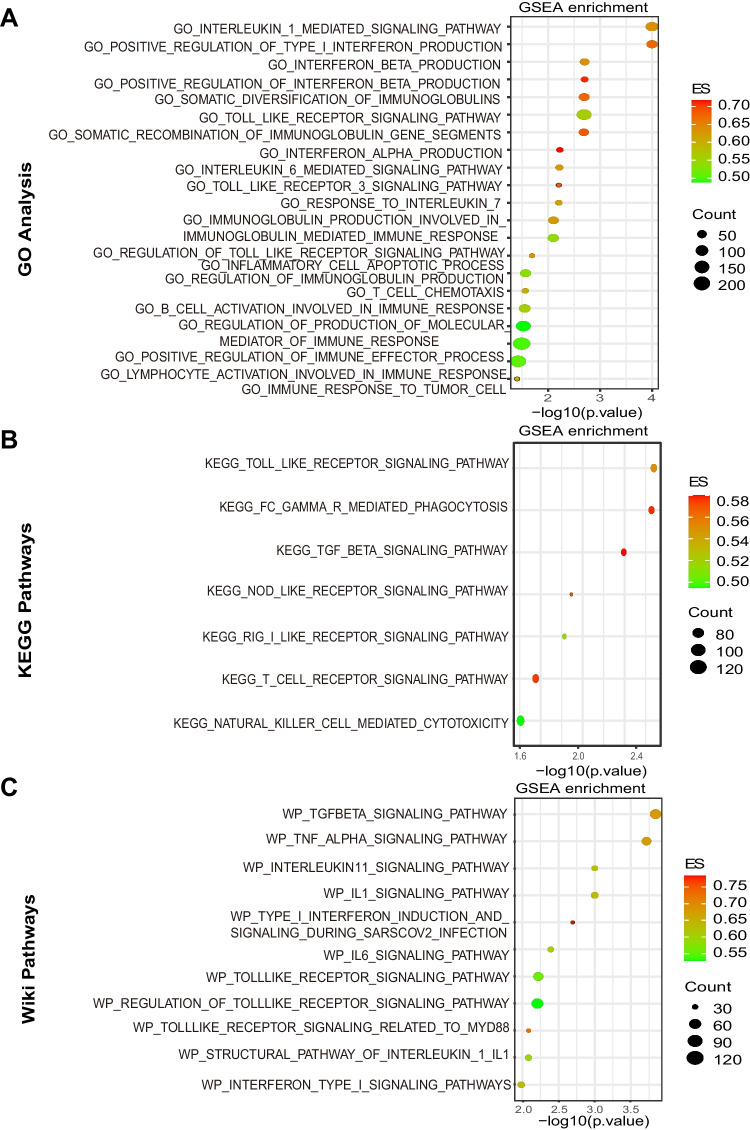
Expression of CMTM6 enriched in immunological and inflammatory biological functions. **A** Immune- and inflammation-related signaling pathways enriched in positive expression of CMTM6 within PAAD by GO analysis. **B** Immune- and inflammation-related signaling pathways enriched in positive expression of CMTM6 within PAAD by KEGG analysis. **C** Immune- and inflammation-related signaling pathways enriched in positive expression of CMTM6 within PAAD by Wiki analysis. GO, Gene Ontology; KEGG, Kyoto Encyclopedia of Genes and Genomes; ES, enrichment score

Based on the results mentioned above, CMTM6 contributes to critical PAAD biological functioning in PAAD progress. Previous researches have reported that CMTM6 promotes tumor progression via modulation of immunity of T cells in a variety of tumors. To thoroughly investigate the immune-related pathway changes and the association involving expression of CMTM6 and immune signaling pathway within PAAD, GSEA enrichment assessment was performed to reveal the potential significance (adjusted *P* < 0.05). The biological functions for the related genes were conducted via GO, KEGG, and WikiPathways analyses. We found that expression of CMTM6 was increasingly associated with most immunity responses and inflammatory activities. Based on their normalized enrichment score (NES) ratings, the most highly enriched immune-related signaling pathways were chosen. GO assessment indicated that expression of CMTM6 was enriched in the immune response to tumor cells, interleukin-1-mediated signaling pathway, inflammatory cell apoptotic process, and T-cell chemotaxis (Fig. 4A). KEGG assessment revealed that expression of CMTM6 was enriched in T-cell receptor, cytotoxicity caused by natural killer (NK) cells as well as Toll-like receptor (TLR), and Fc gamma R-mediated phagocytosis signaling pathways (Fig. 4B). WikiPathways assessment demonstrated that expression of CMTM6 was enriched in the IL1, IL6, interleukin 11, and Toll-like receptor signaling pathways (Fig. 4C). As a result of these findings, it strongly demonstrates that CMTM6 contributes to both immune function and inflammatory response within PAAD.

### Expression of CMTM6 is related with immunomodulators within PAAD

**Fig. 5:**
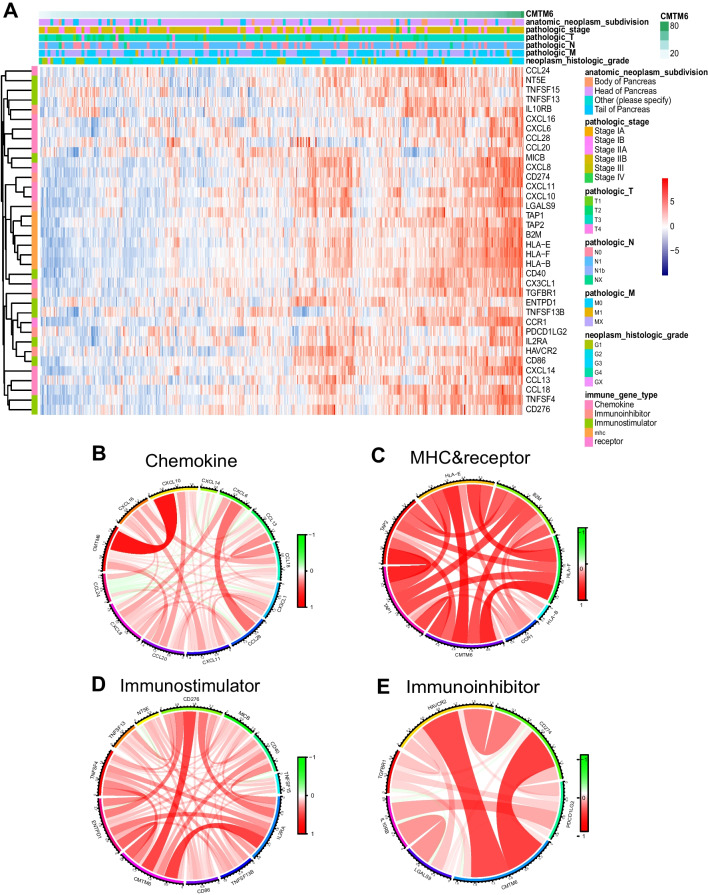
The association involving expression of CMTM6 and immunomodulators in TCGA-PAAD. **A** Immunomodulators were mostly affirmatively related with expression of CMTM6 in TCGA-PAAD as indicated in heatmap. **B**–**E** Correlation of significant immunomodulators with expression of CMTM6 in TCGA-PAAD, including four modules: chemokine, MHC&receptor, immunostimulatory, and immunoinhibitory

To learn more about how CMTM6 could function immunologically inside PAAD, we first prepared 122 immunomodulators from Charoentong’s research (Supplement Table S1) (Chen & Mellman, 2013), including chemokines, MHC, immunological stimulators, immunity inhibitors, and receptors. We screened 36 immunomodulators which were positively related with expression of CMTM6 (*P* < 0.05; Supplement Table S2; Supplement File 1). As indicated in the heatmap, CMTM6 was positively associated with these immunomodulators by integrating the specific clinical characteristics, including anatomic neoplasm subdivision, pathologic TNM, pathological stage, and histologic grade (Fig. 5A). To investigate the further association between CMTM6 expression and these significant immunomodulators, we have carried out a circle graph assessment (Fig. 5B–E). Three important immunomodulators (CXCL10, CXCL11, and CCR1) were affirmatively associated with expression of CMTM6 within PAAD in a significant manner, which played an important role in inducing the CD8+ T-cell recruitment to the TME. Other chemokines and receptors, such as CCL13, CCL18, CCL20, CCL28, CD40, CD88, CXCL6, and CXCL14, enhancing Th2 cells and CD8^+^ T-cell recruitment, were also positively associated with CMTM6 expression. Importantly, CD274 (PD-L1) as a critical immune checkpoint was found to be critically related with expression of CMTM6 within PAAD. CMTM6 has been revealed to be an important regulator of PD-L1 that is involved in immune surveillance in tumor immunity escape in current research. These important relationships between CMTM6 and the manifold immunomodulator system extremely clarify the immunological effect of CMTM6 within PAAD.

### Expression of CMTM6 is related with immunity cell infiltration within PAAD

**Fig. 6:**
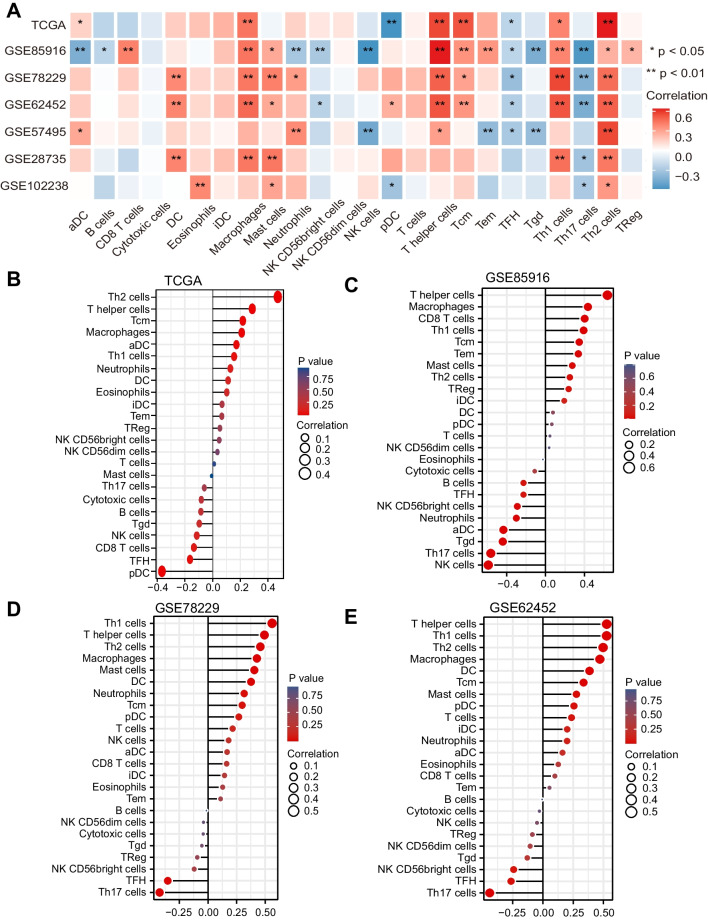
The association involving expression of CMTM6 and immune cell infiltration in TCGA and GEO datasets. **A** The correlation between 24 immunity cell infiltration fractions and the expression of CMTM6 in TCGA-PAAD and six GEO datasets. **B** Lollipop graph showed the connection involving expression of CMTM6 as well as 24 immunity cell infiltration fractions within TCGA-PAAD. **C**–**D** In GSE85916, GSE78229, and GSE62452, a lollipop graph demonstrated a connection between the expression of CMTM6 and 24 immunity cell infiltration fractions. *P < 0.05; **P < 0.01; ***P < 0.001

**Fig. 7:**
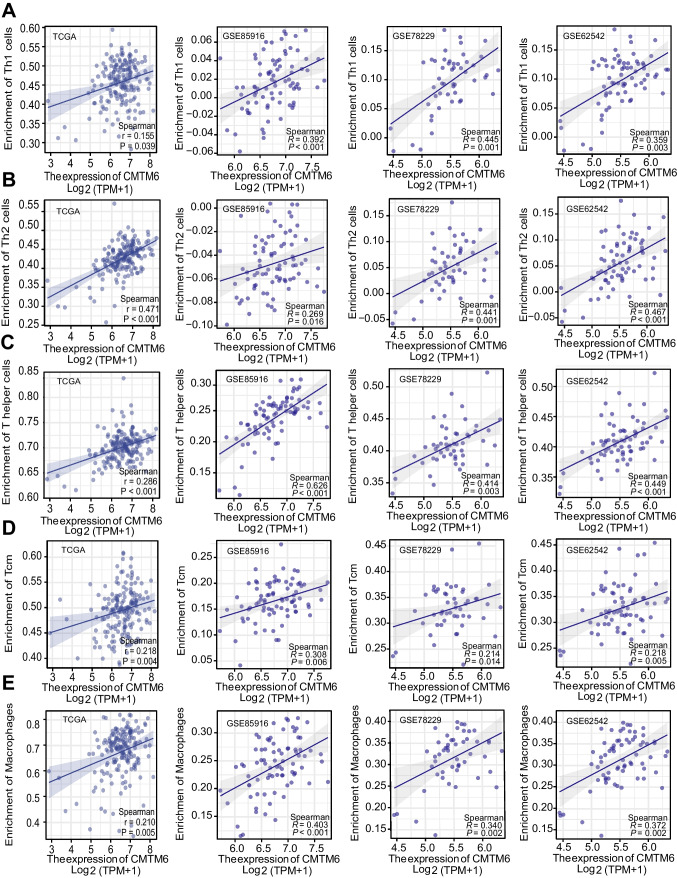
The relationship between five typical immune cell infiltration and CMTM6 expression in TCGA and GEO database. **A**–**E** Scatter plot displays the positive association involving expression of CMTM6 and enrichment of Th1, Th2, T helper, Tcm, and macrophage in TCGA-PAAD, GSE85916, GSE78229, and GSE62542

**Fig. 8:**
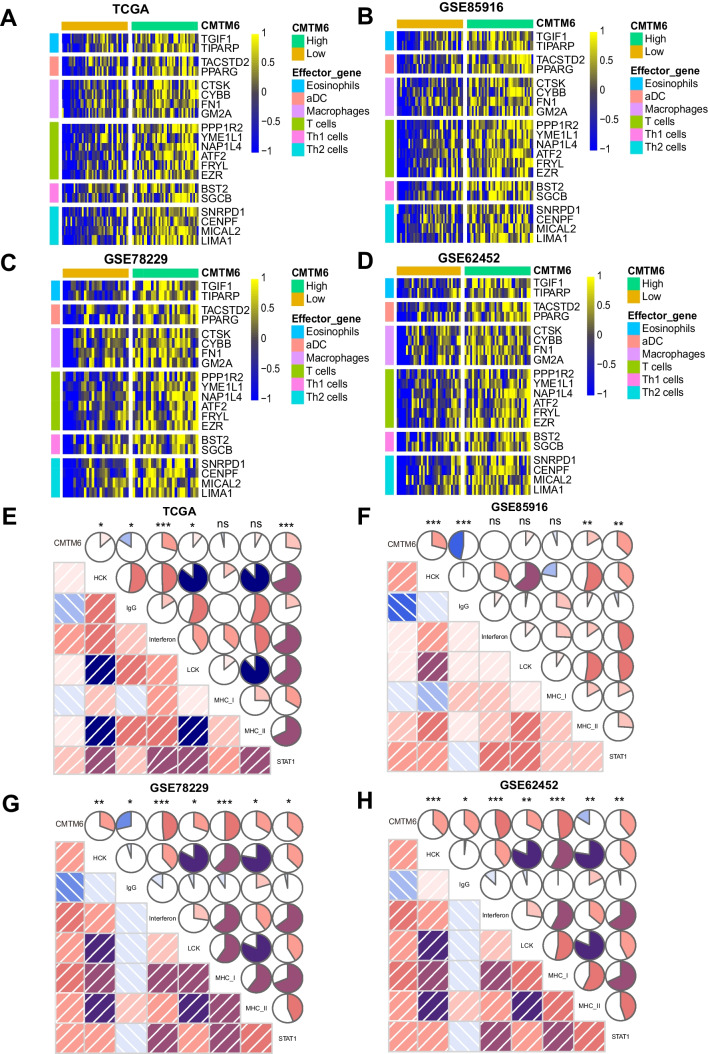
CMTM6 is affirmatively related with expression of effector genes for immunity cells and inflammatory activities in TCGA and GEO database. **A**–**D** Heatmaps show the expression of effector genes of immunity cells (eosinophils, aDC, macrophages, T cells, Th1 cells, and Th2 cells) involving low and high CMTM6-expressed cohort in TCGA-PAAD, GSE85916, GSE78229, and GSE62452. **E**–**H** Correlations involving expression of CMTM6 and inflammatory activities in TCGA-PAAD, GSE85916, GSE78229, and GSE62452. Clockwise represents positive correlation, and counterclockwise represents negative correlation. The area of the circle represents Pearson’s correlation coefficients (*R*) (0–1). *P < 0.05; **P < 0.01; ***P < 0.001

We used the ssGSEA method to calculate the composition of immunity cells in 179 TCGA-PAAD samples and quantify the heterogeneity of 24 sub-populations of immunity cells in the mixed cell population. The lollipop chart displayed the relationship between CMTM6 expression and immune cell infiltration fraction (Fig. 6B, Supplement Table S3). To further verify the infiltration of these immune cells, we chosen six PAAD datasets from GEO database (GSE85916, GSE78229, GSE62452, GSE57495, GSE28735, and GSE102238) for the same ssGSEA assessment (Fig. 6A). The outcomes were consistent with those of TCGA in three typical datasets including GSE85916, GSE78229, and GSE62452 (Fig. 6C–E, Supplement Table S4, S5, S6). Among these cells, the enrichment of Th1 cells (Fig. 7A), Th2 cells (Fig. 7B), T helper cells (Fig. 7C), Tcm cells (Fig. 7D), and macrophages (Fig. 7E) was affirmatively associated with expression of CMTM6 in TCGA and three typical GEO datasets. In contrast, the increased enrichment of TFH cells (Supplement Fig. S4A) and pDC cells (Supplement Fig. S4B) was accompanied with the decreased expression of CMTM6 in TCGA-PAAD. As we expected, the upregulated expression of CMTM6 indicated the increased expression of corresponding effector genes of these immunity cells, including Th1 cells, Th2 cells, T cells, aDC cells, and macrophages, which was consistent with our above immune cell infiltration assessment results (Fig. 8A–D). Surprisingly, the effector gene expression of eosinophils was also increasingly related with expression of CMTM6. The above outcomes further verify that CMTM6 especially contributes to regulating tumor immunity within PAAD.

### Expression of CMTM6 is related with inflammatory activities within PAAD

GSEA enrichment pathway assessment revealed that CMTM6 participated in the inflammatory response within PAAD progression. Therefore, we employed the approach previously described to calculate the expression degrees of 7 metagenes to reflect the function of CMTM6 in the inflammatory reaction (Hidalgo, 2010). As indicated in Fig. 8E, expression of CMTM6 was positively related with inflammatory activities, including HCK, interferon, LCK, and STAT1 in TCGA-PAAD. And in Fig. 8F, expression of CMTM6 was positively associated with HCK, MHC II, and STAT1 in GSE85916. More importantly, in GSE78229 and GSE62542, expression of CMTM6 was positively related with all HCK, interferon, LCK, MHCI, MHC II, and STAT1 (Fig. 8F–G). However, in both TCGA and GEO datasets, the expression of CMTM6 was related to IgG in a negative manner. These outcomes revealed that overexpression of CMTM6 promoted its upregulation for antigen presenting cells, macrophage activation, and T-cell signal transduction. Therefore, we demonstrate that CMTM6 plays crucial roles in inflammatory and immunological processes within PAAD, and expression of CMTM6 is strongly related with the progression of an inflammatory and immune-mediated TME.

### CMTM6 contributes to proliferation, migration, and invasion of PAAD cells

**Fig. 9:**
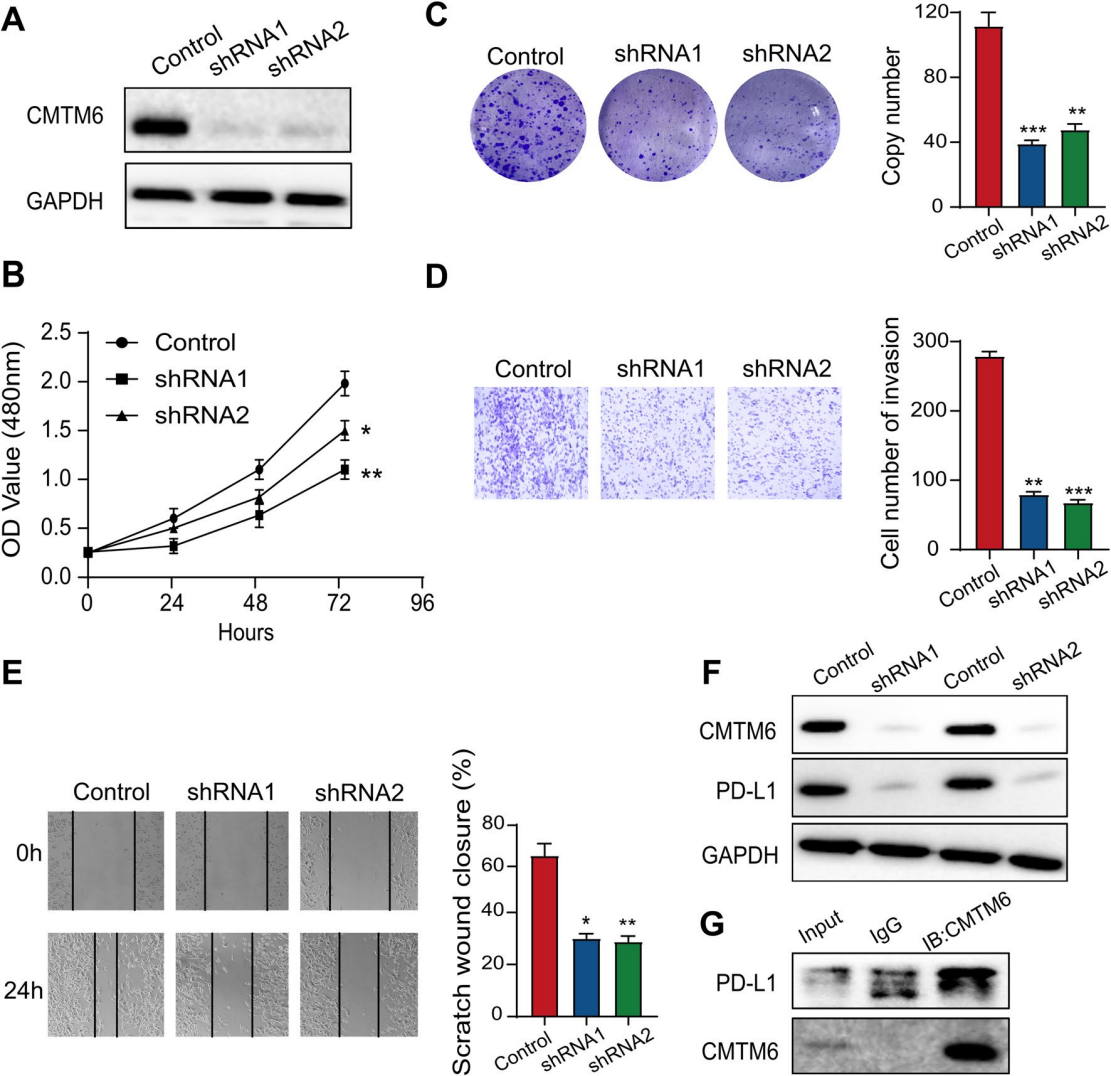
CMTM6 enhances proliferation, migration, and invasion of PAAD cells and can bind with PD-L1 protein. **A** CMTM6 protein degrees analyzed by Western blotting in Capan-2 cells. They were transduced with lentiviruses encoding either a CMTM6 shRNA or a control shRNA. **B** CCK-8 test was to determine the proliferation efficiency in Capan-2 cells. **C** Colony forming to reflect the proliferation efficiency of Capan-2 cells. **D** Transwell assays to assess cell invasion rate in Capan-2 cells. **E** Wound-healing test to examine cellular migration abilities in Capan-2 cells. **F** Western blotting to detect the protein degrees of PD-L1 after silencing expression of CMTM6 in Capan-2 cells. **G** Co-IP assessment to reveal the interaction involving CMTM6 and PD-L1 protein in Capan-2 cells. Anti-CMTM6 or anti-PD-L1 antibodies were employed to immunoprecipitate the lysates. *P < 0.05; **P < 0.01; ***P < 0.001

Considering that CMTM6 is expressed is upregulated within PAAD and its elevated expression indicates a worse estimated outcome of PAAD patients, to further research the biological function of CMTM6, we carried out a loss-of-function experiment. Firstly, we selected the Capan-2 cell with the highest expression of CMTM6. Secondly, we silenced expression of CMTM6 by transducing with lentiviruses encoding CMTM6 shRNA into Capan-2 cells (Fig. 9A). Surprisingly, via CCK-8, we found that silencing CMTM6 greatly lowered the proliferative ability of Capan-2 cells (Fig. 9B). Consistently, the cell density of CMTM6-silenced cells was much lower compared with the negative control by colony formation test (Fig. 9C). Transwell tests were displayed to assess the modification of cellular invasiveness within Capan-2 cells, and CMTM6 silencing decreased invasive ability of Capan-2 (Fig. 9D). Moreover, in a wound-healing experiment, the CMTM6 silencing cohort’s cell migratory capacity was reduced (Fig. 9E), which invaded the matrix more slowly than control cohort. Therefore, CMTM6 promotes proliferation, invasion, and migration within PAAD cells.

### CMTM6 can interact with PD-L1 in protein level, and elevated CMTM6 expression may represents better immunotherapeutic response

CMTM6 has been reported to be a critical regulating factor for PD-L1, which can have an interaction with PD-L1 and stabilize sustain expression of PD-L1 protein. Being the most representative immune checkpoint, PD-L1 can transmit immunosuppressive signals by combining with PD-1 to decrease the proliferation as well as activation of CD8+ T cells in immune systems. Combination immunotherapy, especially targeting the immune checkpoints, greatly enhances the clinical benefits in current research. In CMTM6-depleted Capan-2 cells, we subsequently examined the link between the expression of CMTM6 and PD-L1 protein. The results demonstrated that inhibition of CMTM6 greatly decreased the PD-L1 expression (Fig. 9F). To further validate the interaction involving CMTM6 and PD-L1 protein, we conducted co-IP assays. The Western blot test detected a strip, suggesting a potential bind between CMTM6 and PD-L1 (Fig. 9G). Above experiments verified that inhibition of CMTM6 reduced PD-L1 expression and that CMTM6 directly bound with PD-L1; thus, CMTM6 may affect anti-PD-1/PD-L1 or other immunotherapeutic response. We chose several immunotherapy cohorts for relevant bioinformatics analysis. The results showed that patients with elevated CMTM6 expression may receive better immunotherapeutic response in TCGA-PAAD, GSE57495, and GSE85916 cohorts (Figs. S5A-5C).

## Discussion

PAAD as one of the most malignant and lethal solid tumors, its symptoms are non-specific, and patients with PAAD often present at an advanced stage (Hidalgo, 2010). Conventional cytotoxic chemotherapy provides the benefit of only a few months of OS in patients having PAAD (Conroy et al., 2011; Von Hoff et al., 2013). This poor therapeutic efficacy has fueled the ongoing efforts to unearth the roles of the TME within PAAD therapy. These strategies include the following: (1) Targeting desmoplasia, such as matrix metalloproteinases, hyaluronan, Sonic hedgehog signaling pathway which is responsible for pancreas development, and stromal cells just like cancer-associated fibroblasts; (2) targeting a metabolic convergence to enable TME remodeling; (3) targeting the focal adhesion kinase pathways to remodel the TME; (4) disrupting TGF-β signaling in the TME; and (5) other efforts to target immune compartment in the TME (Ho et al., 2020; Sharma et al., 2020; Altman et al., 2016; Christmas et al., 2018; Mitchem et al., 2013; Jang et al., 2017; Kinkead et al., 2018; Blair et al., 2019; Hosein et al., 2019). Among these treatments, immunotherapy remains a critical new method that has been applied to some advanced metastatic cancers, specifically melanoma, renal cell carcinoma, and lung cancer (Burr et al., 2017; Mezzadra et al., 2017; Martinez-Morilla et al., 2020; Zhao et al., 2019; Tulchiner et al., 2021). However, when immunotherapy is employed for PAAD, in clinical trials, single-agent immune modulators have been found to be ineffective. As a result, multimodal therapies that targeting the mechanisms of immunotherapy resistance are required.

In this research, we demonstrate that CMTM6 is a promising immunotherapy target for PAAD treatment. We reveal that CMTM6 construct an immunological and inflammatory TME and demonstrate that CMTM6 is affirmatively associated with the immunological status of TME within PAAD by combining GEO profiles, TCGA database, and integrated bioinformatics analysis. Moreover, we verify that CMTM6 accurately predicts poor estimated outcome and represents malignant oncological function within PAAD through experimental verification. These outcomes illustrate the significance of CMTM6 as a potential immunotherapy target for PAAD.

Pancreatic cancer has elevated malignancy and poor estimated outcome, mainly due to elevated invasion, late diagnosis, and low resection rates (Ansari et al., 2016). In recent years, although there have been a few new treatment options available, such as the olaparib, a poly ADP-ribose polymerase inhibitor, and larotrectinib, an inhibitor of BRCA1 and BRCA2 mutations, as well as entrectinib for neurotrophic tyrosine kinase fusion tumors, more potential targets and clinical trials are urgently required to investigate improvement in terms of therapeutic effect within PAAD (Sohal et al., 2020; Li et al., 2005; Roth et al., 2001). CMTM6 as a newly therapy target was revealed to be highly expressed within PAAD tissues in comparative with that in normal pancreatic tissues in public datasets. To confirm the cancer database accuracy, IHC experiments were performed on PAAD tissues to verify that CMTM6 was actually abnormally highly expressed within pancreatic cancer. Additionally, in participants with PAAD, increased expression of CMTM6 was linked to a worse projected outcome. To further illustrate how PAAD development is impacted by CMTM6’s oncological influence, a series of functional experiments was carried out to reveal that CMTM6 can promote proliferative, migrative, and invasive ability of pancreatic cells. The extremely elevated expression of CMTM6 and concomitant shorter survival time were also reported in other cancers, including gastric cancer, glioma, melanoma, breast cancer, hepatocellular carcinoma, lung cancer, oral squamous cell carcinoma, and colorectal cancer (Burr et al., 2017; Mezzadra et al., 2017; Zhang et al., 2021; Ubukata et al., 2021; Tanaka et al., 2019; Peng et al., 2021; Zhao et al., 2020; Guan et al., 2018; Tian et al., 2021; Xiao et al., 2021; Koh et al., 2019; Zugazagoitia et al., 2019; Shang et al., 2020; Wang et al., 2020; Hou et al., 2020; Liu et al., 2021; Yafune et al., 2013). These researches suggest that in several malignancies, CMTM6 may act as a prognostic indicator and a broad spectrum therapeutic target.

Notably, it has been suggested that CMTM6 plays crucial roles in the TME. The transmembrane protein CMTM6 has six transmembrane domains, is a tetrameric protein with two extracellular loop domains, and has a short MARVEL domain protein that extends to the cytoplasm at its N- and C-termini (Elazar & Peles, 2020). As a new regulator of PD-L1, CMTM6 co-localizes with the PD-L1 protein and stabilizes PD-L1 expression on the cell surface (Burr et al., 2017; Mezzadra et al., 2017). The anti-tumor response and immunological escape are caused by the stabilizing action of CMTM6 on PD-L1, which promotes PD-L1 on the tumor cell membrane and attaches to PD-1 on activated T cells (Kidokoro et al., 2020; Xie et al., 2019). Therefore, an increasing amount of research is focused on the interaction and association involving CMTM6 and PD-L1 in many cancers and even some inflammatory diseases. Researches have shown a significant positive association between CMTM6 and PD-L1 expression in gastric cancer, glioma, hepatocellular carcinoma, non-small cell lung cancer, and lung squamous carcinoma (Burr et al., 2017; Mezzadra et al., 2017; Zhang et al., 2021; Ubukata et al., 2021; Tanaka et al., 2019; Peng et al., 2021; Zhao et al., 2020; Guan et al., 2018; Tian et al., 2021; Xiao et al., 2021; Koh et al., 2019; Zugazagoitia et al., 2019; Shang et al., 2020; Wang et al., 2020; Hou et al., 2020; Liu et al., 2021; Yafune et al., 2013; Zhu et al., 2019). In melanoma, breast cancer, and thyroid cancer cell lines, CMTM6 was discovered to be an important regulator of the expression of PD-L1 (Burr et al., 2017; Mezzadra et al., 2017). In our research, we first demonstrated that CMTM6 regulating the expression of PD-L1 in PAAD. By using lentivirus shRNA to inhibit the expression of CMTM6, pancreatic cells’ PD-L1 expression was subsequently reduced. In pancreatic cell lines, CMTM6 may bind to the PD-L1 protein according to co-IP analysis. The important interaction connecting CMTM6 and PD-L1 in the TME indicates the significant immunological effect of CMTM6 in cancers.

To further investigate the immunological function of CMTM6 in the TME of PAAD, we performed GSEA enrichment analysis, including GO analysis, KEGG analysis, and WikiPathways, and revealed that the expression of CMTM6 was enriched in most regulation networks of immune responses. The correlation analysis showed that expression of CMTM6 was affirmatively related with a large amount of immunomodulators. Additionally, CMTM6 expression was shown to be associated with increased immunity cell infiltration, especially in the different types of T cells, macrophages, and aDC cells in TCGA-PAAD and three GEO databases. These results are consistent with those of previous research. For example, in lower-grade brain gliomas, Zhao et al. showed that enhanced CMTM6 expression was associated with increased immune infiltration (Guan et al., 2018). The expression of CMTM6 also was shown to be strongly correlated with numerous immune molecules, including PD-L1, CD3, CD20, and CD68. In the meanwhile, there was a strong correlation between the expression of CMTM6 and that of CD3+ T cells, CD20+ B lymphocytes, and CD68+ macrophages in melanoma (Burr et al., 2017; Mezzadra et al., 2017). In lung squamous carcinoma, the expression of CMTM6 was shown to be positively associated with the amount of infiltration of CD8+ T cells, macrophages, and dendritic cells but negatively correlated with CD4+ T cells (Martinez-Morilla et al., 2020). Additionally, there was a positive correlation between the CMTM6 expression and the infiltration of CD163+ macrophages in oral squamous cell carcinoma (OSCC) (Pang et al., 2021; Mohapatra et al., 2021; Ishigami et al., 2011). Next, we further analyzed seven metagenes to determine the function of CMTM6 within inflammatory reaction. The findings indicated that the expression of CMTM6 was affirmatively related with most inflammatory activities in TCGA and three representative GEO databases, suggesting that CMTM6 may be upregulated during T-cell signaling transduction, antigen-presenting cells, and macrophage activation. In summary, all of these results have been proposed that CMTM6 might be a viable immunotherapy target within PAAD.

This research is the first to report the malignant biological behavior and potential immune effect of CMTM6 within PAAD. However, there are still some limitations. Although we have verified the immunomodulatory effect of CMTM6 in TCGA-PAAD and some GEO datasets, the amounts of datasets are limited. And more basic experimental research is required to ascertain and verify the crucial immunological regulation mechanism of CMTM6 in PAAD. Recently, more and more studies have been reported the detailed mechanism of CMTM6 participating in regulating tumor immunity. For example, Ho. et al found that the CD58-CD2 axis is co-regulated with PD-L1 through CMTM6. Competition between CD58 and PD-L1 stability regulated by CMTM6 binding determines their rate of endosomal recycling over lysosomal degradation, which then further disturbing immune evasion through affecting T-cell activation, intratumoral T-cell infiltration, and proliferation (Ho et al., 2023). Moreover, in triple-negative breast cancer (TNBC), circ-0000512 has been revealed to inhibit PD-L1 ubiquitination by sponging the miR-622/CMTM6 axis, thus promoting TNBC progression and immune escape (Dong et al., 2023). In addition, CMTM6 also participates in regulation of macrophages, and OSCC cell-secreted exosomal CMTM6 induces M2-like macrophages polarization via ERK1/2 signaling pathway (Pang et al., 2021). More importantly, in pancreatic cancer, oncolytic peptide LTX-315 promotes anti-pancreatic cancer immunity by targeting the ATP11B-PD-L1 axis which is dependent of CMTM6-mediated lysosomal degradation (Tang et al., 2022). Therefore, based on available researches, we think there are several perspectives and speculative hypotheses worth considering regarding the specific mechanisms of CMTM6 in regulating tumor immunity: (1) Immune checkpoint modulation: CMTM6 may interact with other immune checkpoint molecules, not only PD-L1, then influencing the balance between immune activation and immune tolerance in the TME; (2) tumor-associated antigen presentation: CMTM6 might play a role in the presentation of tumor-associated antigens by modulating the expression or function of MHC molecules. This could affect the recognition and response of tumor-specific T cells, thereby impacting anti-tumor immune responses; (3) tumor-infiltrating lymphocyte (TIL) regulation: CMTM6 could be involved in the recruitment, activation, or suppression of TILs within the TME. It may interact with chemokines, adhesion molecules, or other immune cell regulators to modulate the composition and function of TILs, influencing the overall anti-tumor immune response; (4) interplay with other immune regulatory pathways: CMTM6 may also interact with various immune regulatory pathways, such as cytokine signaling, NF-κB pathway, or T-cell co-stimulatory pathways, to shape the immune landscape in the TME. Last but not least, although we displayed that higher CMTM6 expression may represent better immunotherapeutic response in various public databases with a strong tendency, the effective *P* value was not below 0.05. We suspect this may be due to the fact that the existing immunotherapy gene set is for melanoma or other cancers, not pancreatic cancer. In addition, the effects of different immunotherapy drugs on CMTM6 are also unpredictable. Therefore, the results we have presented so far are very limited and only serve as a reference for general direction. In the future, more immunotherapy datasets, more powerful experimental validation, and statistical data are needed to confirm the influence and prediction of CMTM6 against anti-PD-L1/PD-1, anti-CTLA-4, and other immunotherapy efficacy.

## Conclusions

Taken together, our results reveal that CMTM6 is upregulated within PAAD. Elevated expression of CMTM6 is related with less effective clinical outcomes and worse prognostic status. Furthermore, CMTM6 forms an immunological and inflammatory TME within PAAD patients. CMTM6 is also involved in immune cell activation and tumor immune responses. These outcomes illustrate the significance of CMTM6 as a potential immunotherapy target for PAAD. We believe that CMTM6 can be widely explored and found more immune effects in the near future and can be actively and reasonably applied in clinical for the cancer treatment.

### Supplementary information

ESM 1 (PDF 480 kb)

ESM 2 (PDF 468 kb)

ESM 3 (PDF 559 kb)

ESM 4 (PDF 1016 kb)

ESM 5 (PDF 654 kb)

ESM 6 (ZIP 1481 kb)

ESM 7 (XLSX 10 kb)

ESM 8 (XLSX 9 kb)

ESM 9 (XLSX 9 kb)

ESM 10 (XLSX 9 kb)

ESM 11 (XLSX 9 kb)

ESM 12 (XLSX 11 kb)

## Data Availability

Publicly available datasets were analyzed in this study. The datasets TCGA-PAAD and the corresponding clinical patient information analyzed for this study can be found in the TCGA Knowledge Base (https://portal.gdc.cancer.gov/projects/TCGA-PAAD, accessed on 23 December 2022, dbGaP Study Accession: phs000178). Gene expression information from GSE85916, GSE78229, GSE62452, GSE57495, GSE28735, and GSE102238 datasets from the database of GEO can be downloaded in https://www.ncbi.nlm.nih.gov. All original analysis datasets used or analyzed during the current study are available from the corresponding author on reasonable request.

## Abbreviations

PAAD: pancreatic adenocarcinoma
TME: tumor microenvironment
GSEA: gene set enrichment assessment
CMTM6: CKLF-like MARVEL transmembrane domain containing 6
PD-L1: programed cell apoptosis 1 ligand 1
TCGA: the Cancer Genome Atlas
GEO: Gene Expression omnibus
ROC: receiver operating characteristic
AUC: area under the curve
CCLE: Cancer Cell Line Encyclopedia
qRT-PCR: quantitative reverse transcription polymerase chain reaction
HR: hazard ratio
OS: overall survival
DSS: disease-specific survival
PFI: progression-free interval
SNV: single nucleotide variant
SNP: single nucleotide polymorphisms
CNA: copy number alterations
GO: Gene Ontology
KEGG: Kyoto Encyclopedia of Genes and Genomes
NES: normalized enrichment score
NK: natural killer
TLR: Toll-like receptor
MHC: major histocompatibility complex
ssGSEA: enrichment of gene sets within individual samples
co-IP: co-immunoprecipitation
GTEx: Genotype-Tissue Expression
CNV: copy number variation
ATCC: American Type Culture Collection
RPMI: Roswell Park Memorial Institute
FBS: fetal bovine serum
DMEM: Dulbecco’s Modified Eagle Medium
IMDM: Iscove’s modified Dulbecco’s Medium
GAPDH: glyceraldehyde 3-phosphate dehydrogenase
RIPA: radioimmunoprecipitation
PMSF: phenylmethanesulfonyl fluoride
SDS-PAGE: sodium dodecyl sulfate-polyacrylamide gel electrophoresis
PVDF: polyvinylidene difluoride
TBST: Tris-buffered saline with 0.1% Tween-20
PBS: phosphate-buffered saline
OSCC: oral squamous cell carcinoma
TNBC: triple-negative breast cancer
TIL: tumor-infiltrating lymphocyte
