# Supplementary figures and images for: CMTM6 as a potential therapy target is associated with immunological tumor microenvironment and can promote migration and invasion in pancreatic adenocarcinoma
Source: Funct Integr Genomics. 2023 Sep 20;23(4):306. doi: 10.1007/s10142-023-01235-5 (PMC10509136; doi:10.1007/s10142-023-01235-5)

Fig S1

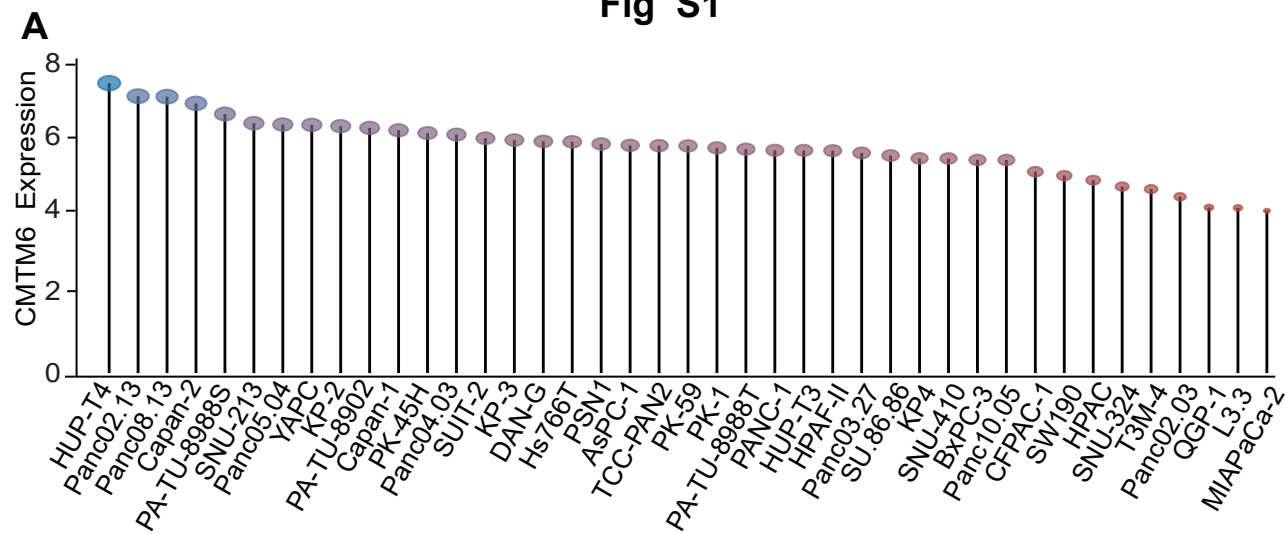

Supplement: Supplementary file 1 — (PDF 480 kb) [file 10142_2023_1235_MOESM1_ESM.pdf]

Fig S2

A

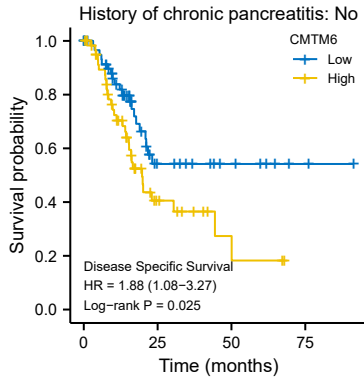

B

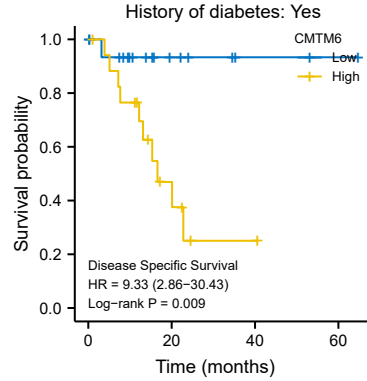

C

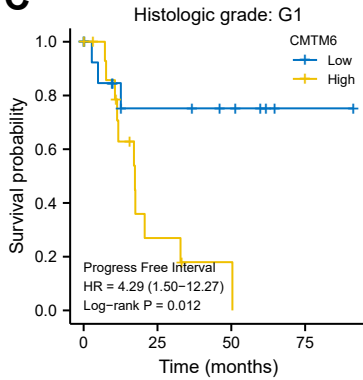

D

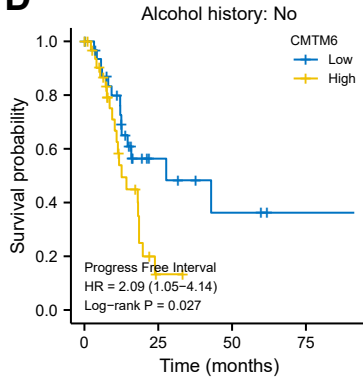

E

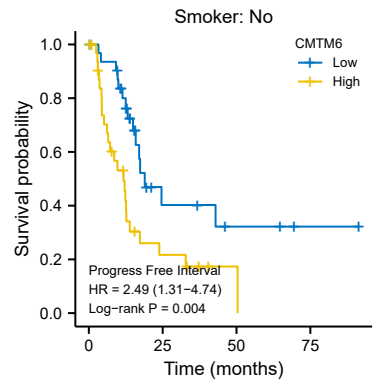

Supplement: Supplementary file 2 — (PDF 468 kb) [file 10142_2023_1235_MOESM2_ESM.pdf]

# Fig S3

## A

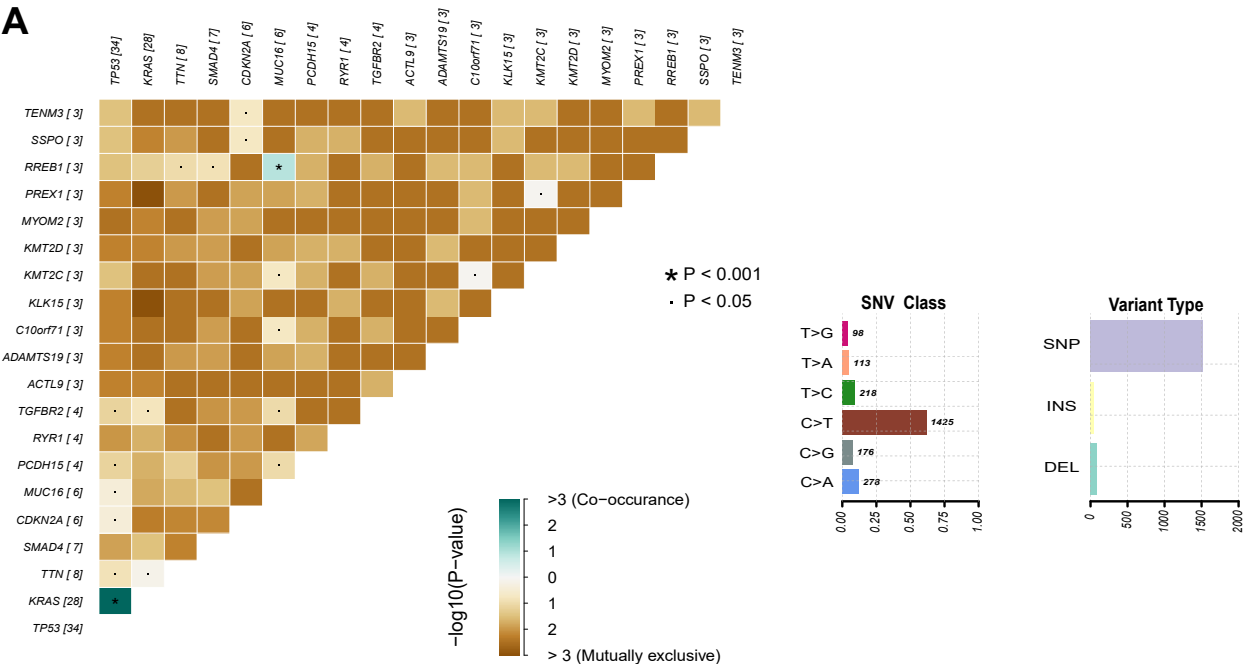

## B

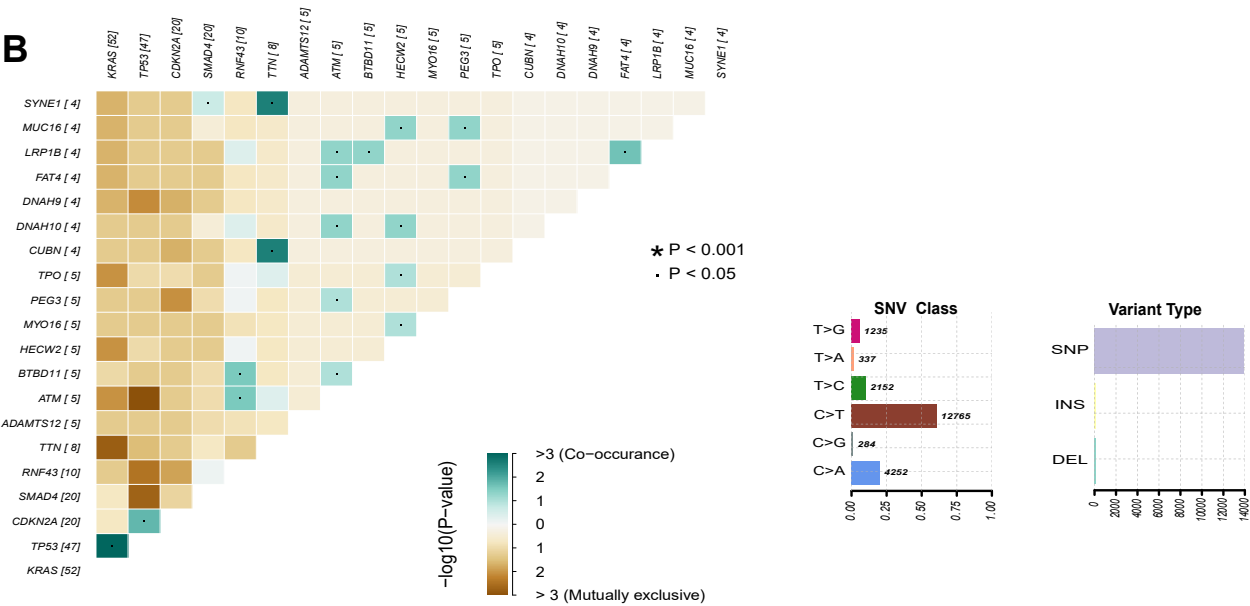

Supplement: Supplementary file 3 — (PDF 559 kb) [file 10142_2023_1235_MOESM3_ESM.pdf]

**Fig S4**

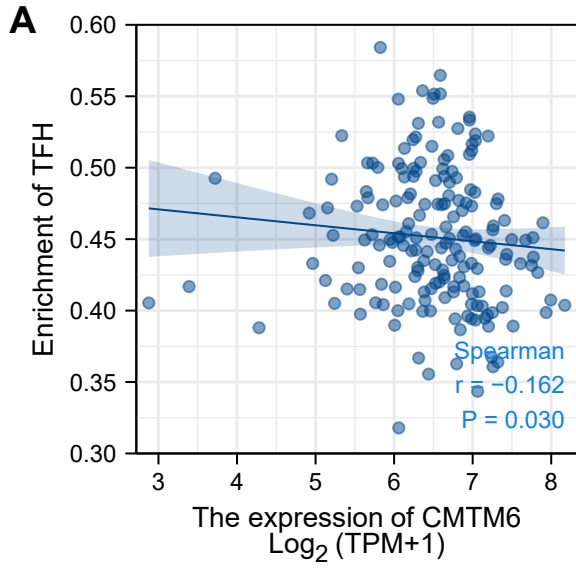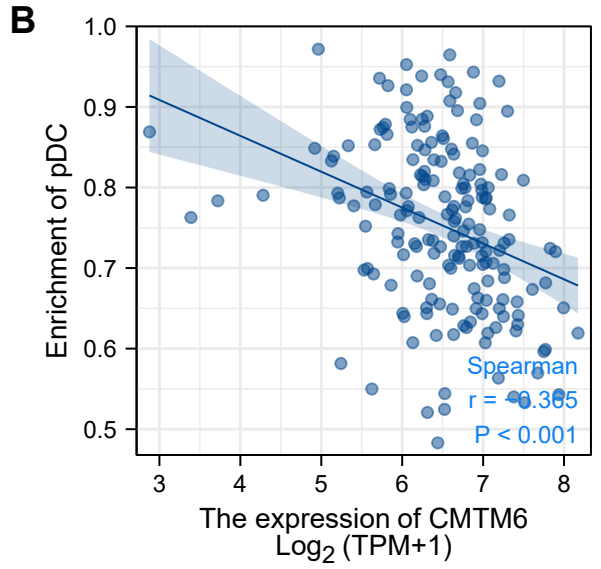

Supplement: Supplementary file 4 — (PDF 1016 kb) [file 10142_2023_1235_MOESM4_ESM.pdf]

**Fig S5**

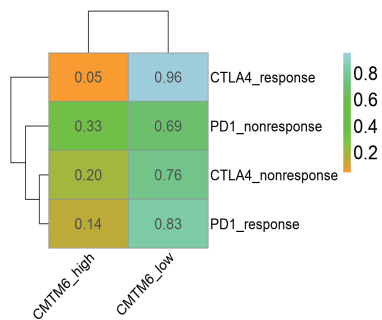

**TCGA**

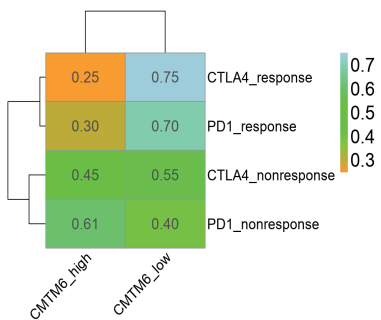

**GSE85916**

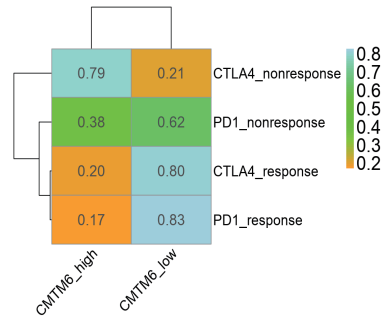

**GSE57495**

Supplement: Supplementary file 5 — (PDF 654 kb) [file 10142_2023_1235_MOESM5_ESM.pdf]

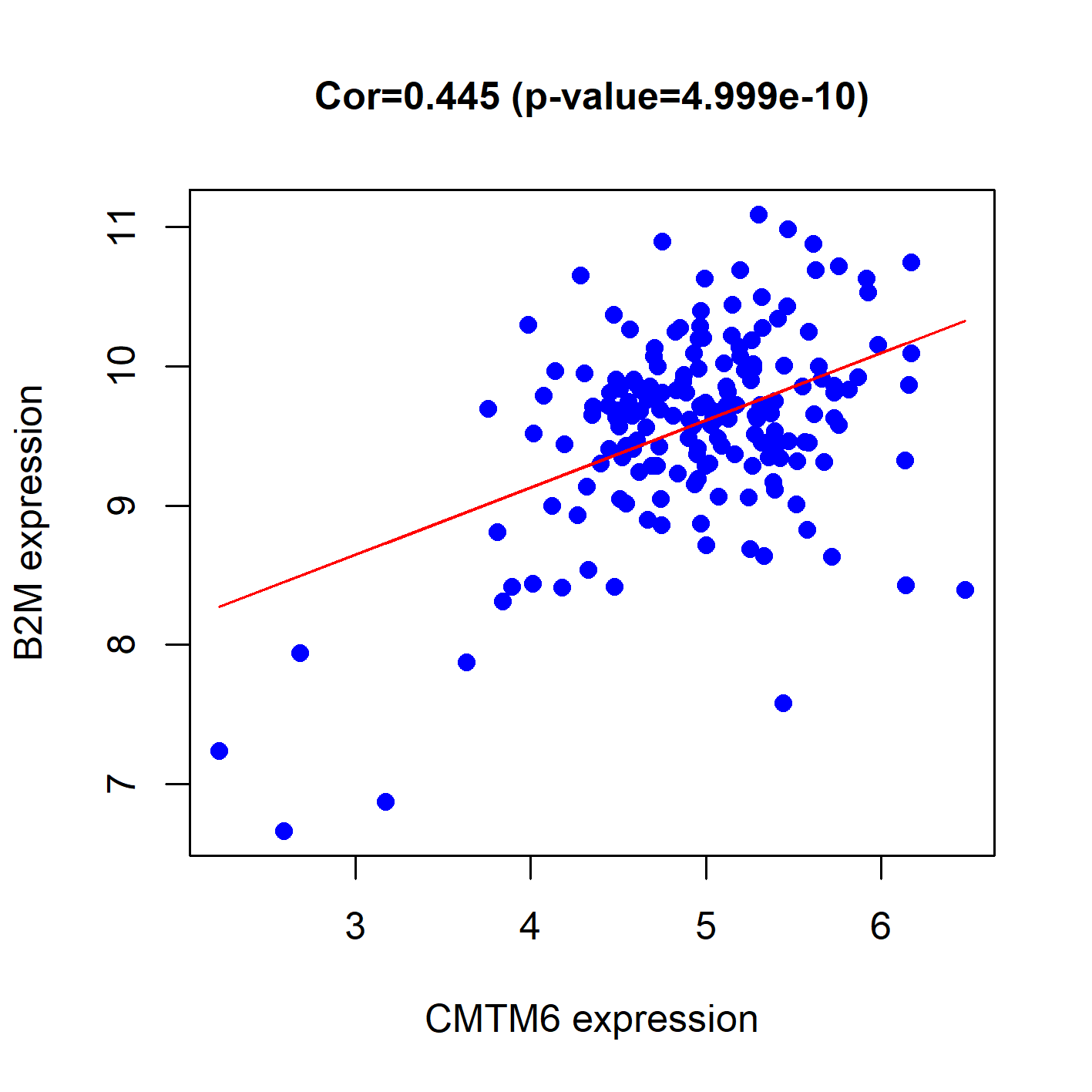

Supplement: Supplementary file 6 — (ZIP 1481 kb) [file 10142_2023_1235_MOESM6_ESM.zip › Supplement File 1/CMTM6_B2M.cor.tiff]

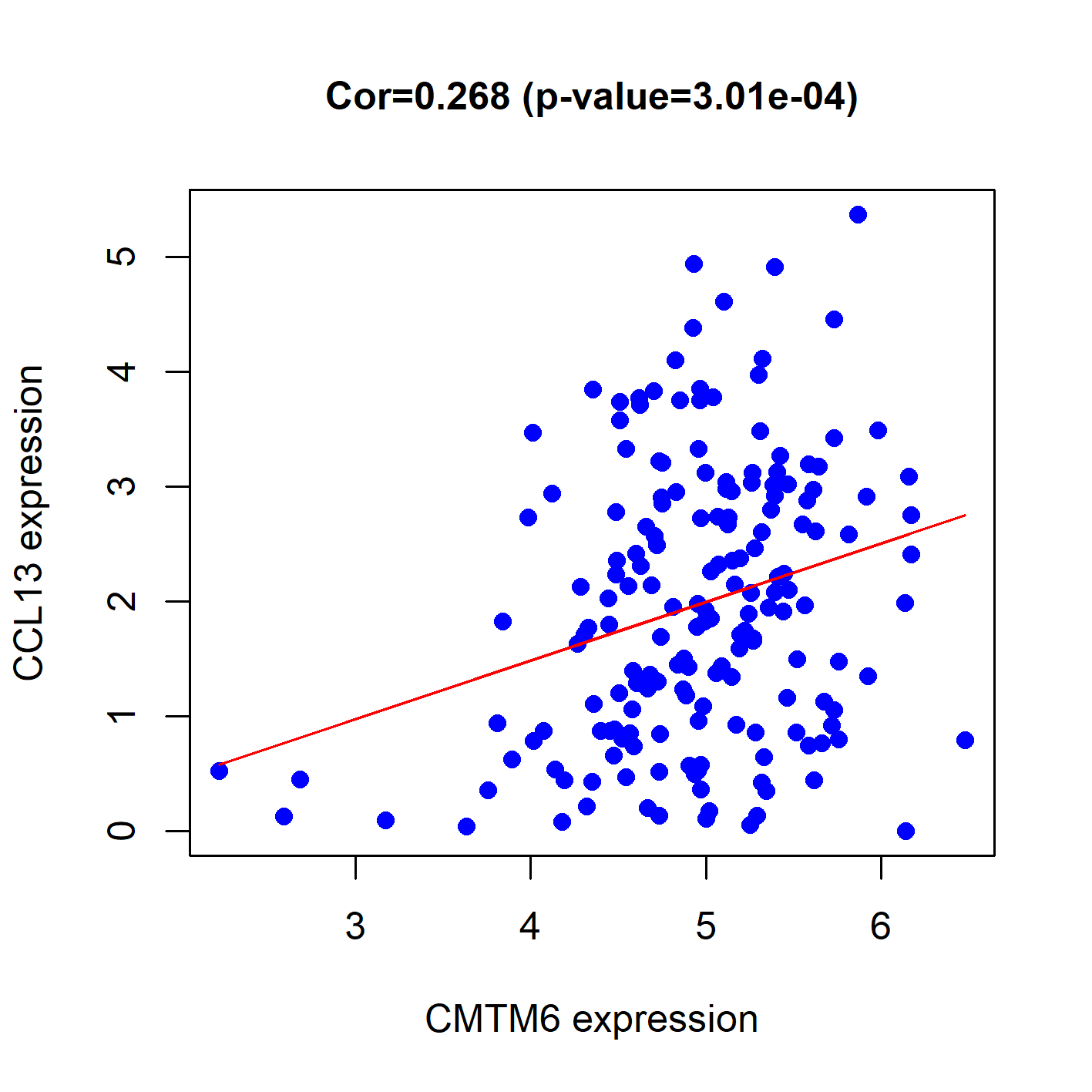

Supplement: Supplementary file 6 — (ZIP 1481 kb) [file 10142_2023_1235_MOESM6_ESM.zip › Supplement File 1/CMTM6_CCL13.cor.tiff]

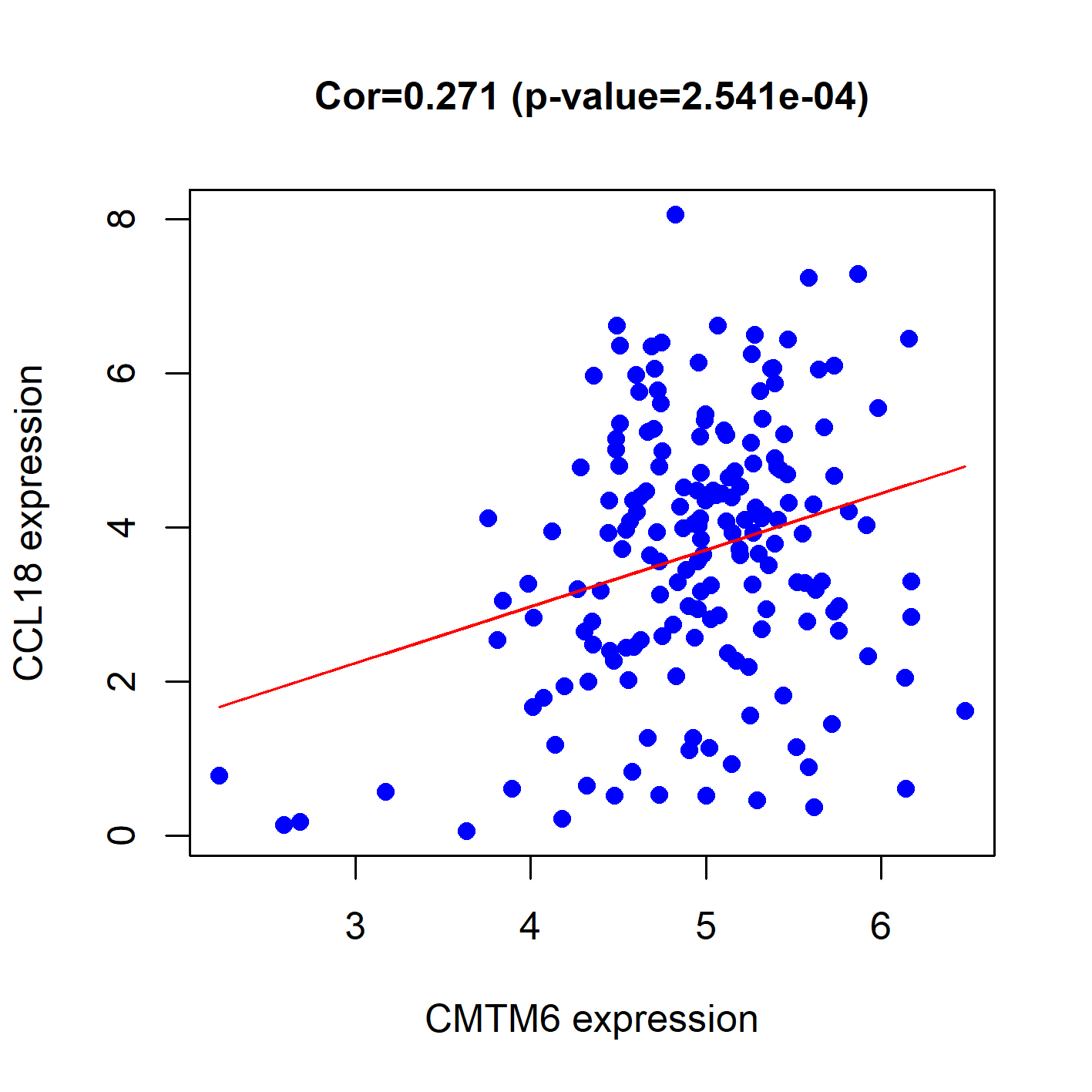

Supplement: Supplementary file 6 — (ZIP 1481 kb) [file 10142_2023_1235_MOESM6_ESM.zip › Supplement File 1/CMTM6_CCL18.cor.tiff]

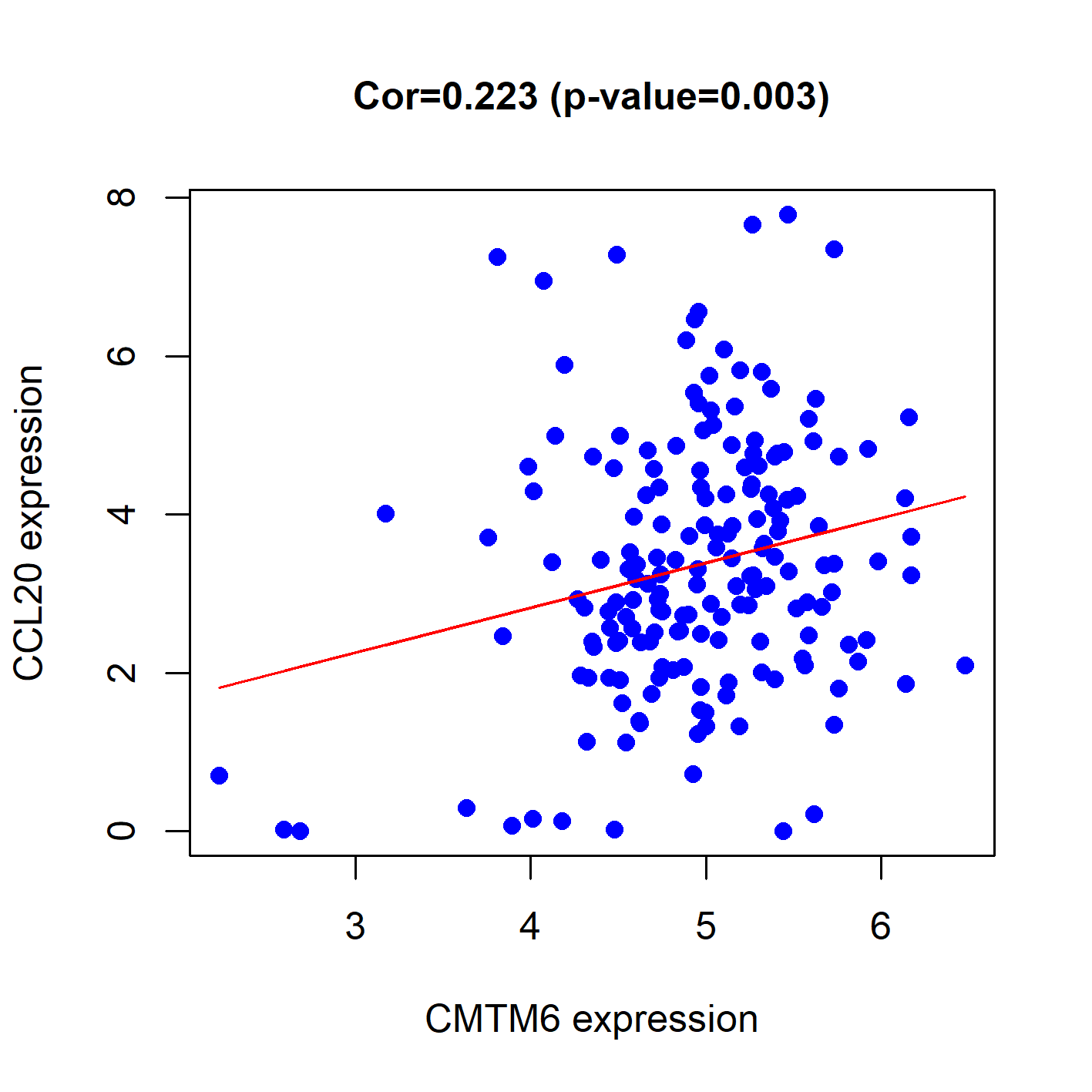

Supplement: Supplementary file 6 — (ZIP 1481 kb) [file 10142_2023_1235_MOESM6_ESM.zip › Supplement File 1/CMTM6_CCL20.cor.tiff]

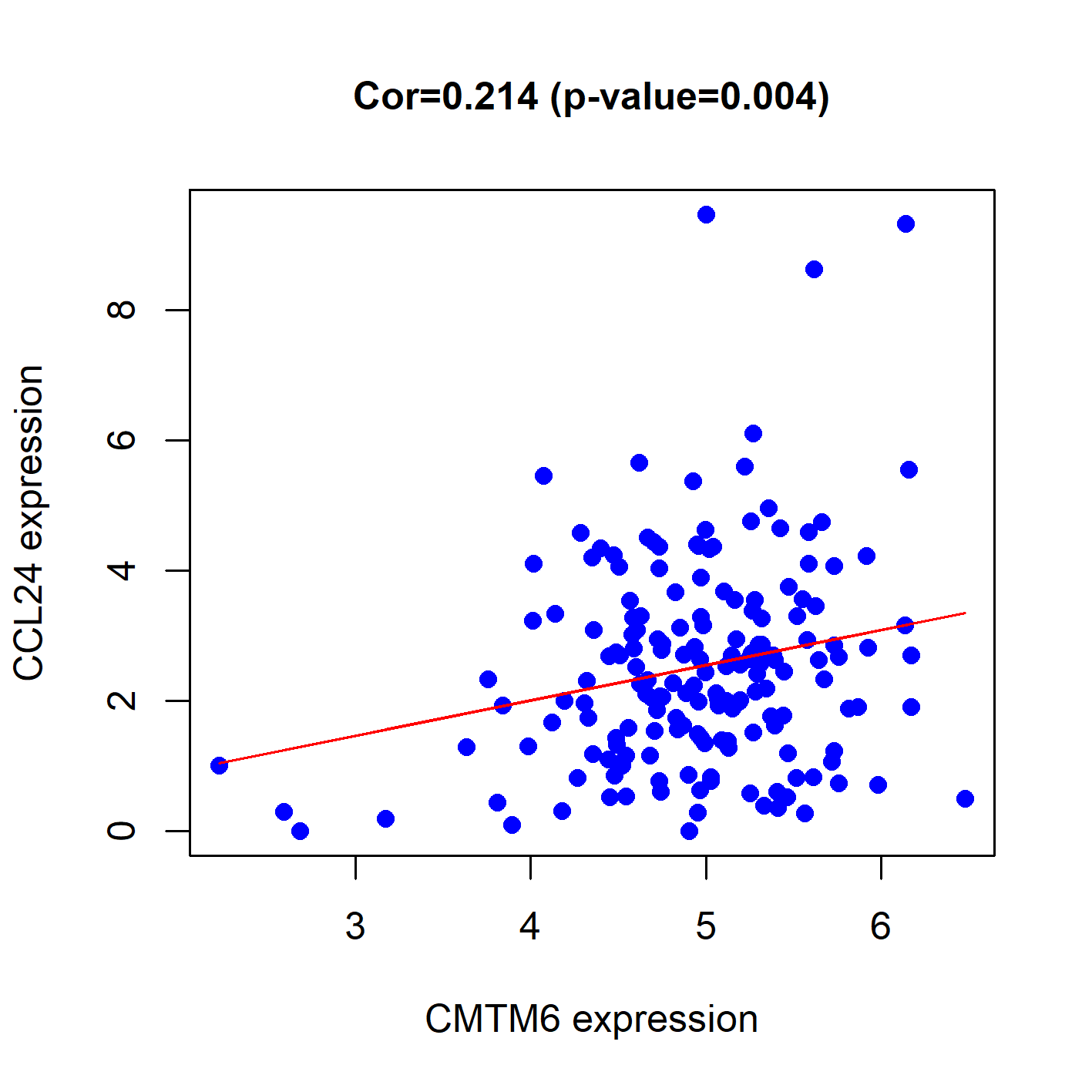

Supplement: Supplementary file 6 — (ZIP 1481 kb) [file 10142_2023_1235_MOESM6_ESM.zip › Supplement File 1/CMTM6_CCL24.cor.tiff]

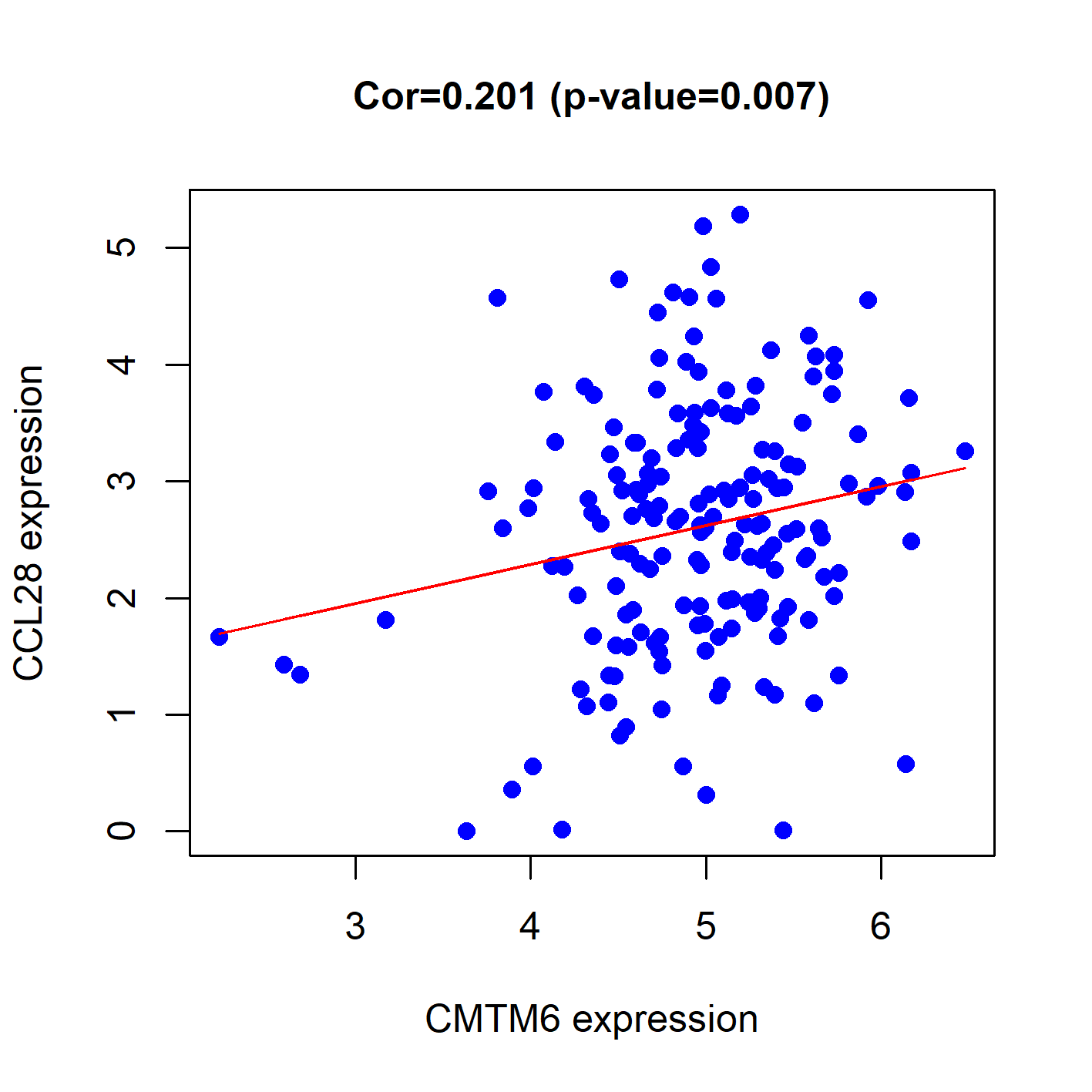

Supplement: Supplementary file 6 — (ZIP 1481 kb) [file 10142_2023_1235_MOESM6_ESM.zip › Supplement File 1/CMTM6_CCL28.cor.tiff]

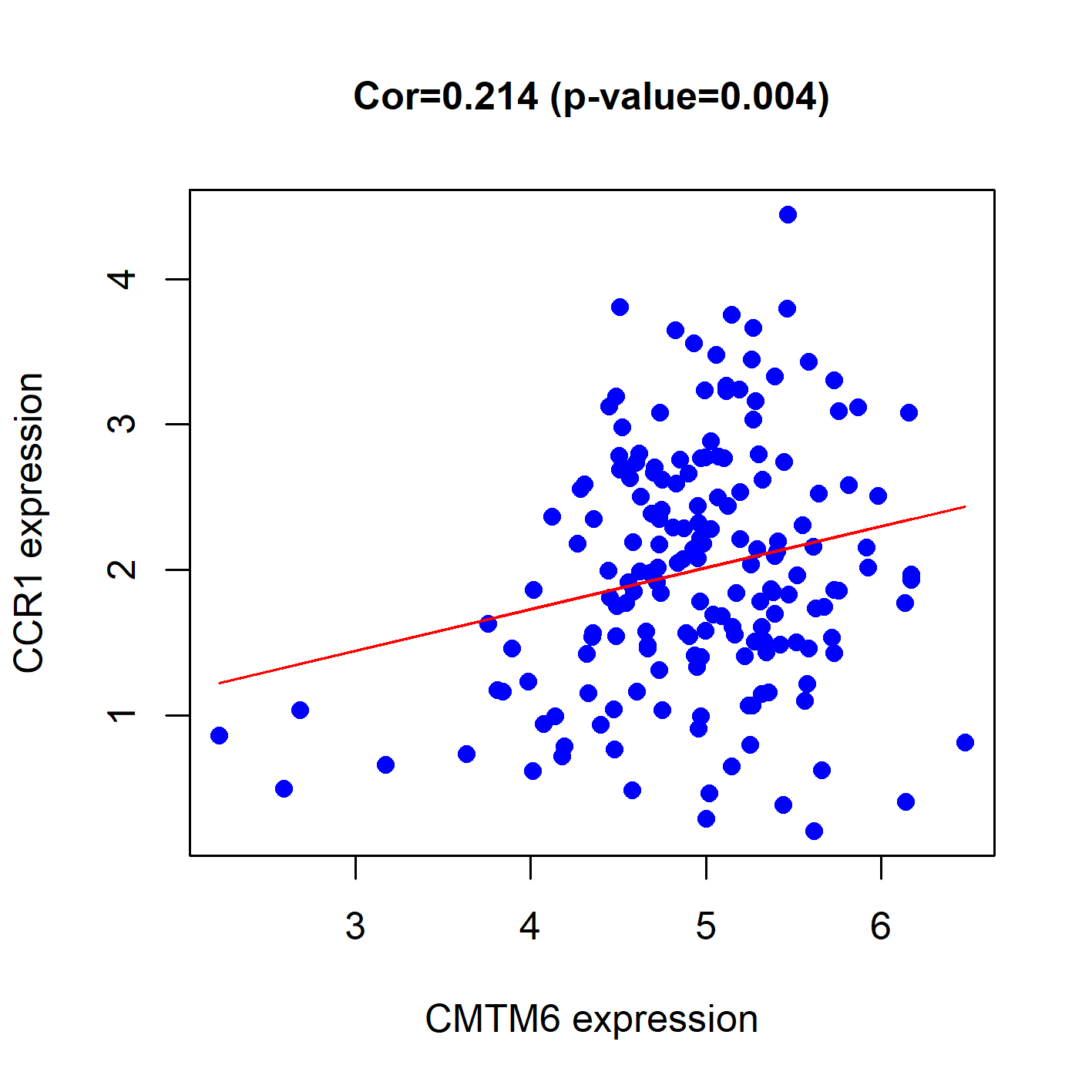

Supplement: Supplementary file 6 — (ZIP 1481 kb) [file 10142_2023_1235_MOESM6_ESM.zip › Supplement File 1/CMTM6_CCR1.cor.tiff]

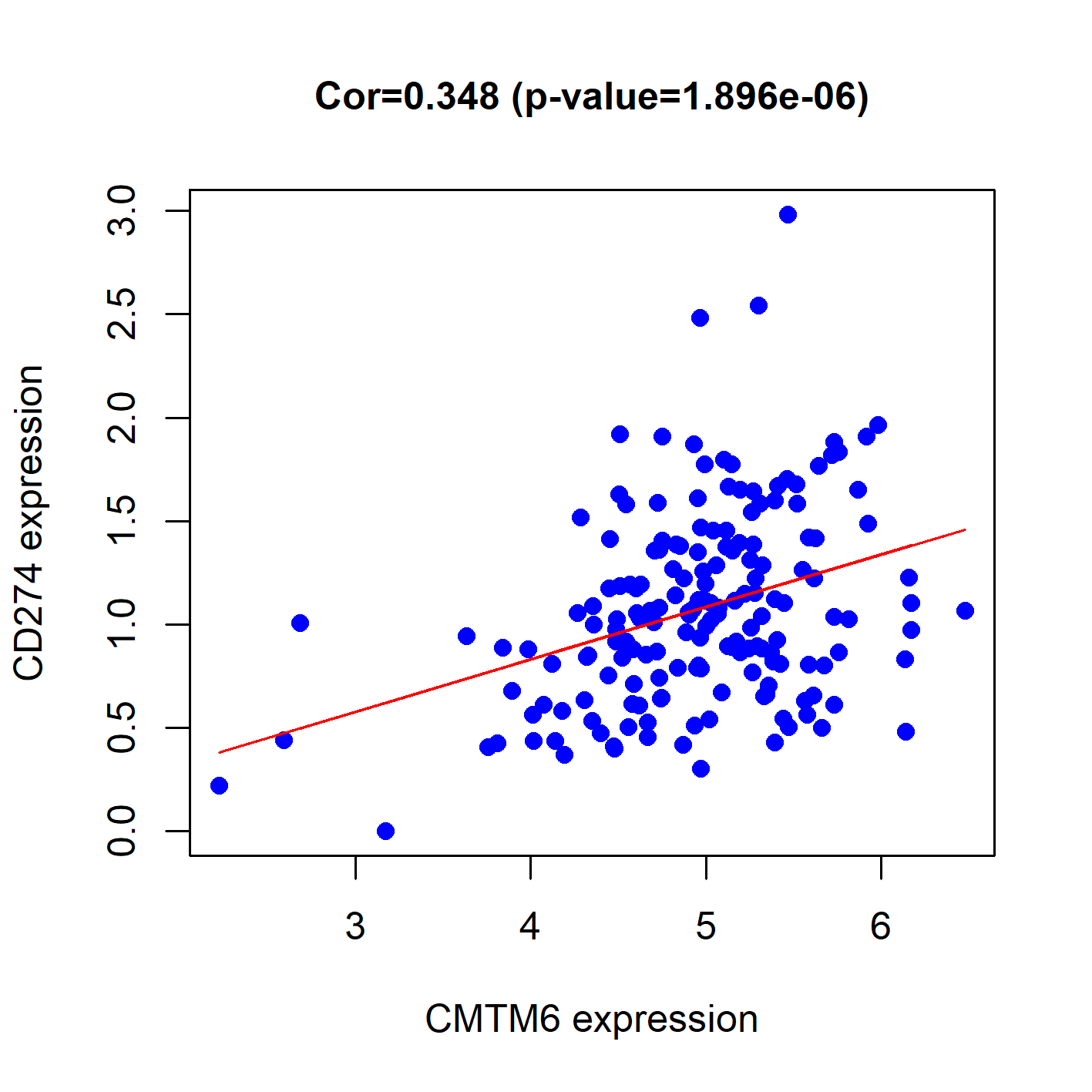

Supplement: Supplementary file 6 — (ZIP 1481 kb) [file 10142_2023_1235_MOESM6_ESM.zip › Supplement File 1/CMTM6_CD274.cor.tiff]

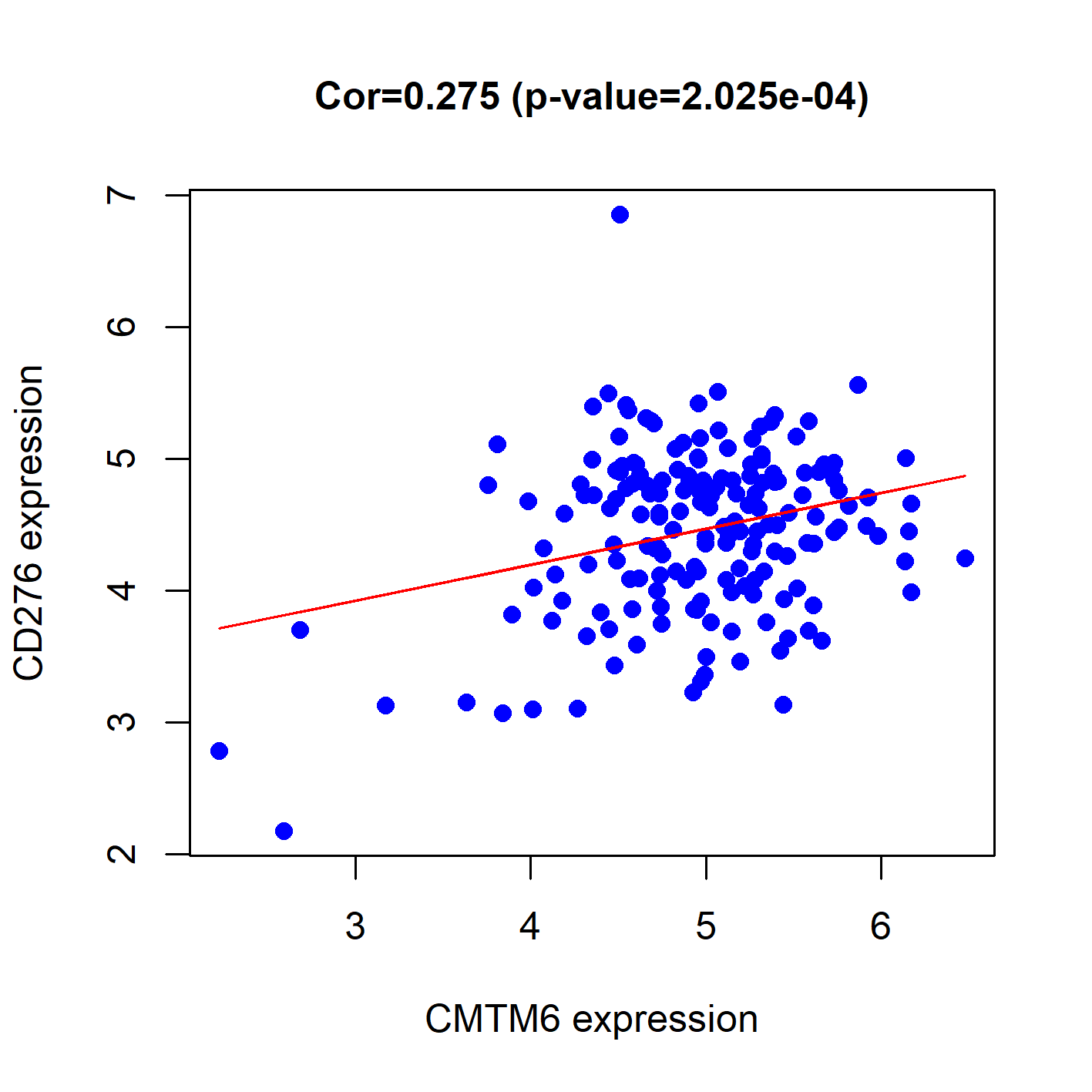

Supplement: Supplementary file 6 — (ZIP 1481 kb) [file 10142_2023_1235_MOESM6_ESM.zip › Supplement File 1/CMTM6_CD276.cor.tiff]

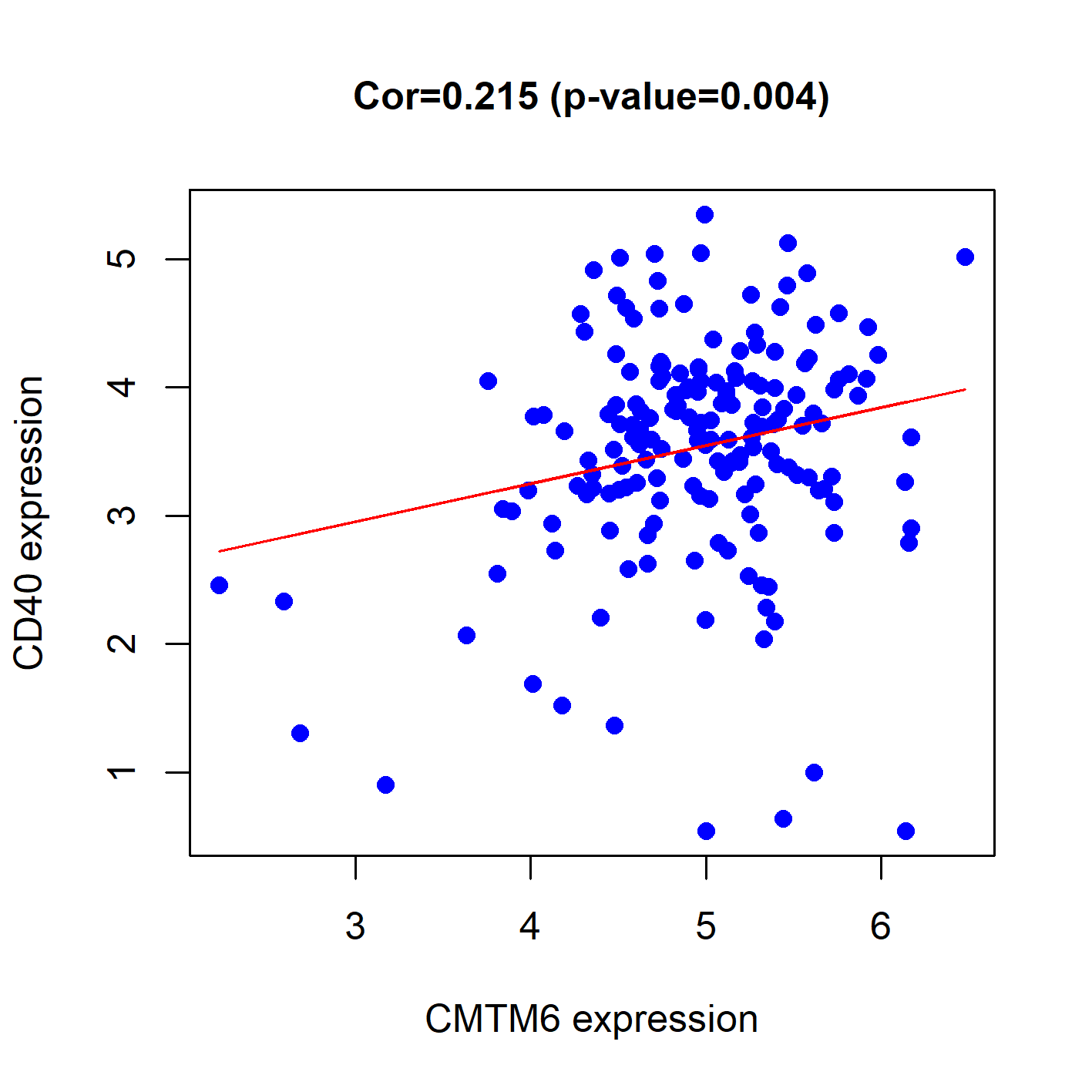

Supplement: Supplementary file 6 — (ZIP 1481 kb) [file 10142_2023_1235_MOESM6_ESM.zip › Supplement File 1/CMTM6_CD40.cor.tiff]

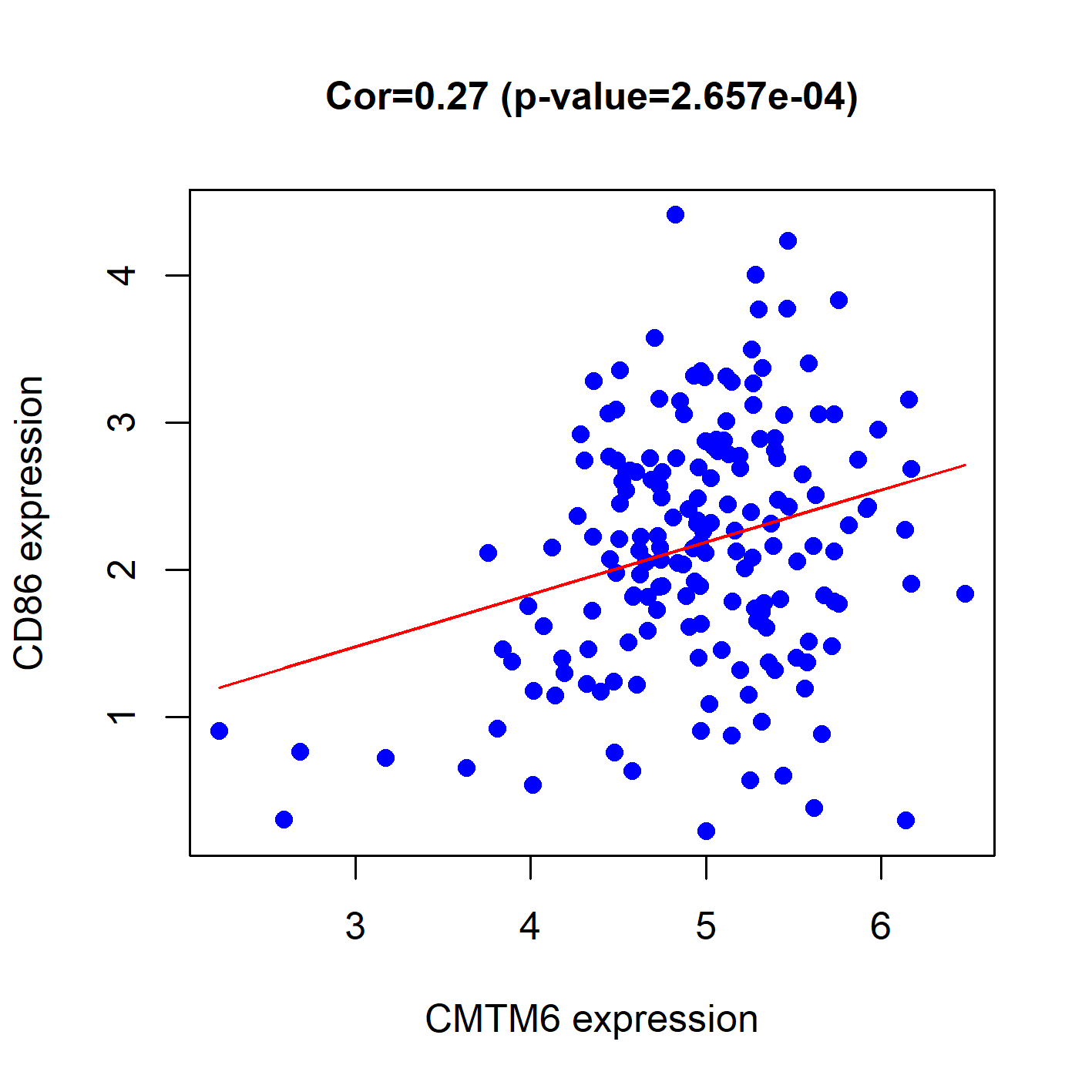

Supplement: Supplementary file 6 — (ZIP 1481 kb) [file 10142_2023_1235_MOESM6_ESM.zip › Supplement File 1/CMTM6_CD86.cor.tiff]

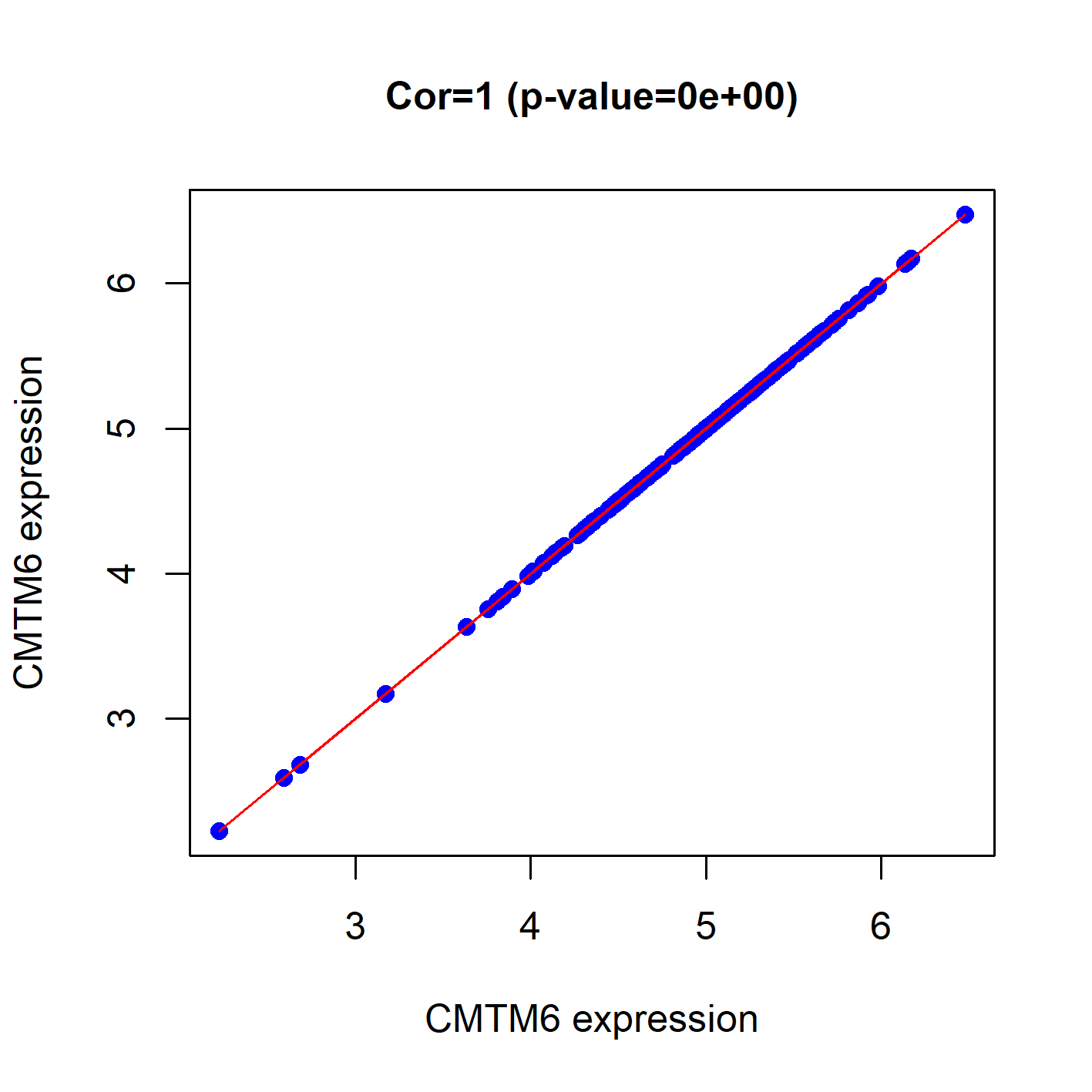

Supplement: Supplementary file 6 — (ZIP 1481 kb) [file 10142_2023_1235_MOESM6_ESM.zip › Supplement File 1/CMTM6_CMTM6.cor.tiff]

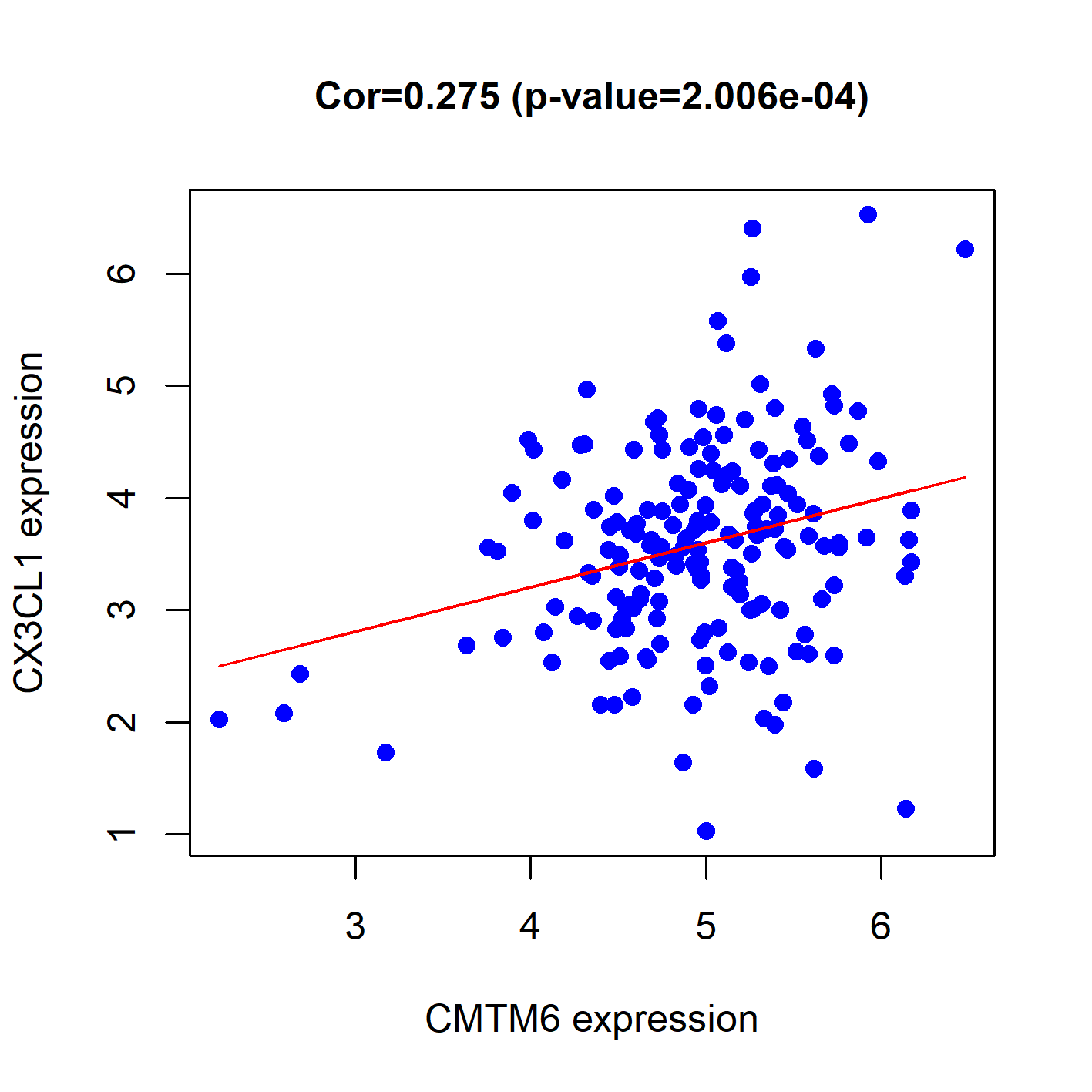

Supplement: Supplementary file 6 — (ZIP 1481 kb) [file 10142_2023_1235_MOESM6_ESM.zip › Supplement File 1/CMTM6_CX3CL1.cor.tiff]

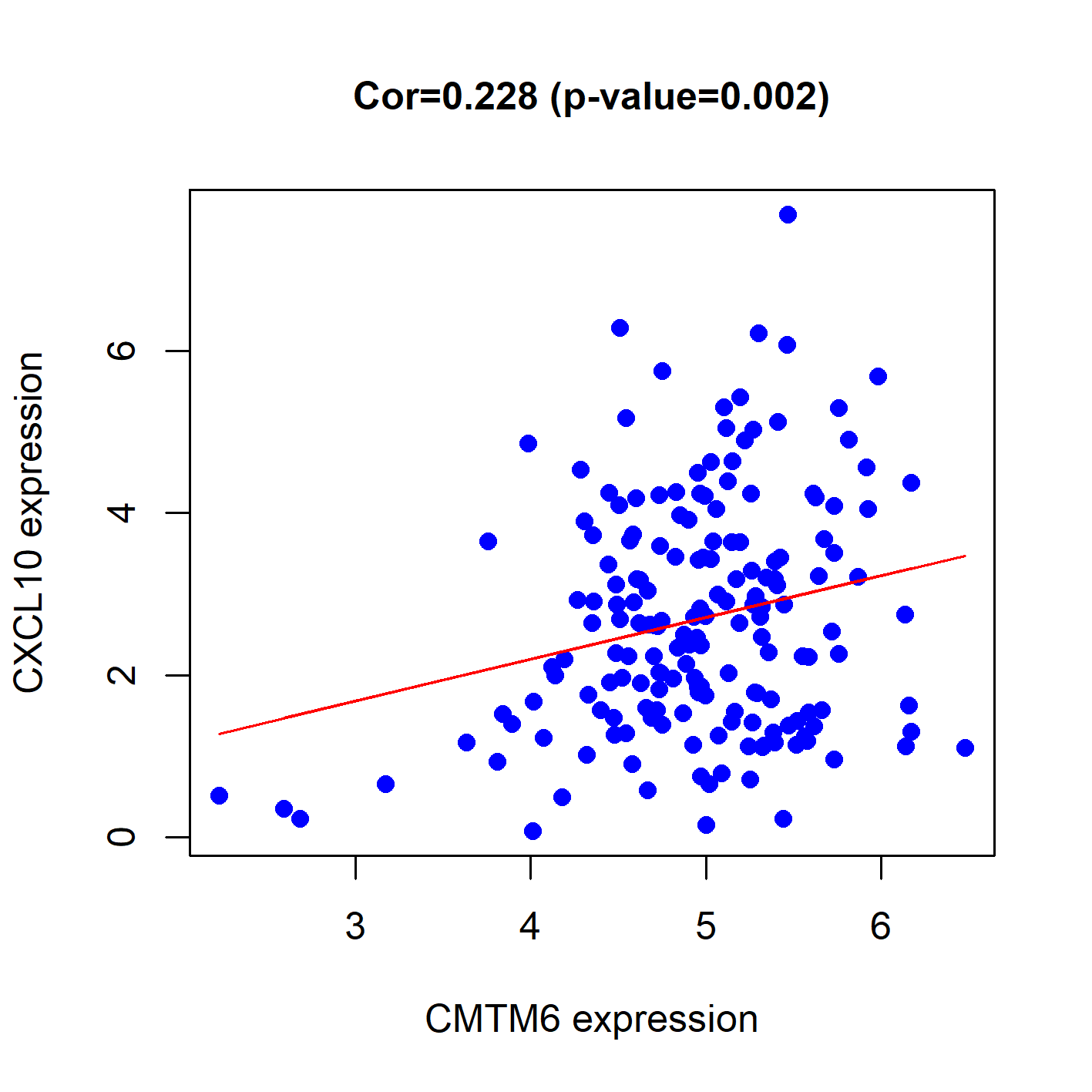

Supplement: Supplementary file 6 — (ZIP 1481 kb) [file 10142_2023_1235_MOESM6_ESM.zip › Supplement File 1/CMTM6_CXCL10.cor.tiff]

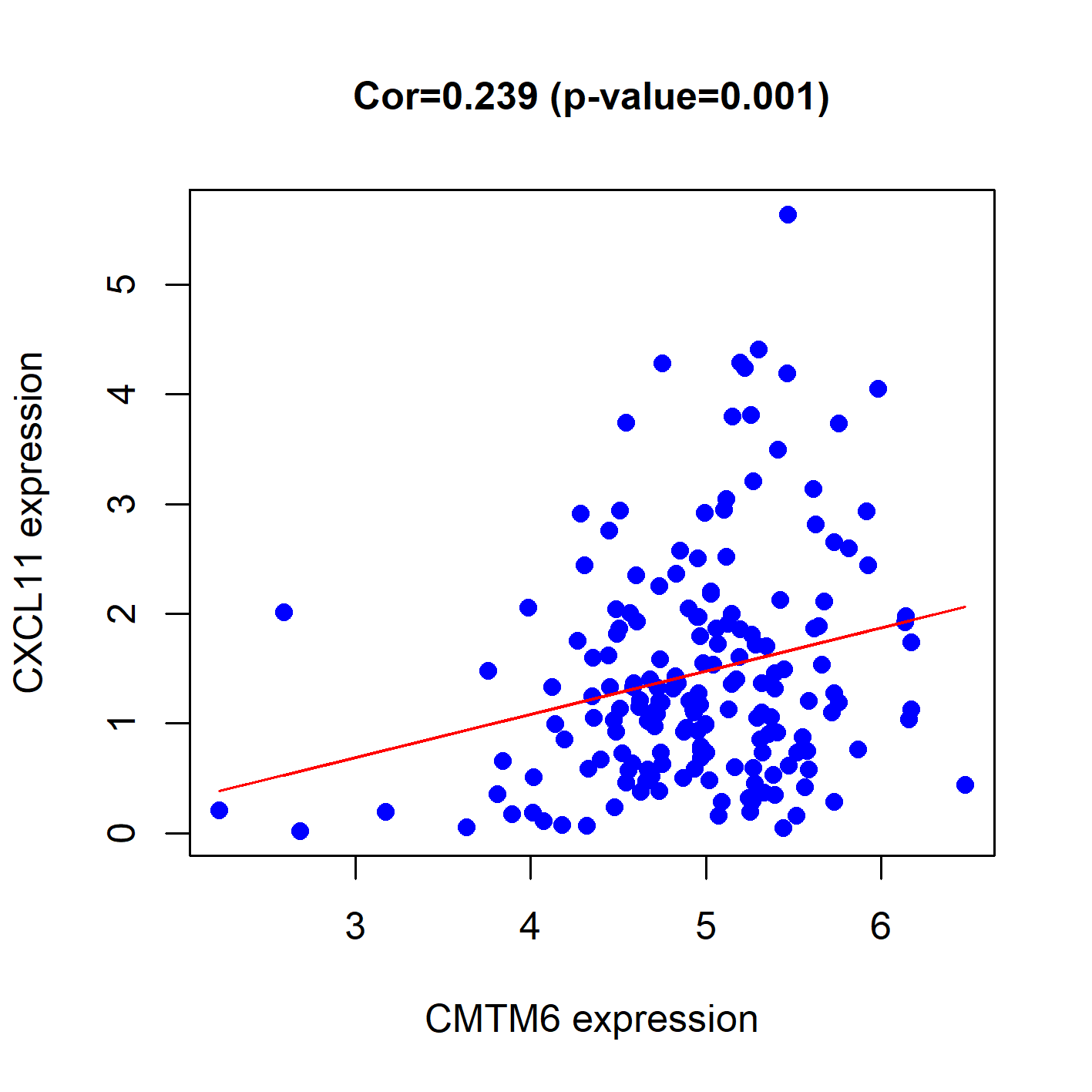

Supplement: Supplementary file 6 — (ZIP 1481 kb) [file 10142_2023_1235_MOESM6_ESM.zip › Supplement File 1/CMTM6_CXCL11.cor.tiff]

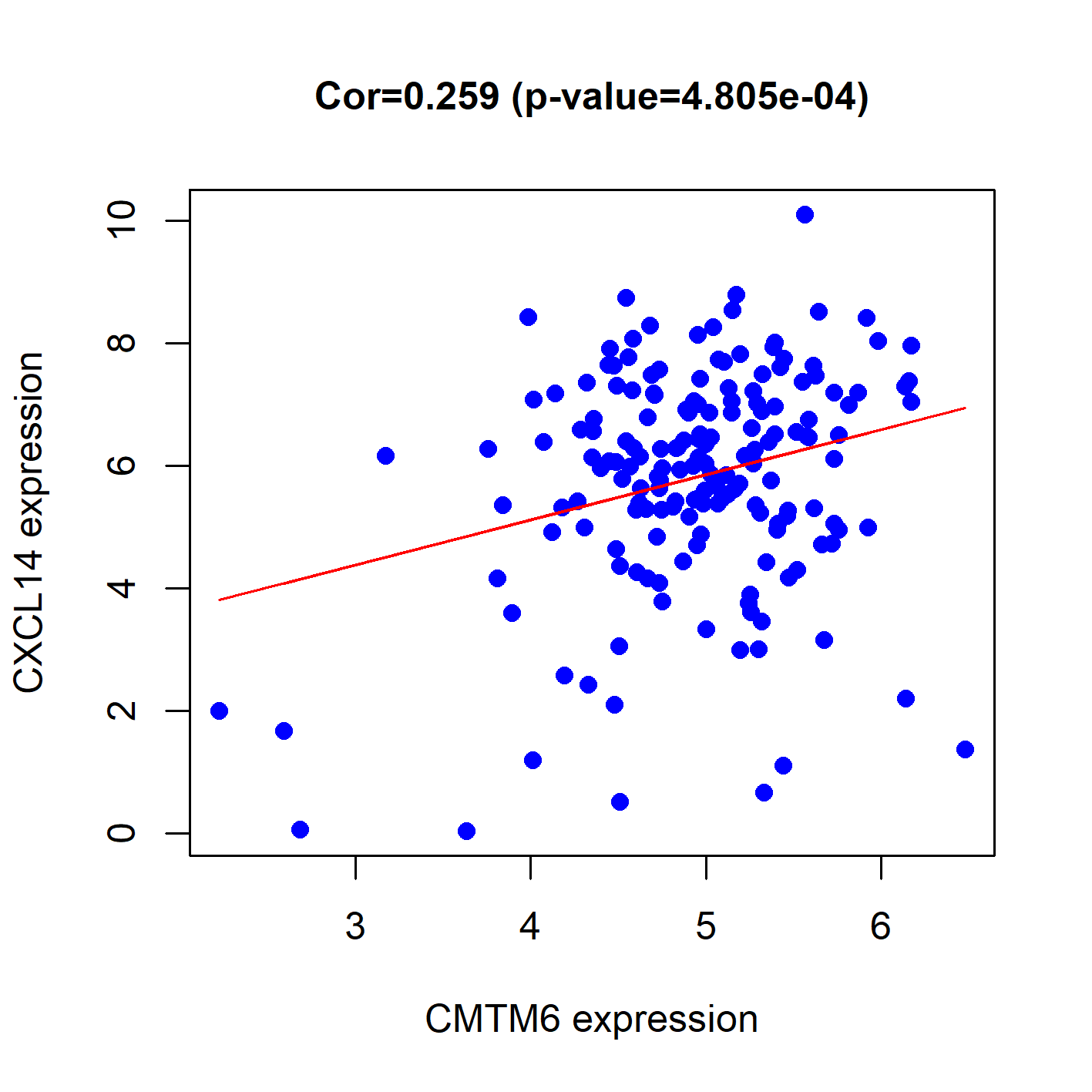

Supplement: Supplementary file 6 — (ZIP 1481 kb) [file 10142_2023_1235_MOESM6_ESM.zip › Supplement File 1/CMTM6_CXCL14.cor.tiff]

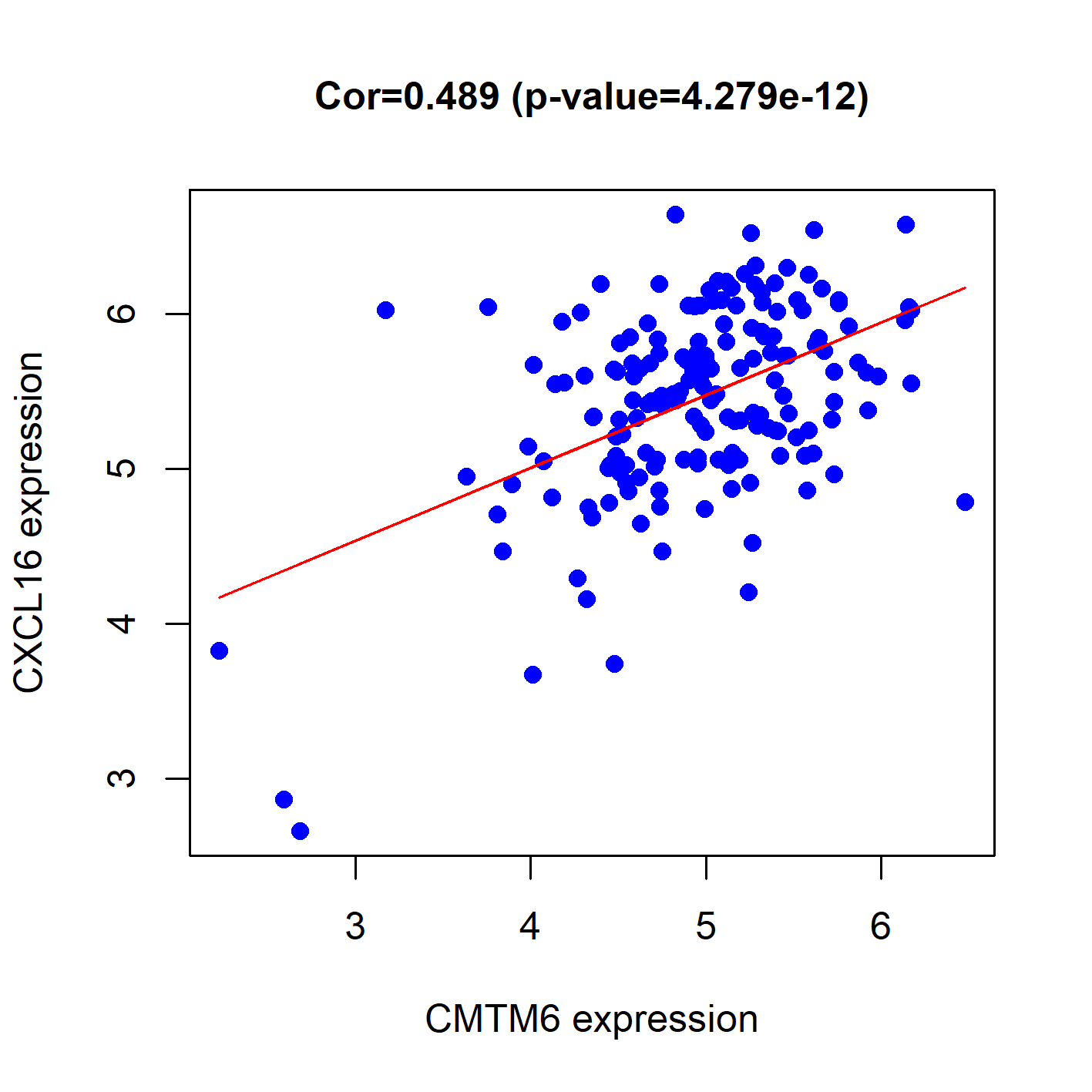

Supplement: Supplementary file 6 — (ZIP 1481 kb) [file 10142_2023_1235_MOESM6_ESM.zip › Supplement File 1/CMTM6_CXCL16.cor.tiff]

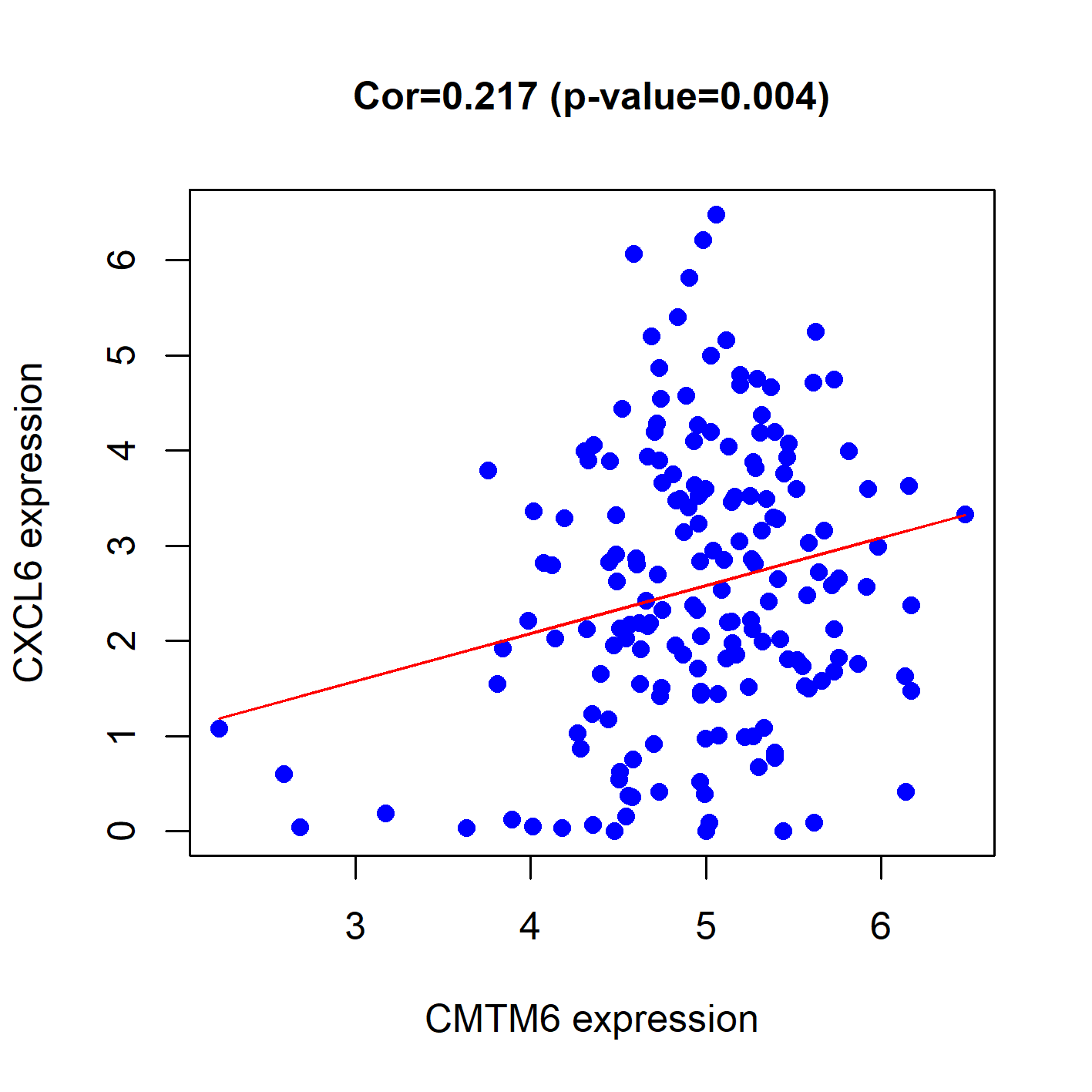

Supplement: Supplementary file 6 — (ZIP 1481 kb) [file 10142_2023_1235_MOESM6_ESM.zip › Supplement File 1/CMTM6_CXCL6.cor.tiff]

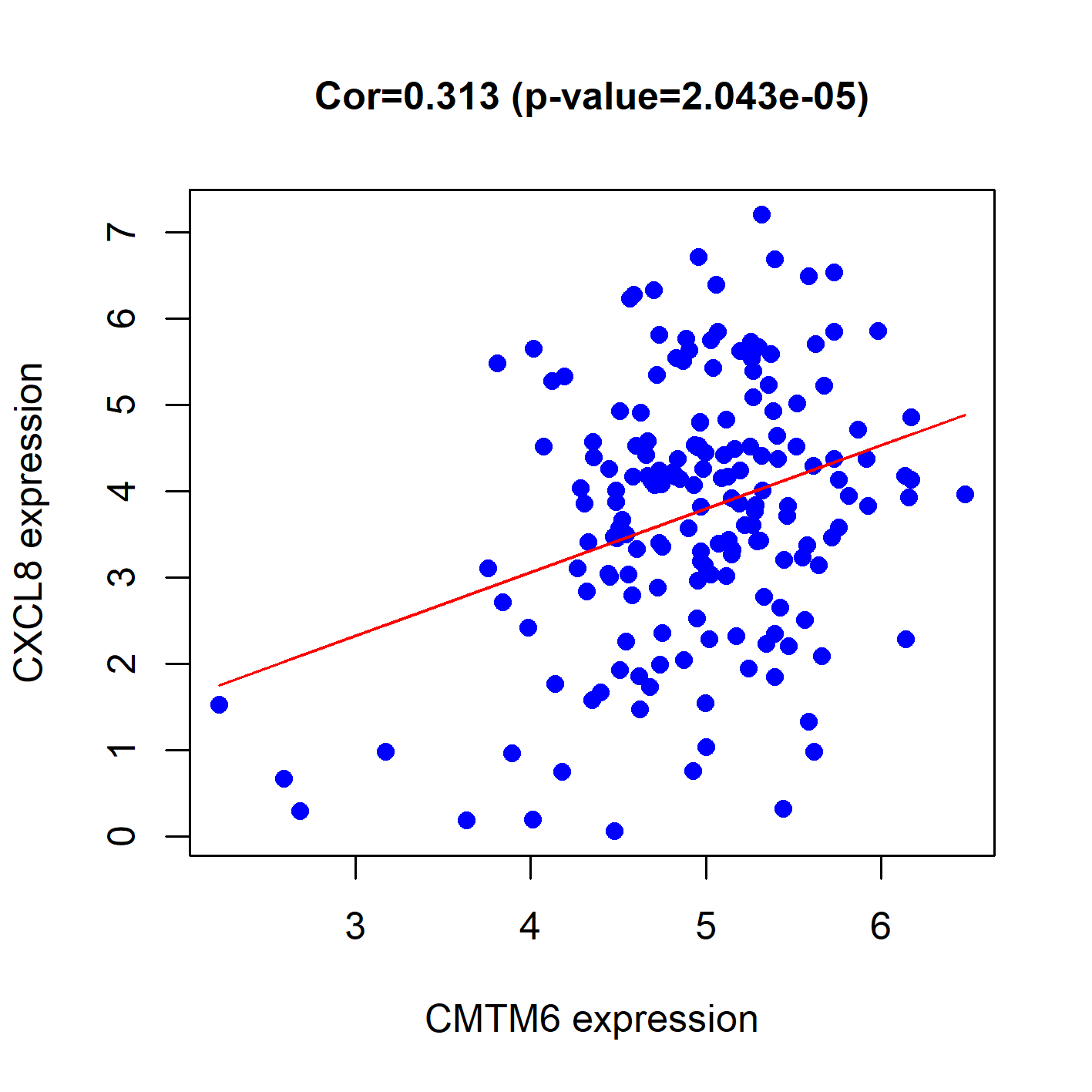

Supplement: Supplementary file 6 — (ZIP 1481 kb) [file 10142_2023_1235_MOESM6_ESM.zip › Supplement File 1/CMTM6_CXCL8.cor.tiff]

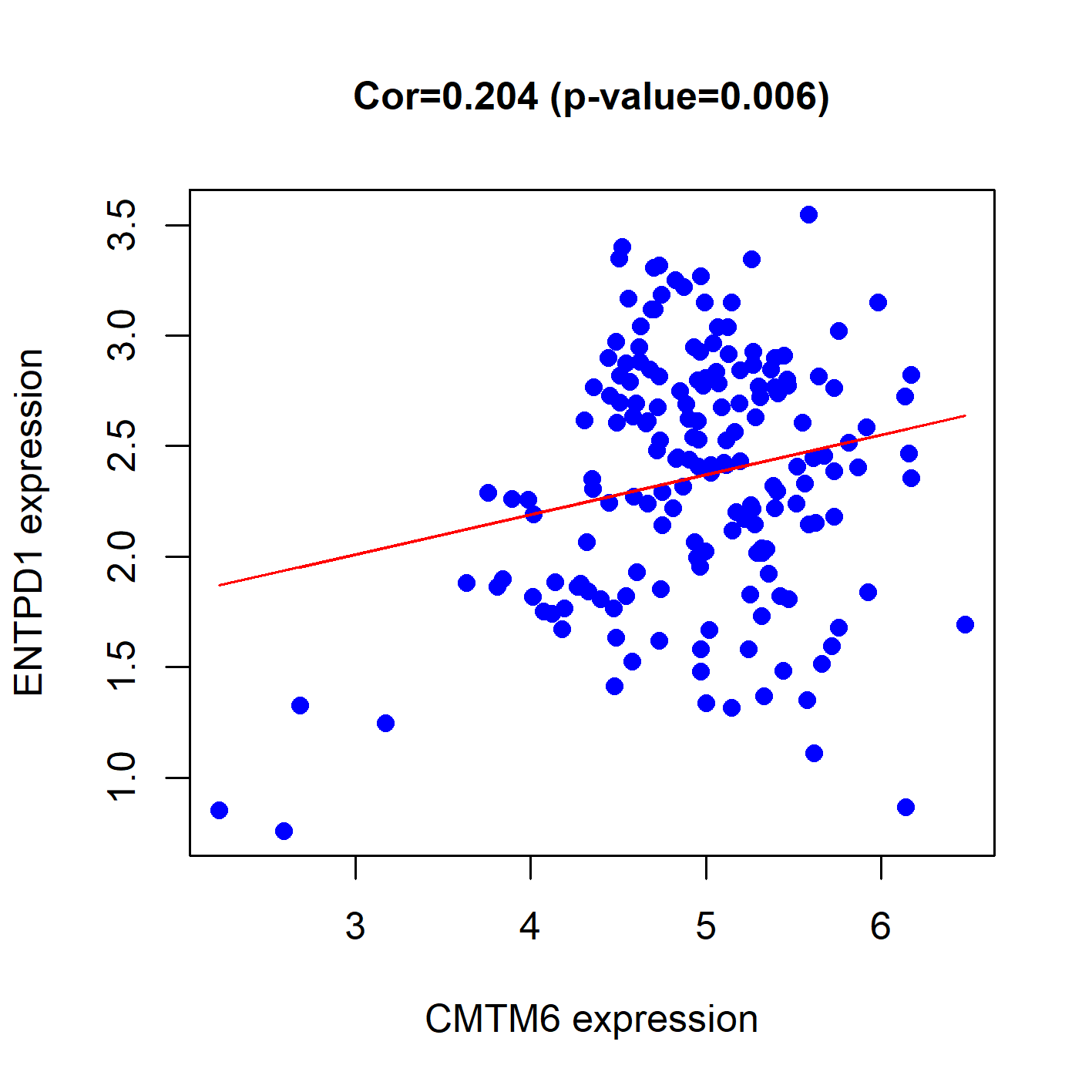

Supplement: Supplementary file 6 — (ZIP 1481 kb) [file 10142_2023_1235_MOESM6_ESM.zip › Supplement File 1/CMTM6_ENTPD1.cor.tiff]

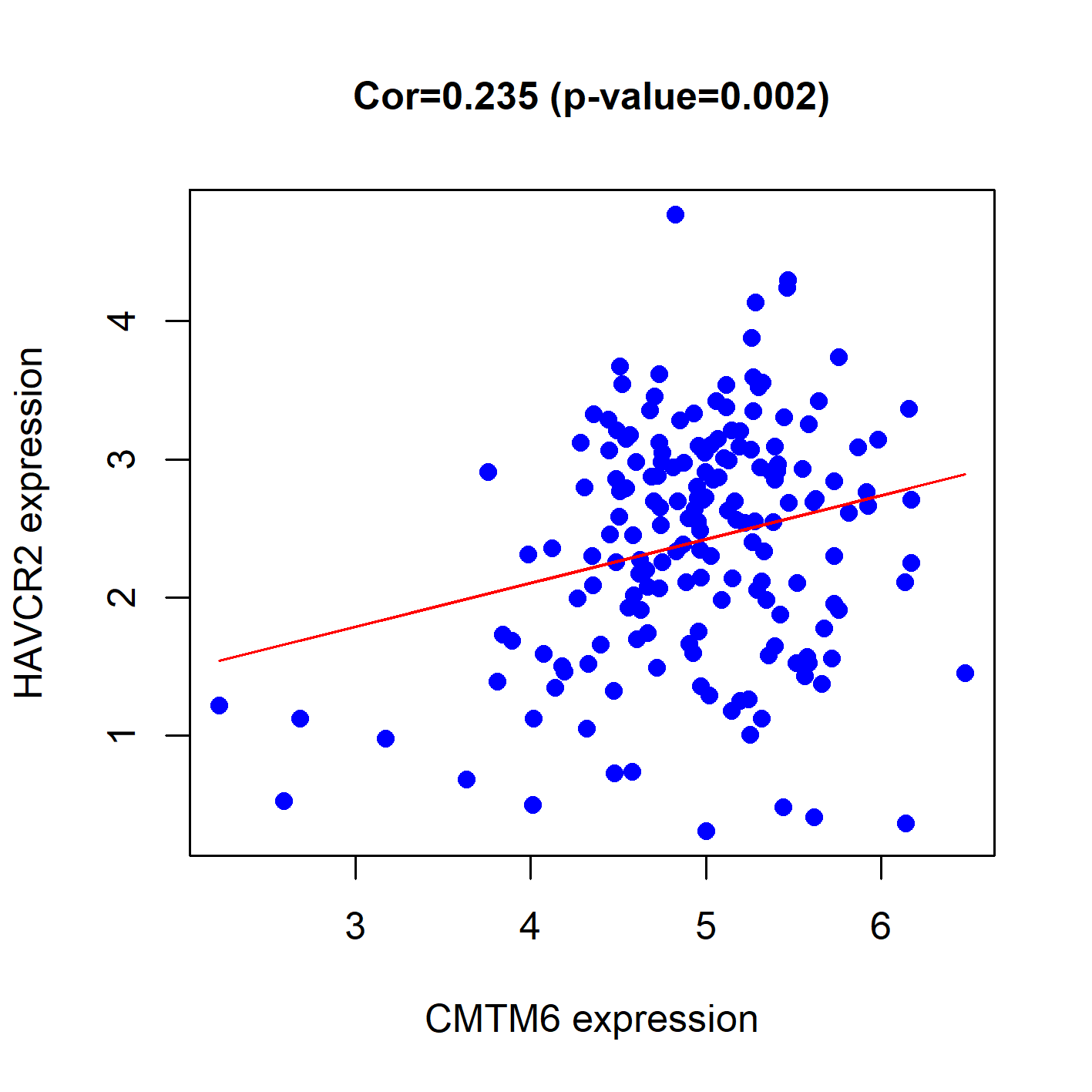

Supplement: Supplementary file 6 — (ZIP 1481 kb) [file 10142_2023_1235_MOESM6_ESM.zip › Supplement File 1/CMTM6_HAVCR2.cor.tiff]

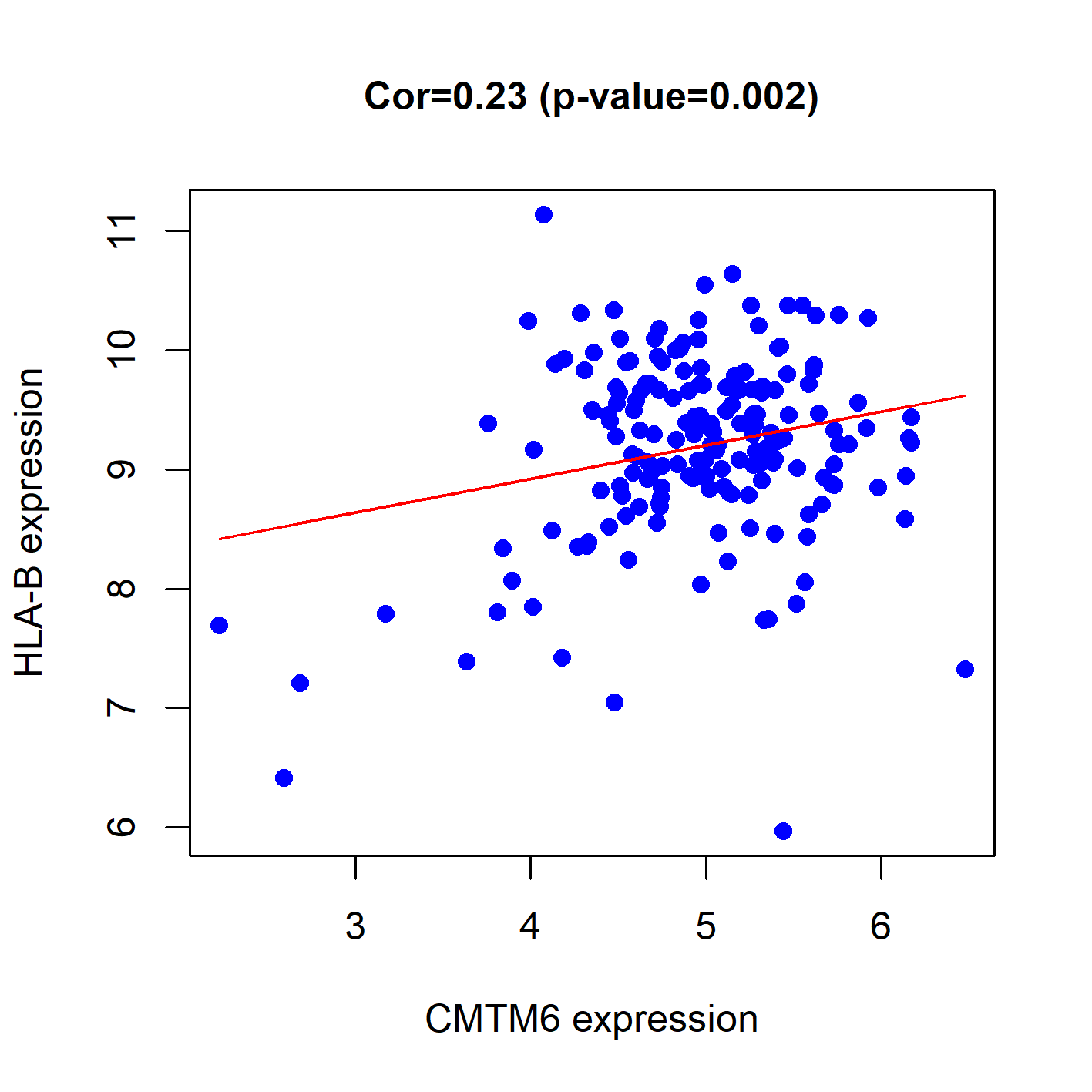

Supplement: Supplementary file 6 — (ZIP 1481 kb) [file 10142_2023_1235_MOESM6_ESM.zip › Supplement File 1/CMTM6_HLA-B.cor.tiff]

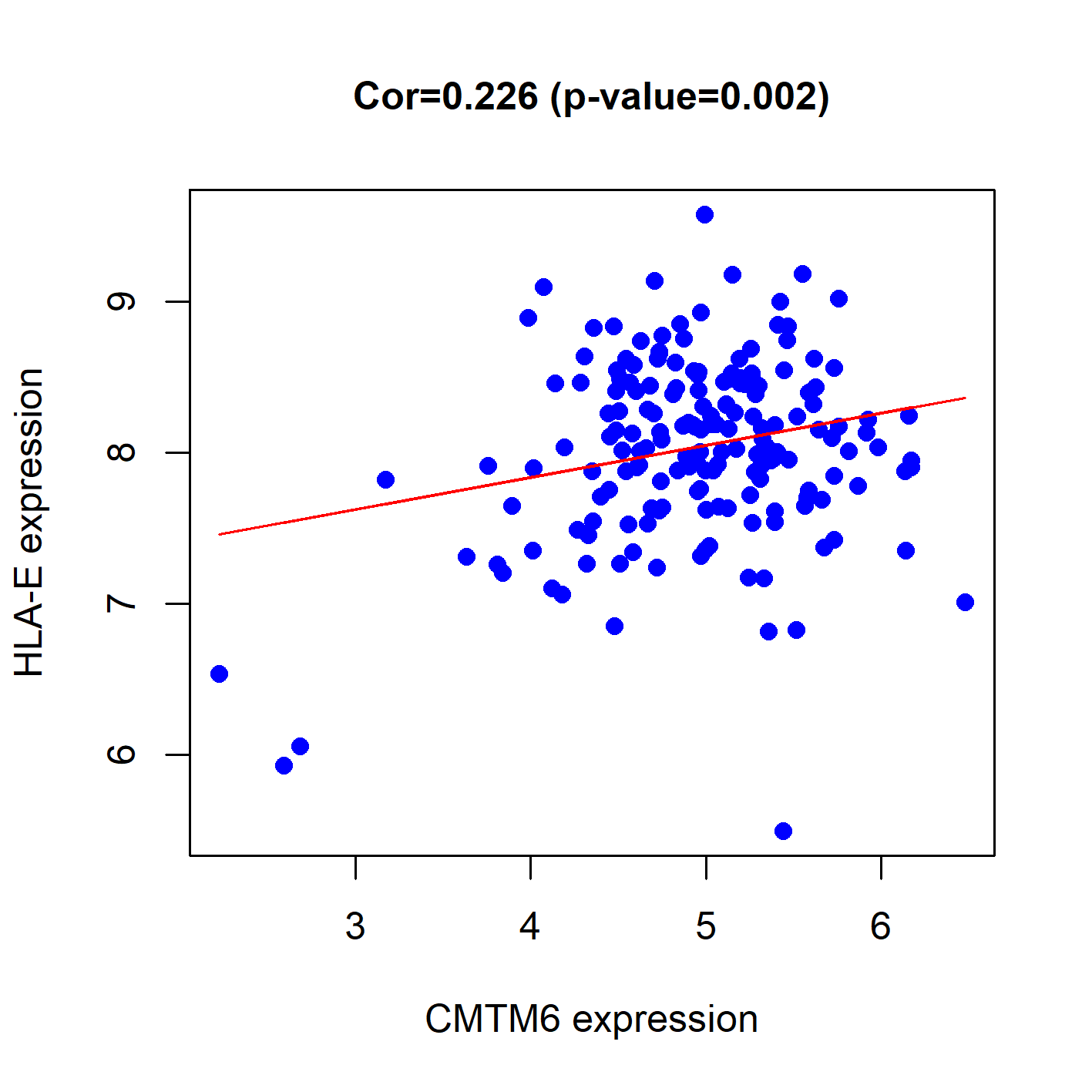

Supplement: Supplementary file 6 — (ZIP 1481 kb) [file 10142_2023_1235_MOESM6_ESM.zip › Supplement File 1/CMTM6_HLA-E.cor.tiff]

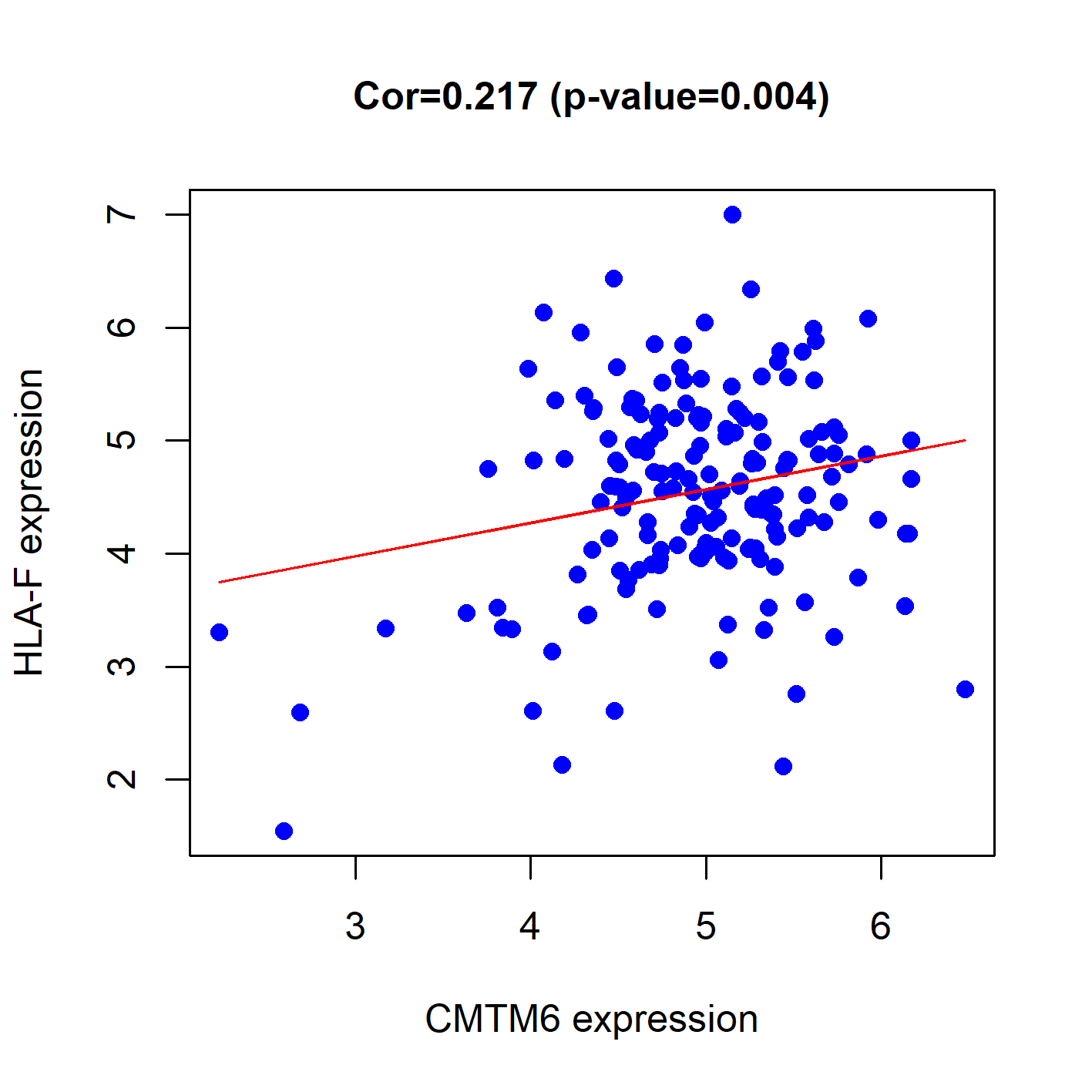

Supplement: Supplementary file 6 — (ZIP 1481 kb) [file 10142_2023_1235_MOESM6_ESM.zip › Supplement File 1/CMTM6_HLA-F.cor.tiff]

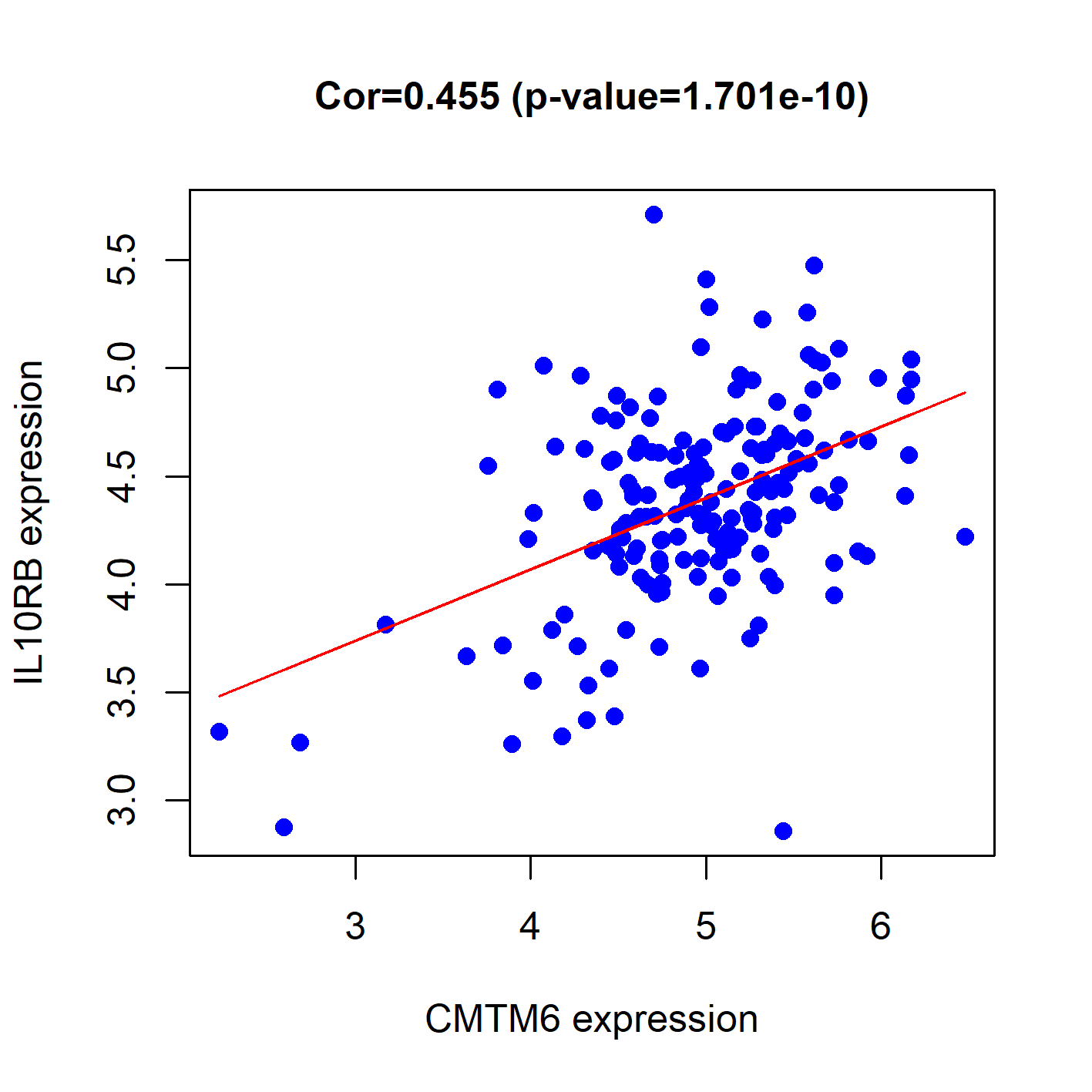

Supplement: Supplementary file 6 — (ZIP 1481 kb) [file 10142_2023_1235_MOESM6_ESM.zip › Supplement File 1/CMTM6_IL10RB.cor.tiff]

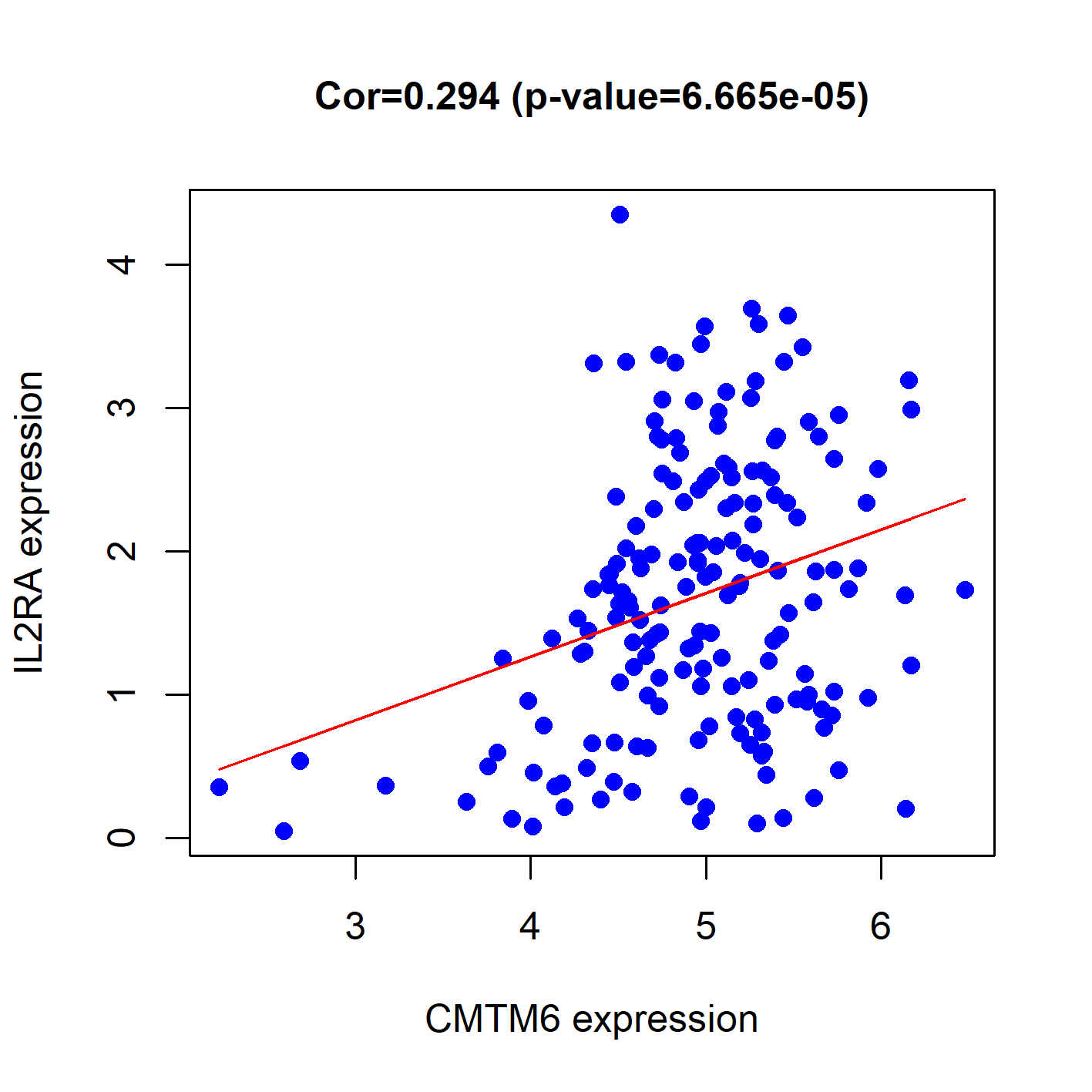

Supplement: Supplementary file 6 — (ZIP 1481 kb) [file 10142_2023_1235_MOESM6_ESM.zip › Supplement File 1/CMTM6_IL2RA.cor.tiff]

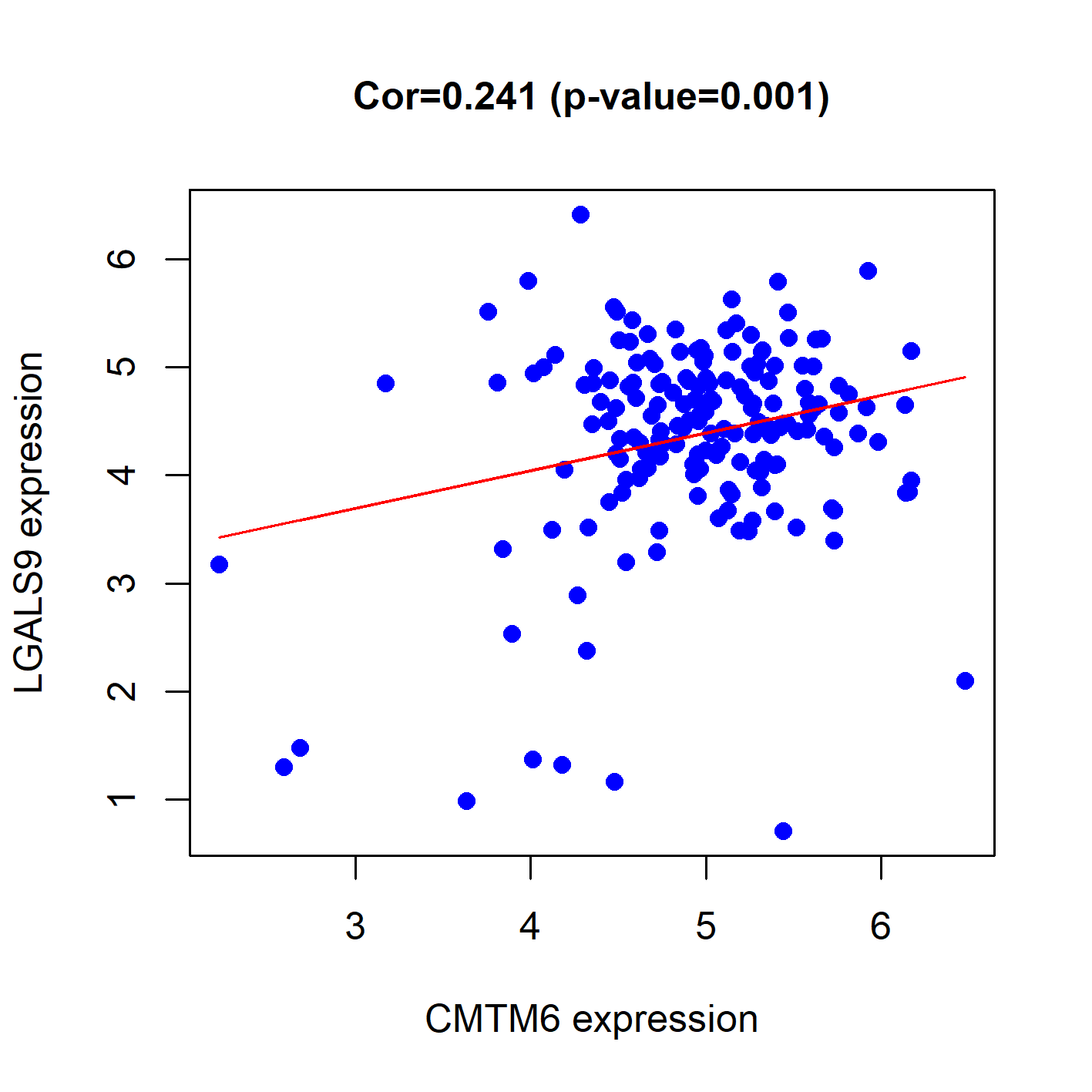

Supplement: Supplementary file 6 — (ZIP 1481 kb) [file 10142_2023_1235_MOESM6_ESM.zip › Supplement File 1/CMTM6_LGALS9.cor.tiff]

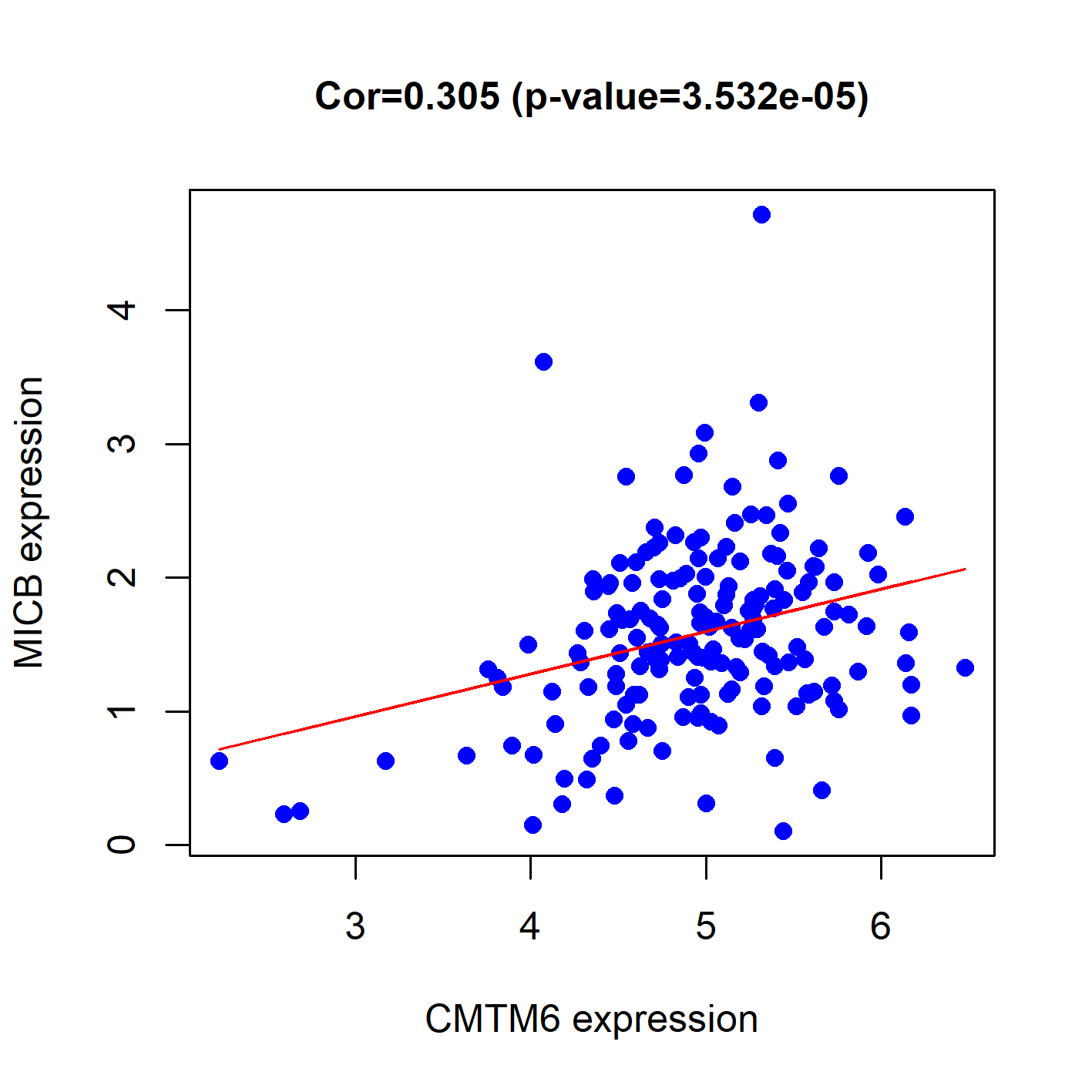

Supplement: Supplementary file 6 — (ZIP 1481 kb) [file 10142_2023_1235_MOESM6_ESM.zip › Supplement File 1/CMTM6_MICB.cor.tiff]

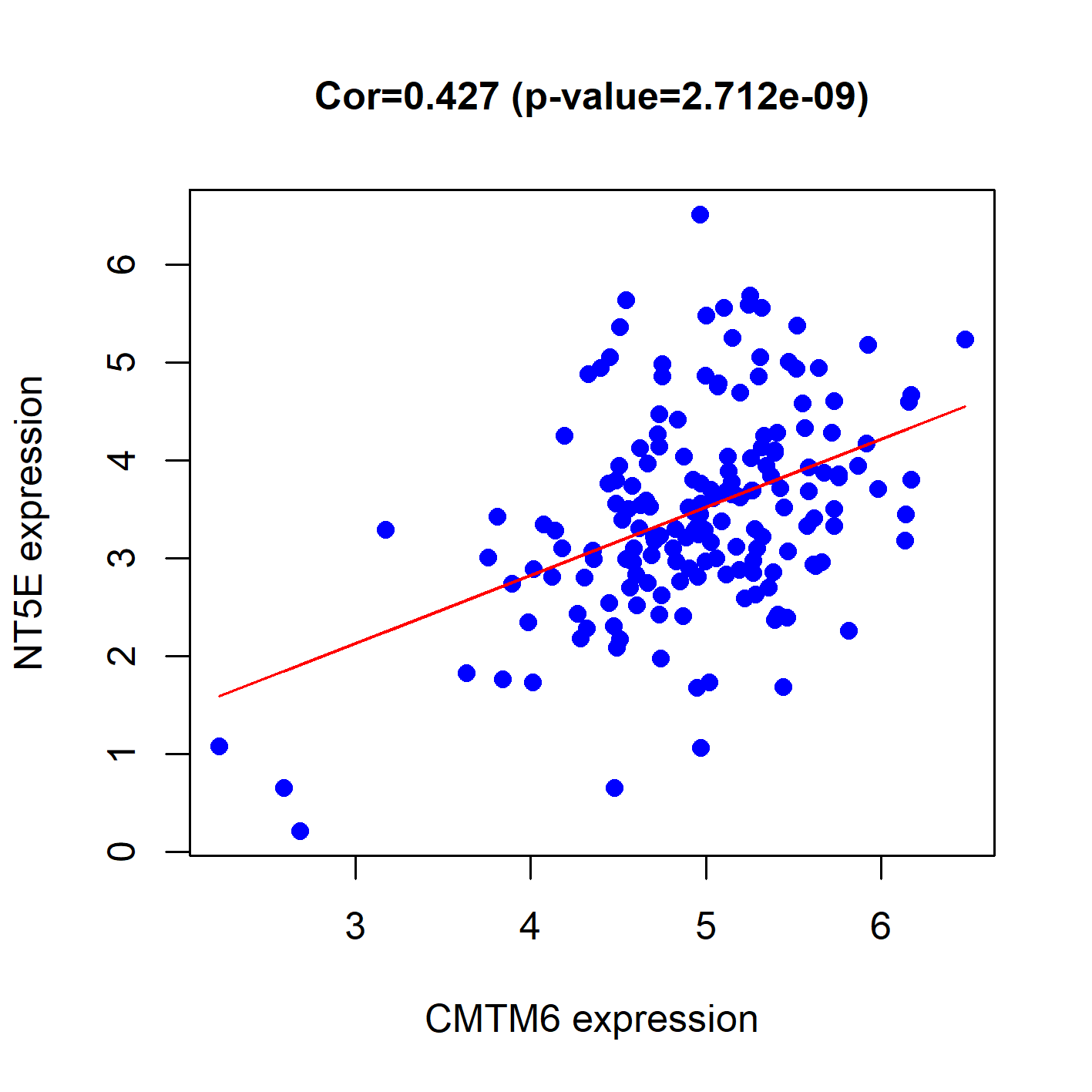

Supplement: Supplementary file 6 — (ZIP 1481 kb) [file 10142_2023_1235_MOESM6_ESM.zip › Supplement File 1/CMTM6_NT5E.cor.tiff]

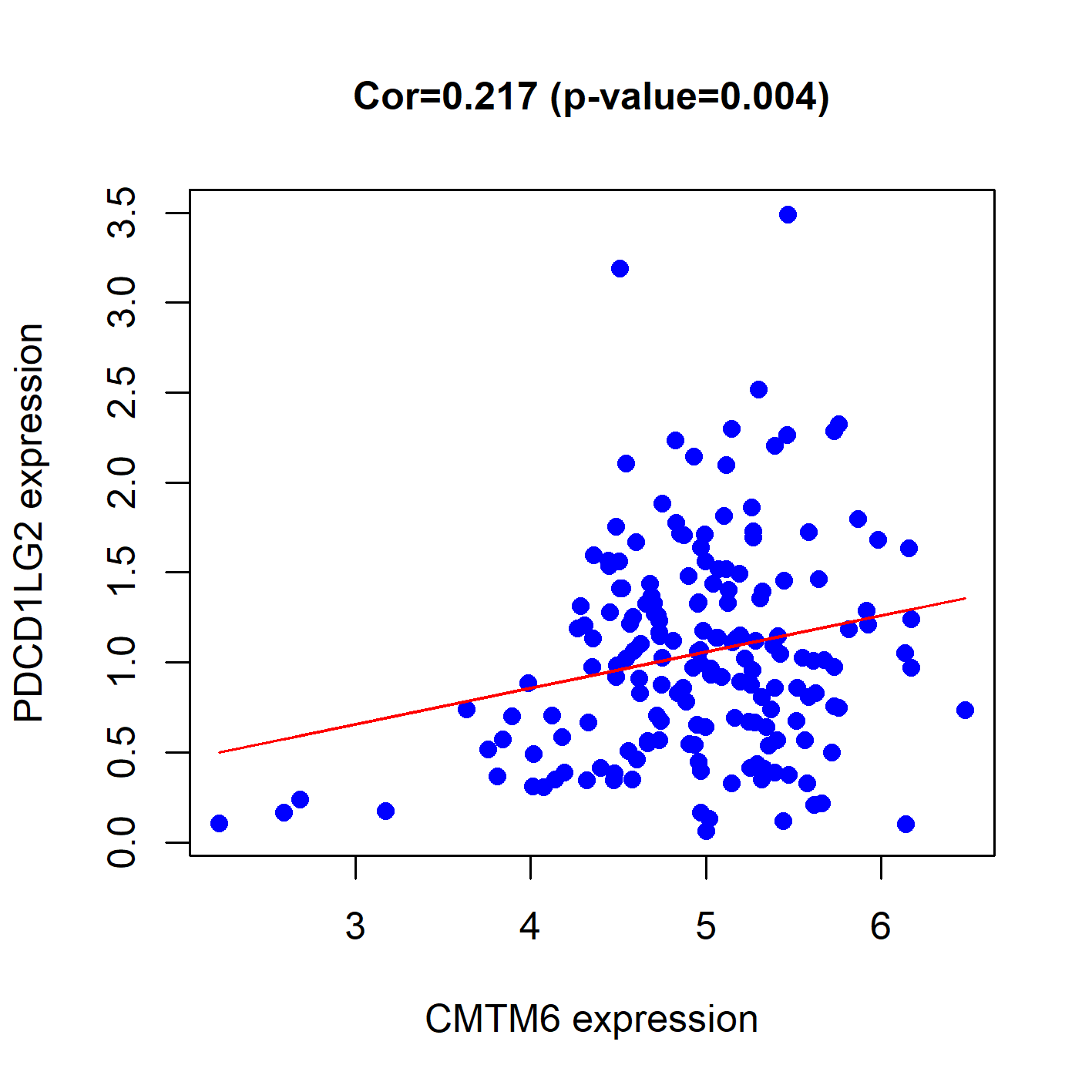

Supplement: Supplementary file 6 — (ZIP 1481 kb) [file 10142_2023_1235_MOESM6_ESM.zip › Supplement File 1/CMTM6_PDCD1LG2.cor.tiff]

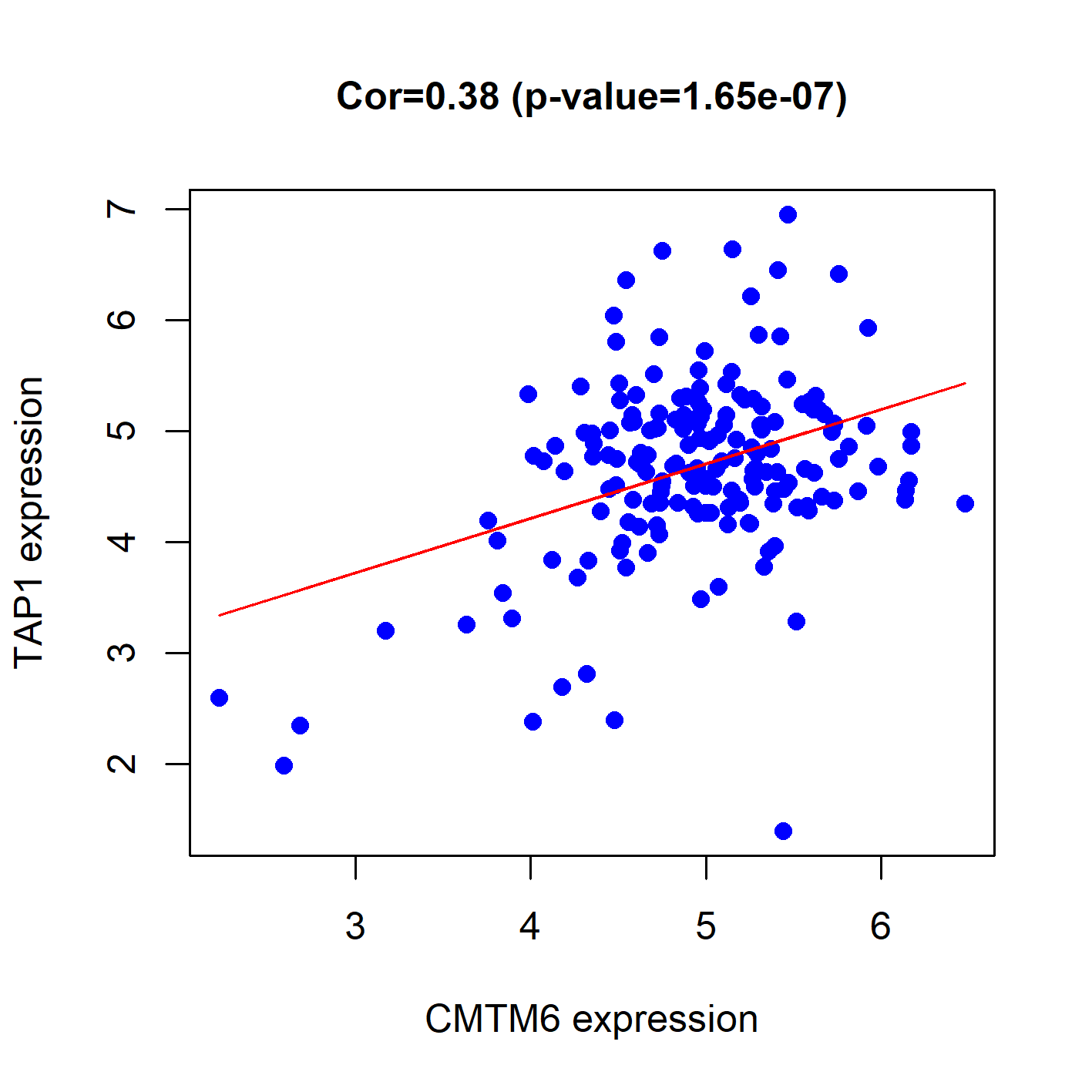

Supplement: Supplementary file 6 — (ZIP 1481 kb) [file 10142_2023_1235_MOESM6_ESM.zip › Supplement File 1/CMTM6_TAP1.cor.tiff]

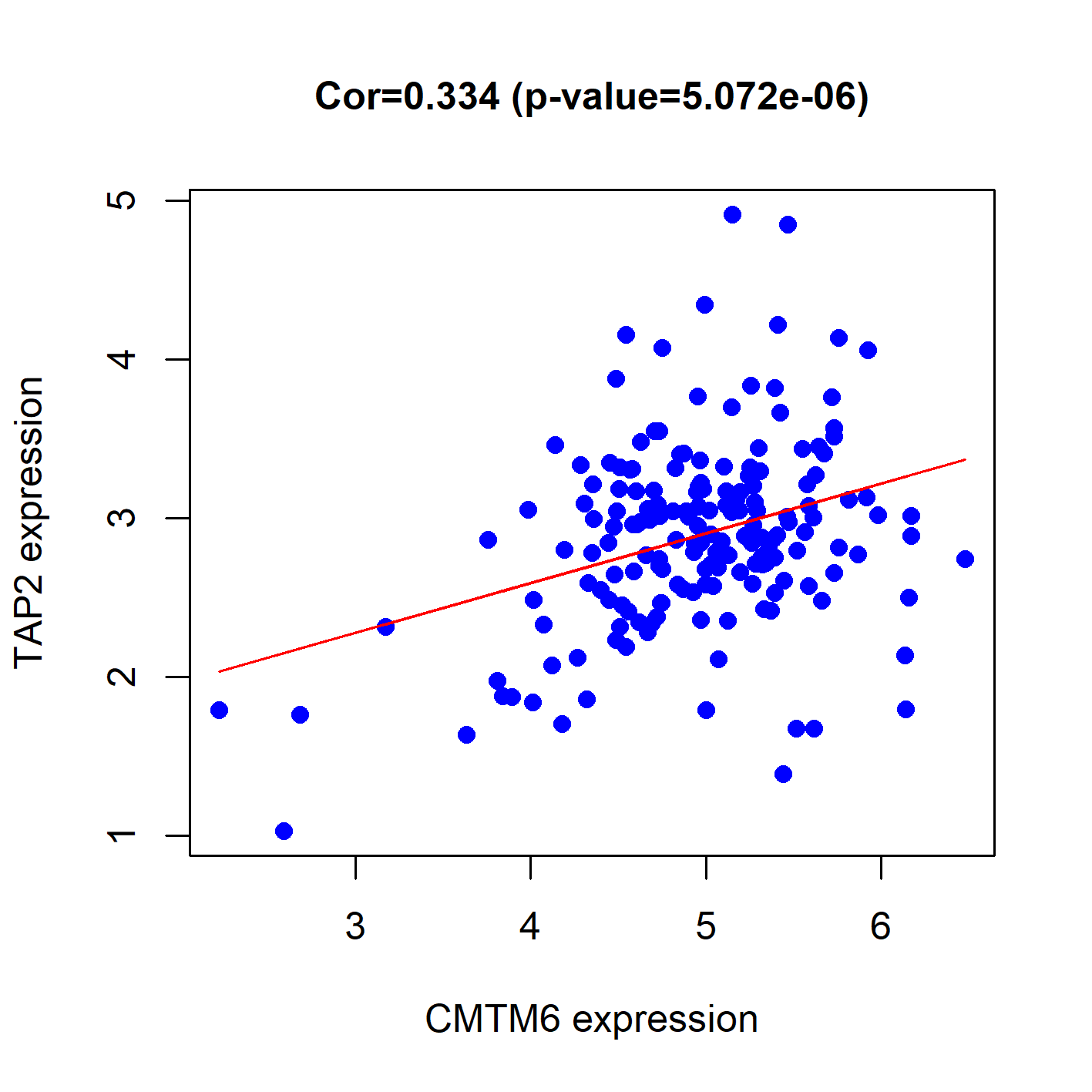

Supplement: Supplementary file 6 — (ZIP 1481 kb) [file 10142_2023_1235_MOESM6_ESM.zip › Supplement File 1/CMTM6_TAP2.cor.tiff]

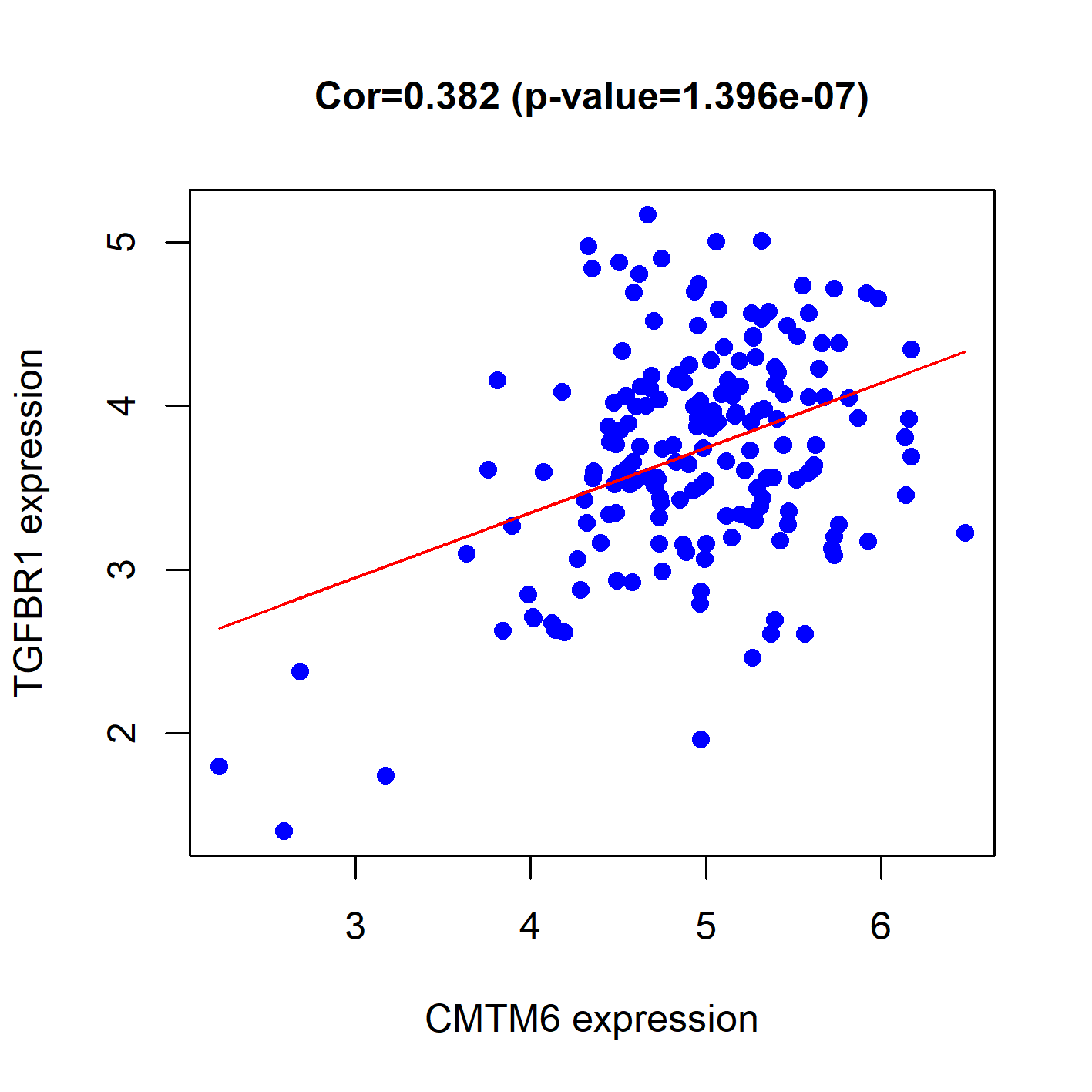

Supplement: Supplementary file 6 — (ZIP 1481 kb) [file 10142_2023_1235_MOESM6_ESM.zip › Supplement File 1/CMTM6_TGFBR1.cor.tiff]

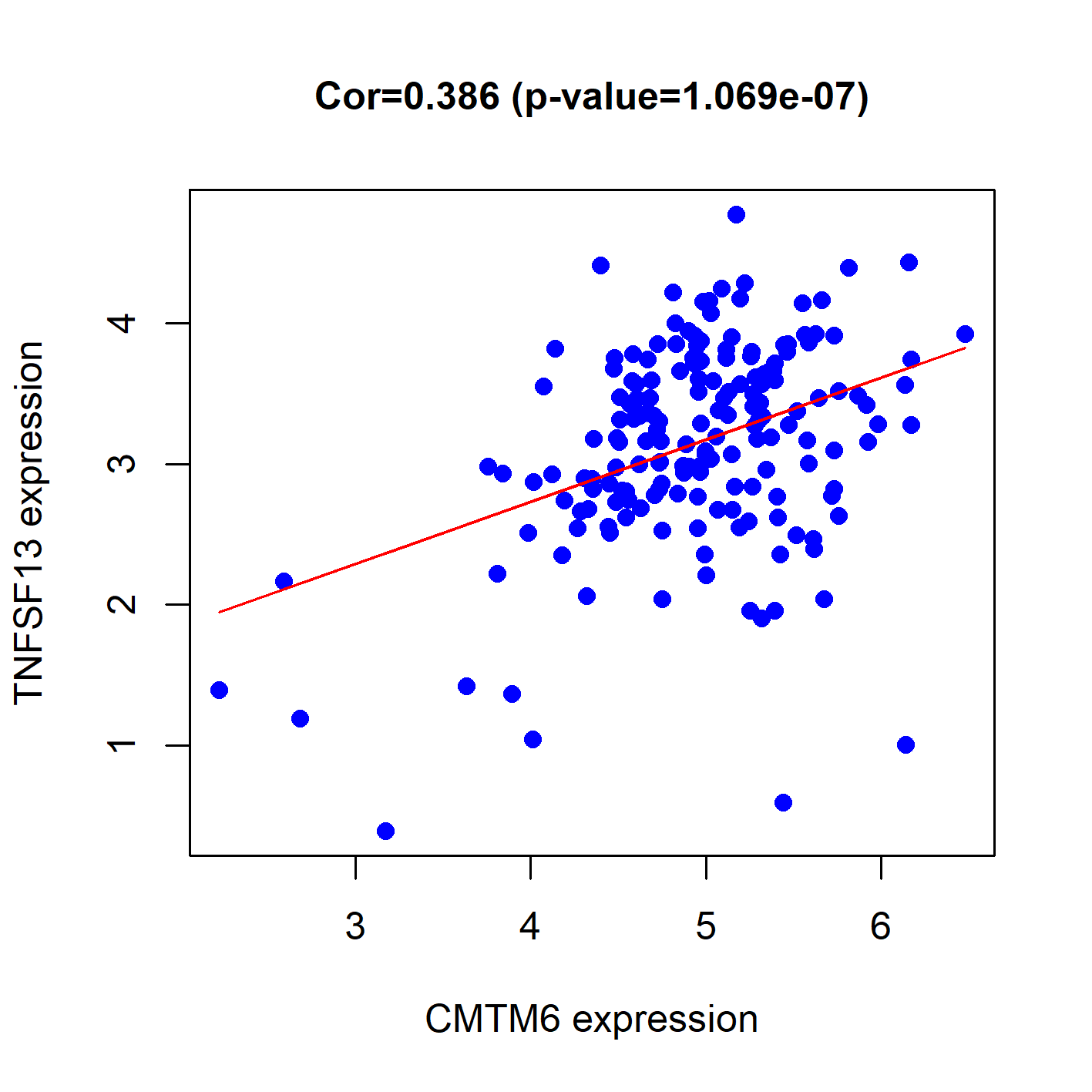

Supplement: Supplementary file 6 — (ZIP 1481 kb) [file 10142_2023_1235_MOESM6_ESM.zip › Supplement File 1/CMTM6_TNFSF13.cor.tiff]

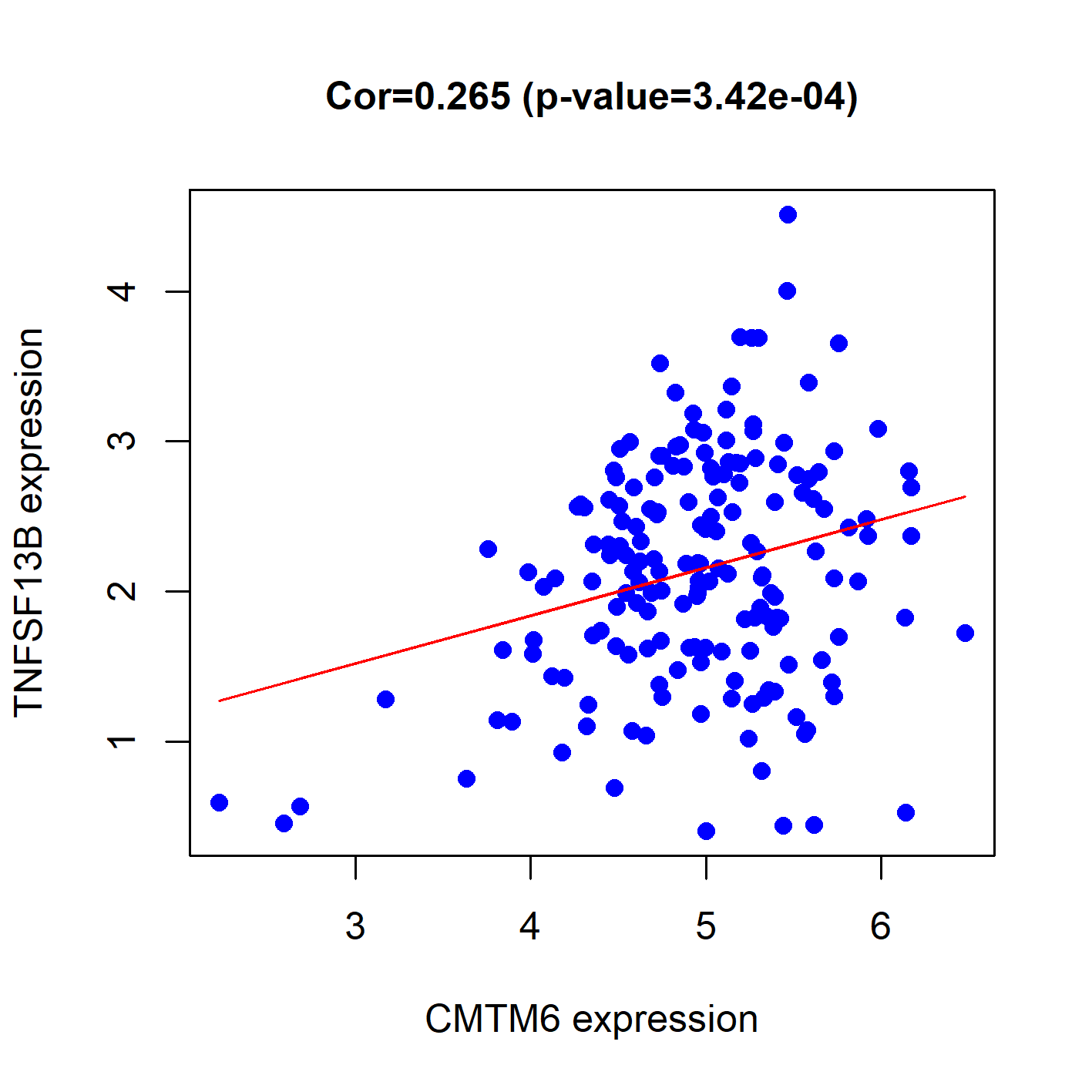

Supplement: Supplementary file 6 — (ZIP 1481 kb) [file 10142_2023_1235_MOESM6_ESM.zip › Supplement File 1/CMTM6_TNFSF13B.cor.tiff]

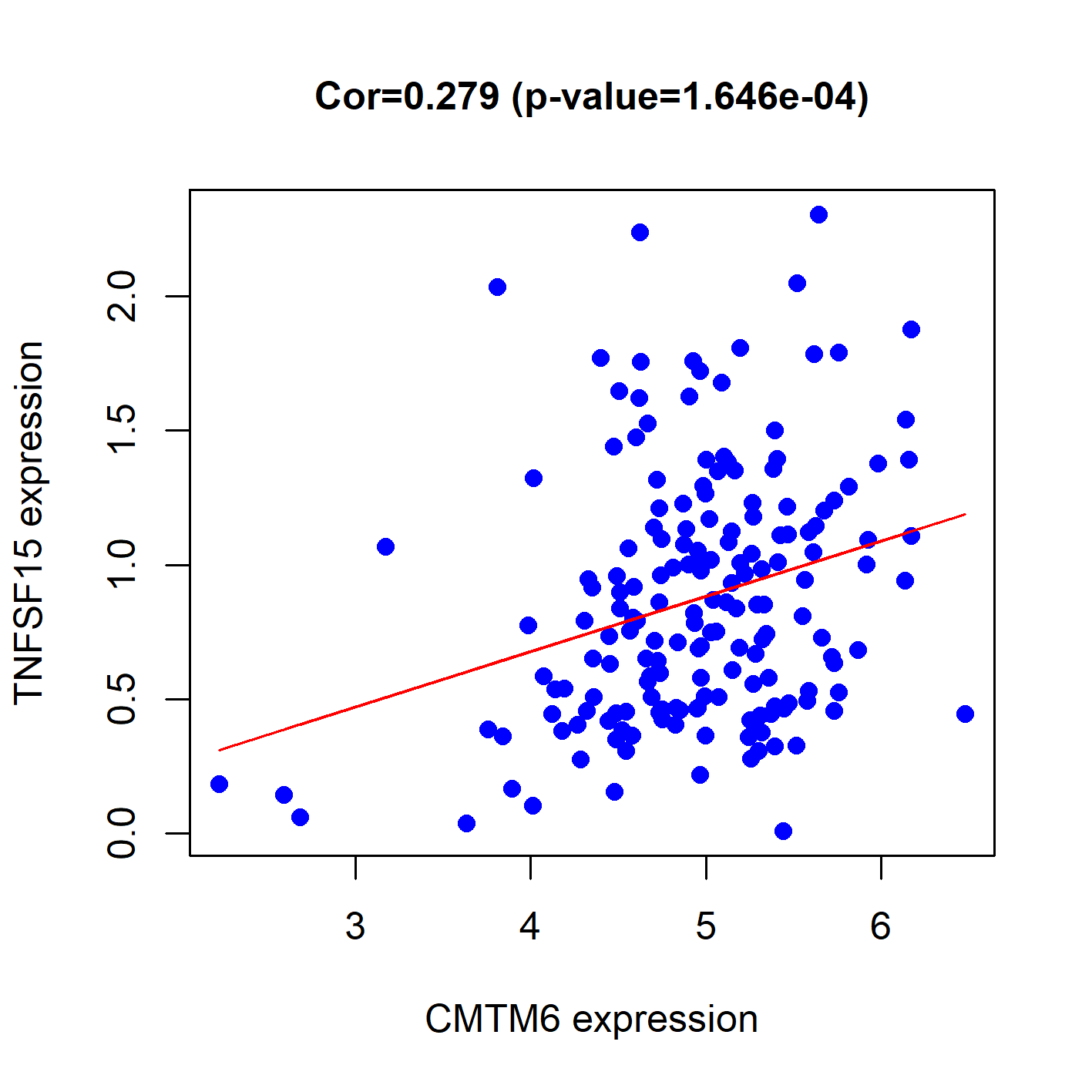

Supplement: Supplementary file 6 — (ZIP 1481 kb) [file 10142_2023_1235_MOESM6_ESM.zip › Supplement File 1/CMTM6_TNFSF15.cor.tiff]

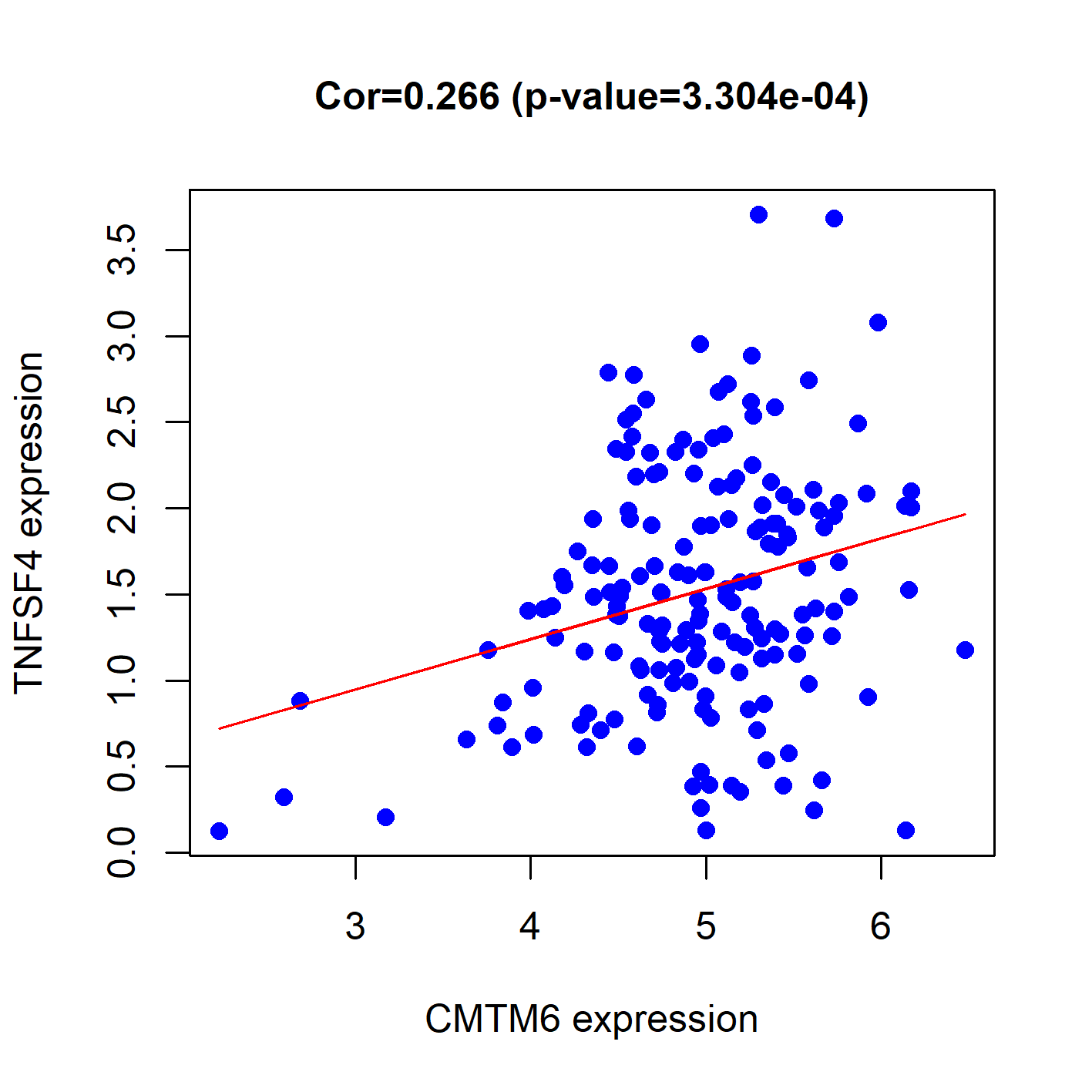

Supplement: Supplementary file 6 — (ZIP 1481 kb) [file 10142_2023_1235_MOESM6_ESM.zip › Supplement File 1/CMTM6_TNFSF4.cor.tiff]
